# Gateway-Driven Hybrid Adaptive Power Management for Energy-Harvesting LPWAN IoT Nodes Using Fuzzy Logic

**DOI:** 10.3390/s26196257

**Published:** 2026-10-02

**Authors:** Mehmet Erkan Yuksel

**Affiliations:** Department of Computer Engineering, Burdur Mehmet Akif Ersoy University, 15200 Burdur, Türkiye; erkanyuksel@mehmetakif.edu.tr

**Keywords:** LPWAN, IoT, energy harvesting, fuzzy logic control, hybrid adaptive power management, gateway-driven synchronization, dynamic power management

## Abstract

**Highlights:**

**What are the main findings?**

- In HA-PM, the gateway fuzzy logic controller determines a nominal node-to-gateway reporting period based on the usable energy state of charge, observed network traffic, and current weather/control context. This enables node-side power-mode and task-scheduling decisions under local service and energy constraints.
- The node selects the power-mode and task-scheduling plan with the lowest estimated total node energy from a defined set of feasible plans that satisfy mandatory sensing, reporting, and timing requirements under the reporting calendar.
- Across the evaluated simulation scenarios, HA-PM improved supercapacitor-level operational continuity and adapted synchronization behavior under adverse energy-harvesting and traffic-load conditions.

**What are the implications of the main findings?**

- Keeping full fuzzy inference at the gateway allows resource-constrained nodes to use adaptive reporting policies while retaining responsibility for command processing and local planning.
- Linking reporting policies with mandatory local service requirements offers a practical basis for energy-management design in energy-harvesting LPWAN IoT systems.

**Abstract:**

Low-Power Wide-Area Network (LPWAN)-based Internet of Things (IoT) deployments require energy management strategies that adapt to dynamic operating conditions without increasing the processing burden on energy-harvesting nodes. These nodes must coordinate data reporting with mandatory local services within a finite energy budget. Conventional fixed duty-cycling and synchronization policies do not jointly account for node energy availability, network traffic, and environmental factors influencing energy harvesting. This study presents hybrid adaptive power management (HA-PM), a framework combining gateway-based reporting-period adaptation with node-side scheduling and power-mode selection. A gateway fuzzy logic controller (FLC) evaluates supercapacitor state of charge (SoC), network traffic, and weather/control context to adjust the reporting period. The node selects the feasible plan with the lowest estimated total node energy from a finite catalog, subject to mandatory service and timing requirements. The reporting period specifies data-reporting requirements, while the local schedule determines available low-power intervals. Simulations compare HA-PM with fixed-period baselines across representative deployment, workload, harvesting, and adverse-condition scenarios. Results indicate that HA-PM reduces communication-related energy consumption, increases low-power-mode residency, and improves supercapacitor-level operational continuity under the evaluated conditions. Stress tests further demonstrate adaptive responses to adverse harvesting and traffic scenarios. These findings support HA-PM as a practical, context-aware energy management strategy for energy-harvesting LPWAN IoT applications, although the reported results are contingent on the specified simulation models, parameters, and operating scenarios.

## 1. Introduction

LPWAN technologies support long-range, low-data-rate communication for geo-graphically distributed IoT devices with limited energy and communication resources [1,2]. They complement short-range technologies such as Wi-Fi, Zigbee, and Bluetooth in applications where communication range, infrastructure density, and maintenance frequency are important design constraints [3,4]. In deployments requiring extended unattended operation of devices, communication scheduling and power-state management substantially influence node energy consumption and service life [5,6].

Energy-harvesting wireless sensor nodes, including those used in LPWAN deployments, seek to extend unattended operation by combining ambient-energy sources with local storage elements, such as supercapacitors [7,8]. The available energy varies with the harvesting source, storage state, workload, and operating conditions. A fixed wake-up or synchronization schedule may therefore consume energy unnecessarily under energy-constrained conditions or may be overly conservative when the stored energy and application context permit more frequent activity [9]. This energy–responsiveness trade-off motivates adaptive power management policies that account for both energy availability and application requirements [10,11].

Research on LPWANs and energy-harvesting sensor nodes has considered fuzzy adaptive data-rate mechanisms [12], adaptive transmission-interval and duty-cycle policies [13], energy-harvesting-aware scheduling and network-assisted resource management [14,15], and evolutionary and digital-twin-assisted fuzzy energy management approaches [16,17]. These studies adapt radio parameters, reporting intervals, scheduling decisions, or node-side duty-cycle controllers. Adapting the reporting period changes the time available for local tasks and low-power operation, so the node must select a feasible mode and schedule that meets its mandatory sensing, reporting, and timing requirements. This study examines how gateway-side context assessment and local power-mode selection can be coordinated under explicit sensing, reporting, and timing requirements.

HA-PM adapts the node reporting period to operating conditions through a gateway FLC. The node selects the power-mode and task-scheduling plan with the lowest estimated total energy consumption among the candidates that satisfy the required sensing, reporting, and timing conditions. The gateway determines the nominal node-to-gateway reporting and synchronization period, $T_{sync}$. The node selects a feasible mode and schedule under that reporting calendar and its mandatory local work; $T_{sync}$ is neither a power-mode command nor a sleep duration.

This study addresses the following research questions:

- RQ1: How does the gateway FLC adapt the nominal reporting period to supercapacitor SoC, observed network traffic load, and weather context?
- RQ2: For a given reporting calendar, which feasible operating-mode and task-scheduling plan minimizes estimated total node energy when candidates are compared for the same mandatory jobs over a common horizon and under equivalent terminal conditions?
- RQ3: Under the specified simulation model, how do the reporting policy (fixed-period or FLC-based) and the node-level selection policy (baseline or proposed) affect energy demand, service delivery, energy-storage dynamics, and time to depletion when evaluated using the same energy-harvesting inputs, event-arrival sequences, and external channel conditions?

The main contributions of this study are as follows:

- A coordinated gateway-node power management architecture that performs context processing at the gateway while retaining power-mode and task-scheduling plan selection at the node.
- A Mamdani FLC that determines the nominal node-to-gateway reporting period from supercapacitor SoC, observed network traffic load, and weather context.
- A node-side controller that selects the plan with the lowest estimated total node energy requirement from a defined finite set of feasible operating-mode and task-scheduling plans, all evaluated for the same mandatory jobs over a common time horizon and under equivalent terminal conditions.
- A reproducible numerical model of the controller and node planner, with explicit assumptions and constraints regarding energy accounting, service requirements, command processing, and energy-storage dynamics.

The remainder of this paper is organized as follows. Section 2 reviews the relevant literature. Section 3 describes the proposed method and experimental protocol. Section 4 presents the evaluation metrics and quantitative evidence. Section 5 discusses the scope and limitations, and Section 6 concludes the paper.

## 2. Related Work

### 2.1. LPWAN Adaptation and Network-Side Control

LPWAN technologies provide long-range connectivity at low data rates and with constrained device energy budgets. Individual technologies differ in spectrum use, link budget, data rate, latency, capacity, and network organization, so their suitability depends on the application and deployment conditions [1,2,3,4]. In LoRaWAN, duty-cycle restrictions, shared-channel contention, receive-window operation, and limited downlink capacity constrain how frequently end devices and gateways can communicate [18]. These characteristics make communication timing and radio configuration important aspects of energy-aware system design.

Adaptive data rate (ADR) mechanisms primarily address the radio link by modifying transmission parameters in response to observed link conditions. Kufakunesu et al. placed a fuzzy ADR controller at the LoRaWAN network server and used recent link observations to assign the spreading factor and transmission power [12]. Gupta et al. used a gateway-side Mamdani controller whose inputs were energy consumption, packet-delivery ratio, throughput, and delay. The direct fuzzy output was predicted network efficiency, which was subsequently used to guide spreading-factor, bandwidth, and coding-rate settings [19]. Both approaches externalize part of the decision process from the end device, but their control objectives and downstream actions concern the radio-link configuration rather than a nominal node-to-gateway reporting period (communication timing) used in local power-mode and task-scheduling decisions.

Cross-layer control extends the decision space beyond conventional ADR. Wang et al. combined fuzzy cognitive maps with adaptive glowworm swarm optimization to model interacting energy-harvesting LPWAN factors and support cross-layer multi-objective resource management [14]. This work provides a relevant precedent for cross-layer fuzzy resource management. HA-PM uses a predefined Mamdani controller for reporting-period adaptation and connects this output to node-side selection of a feasible operating plan based on estimated total energy.

Beletsioti et al. proposed Gateway Energy-Oriented TDMA (GEOT), in which the network server associates end nodes with gateways and constructs per-gateway schedules from the gateway and node populations, measurement periodicity, and spreading-factor assignments [20]. Gateways listen during scheduled broadcast and reception windows and enter the sleep mode during the remaining slots. Simulations examined the gateway lifetime and transmission time under different node-placement distributions. GEOT therefore addresses communication scheduling and operating states at renewable-powered gateways. HA-PM places the local energy selection decision at the node, while the gateway adapts the reporting period that constrains the node’s planning problem.

### 2.2. Energy-Harvesting-Aware Activity and Communication Timing

Energy-harvesting nodes must balance device energy demand against time-varying harvested supply and the energy available in storage. Kansal et al. formulated harvesting-aware power management by allocating node duty cycles to maximize application utility under predicted time-varying harvested energy and battery constraints [21]. Fuzzy duty-cycle control provides one implementation of this principle. Mothku and Rout used current residual energy, predicted harvested energy, and predicted residual energy to determine the node duty cycle in an energy-harvesting sensor-actor network [13]. This work links fuzzy inference directly to the regulation of node activity.

Capacitor-based and batteryless LoRaWAN systems introduce additional constraints because sensing or transmission may proceed only when sufficient stored energy is available. Sabovic et al. examined capacitor-aware scheduling of sensing and transmission tasks and compared sleep-based and power-off operating strategies [8]. Gléonec et al. computed a consumable energy budget for a multi-source energy-harvesting LoRaWAN node and allocated it across sensing tasks according to their energy costs, priorities, and quality-of-service bounds [10]. Giuliano et al. subsequently considered adaptive data transmission intervals for battery-free LoRaWAN sensors [22,23]. These studies illustrate how energy availability can inform task execution, operating strategy, energy allocation, and transmission timing. Task scheduling and transmission-interval adaptation are relevant to HA-PM; the comparison concerns where decisions are made, which information is used, and how reporting period decisions are connected to feasible local operating plans.

Gerndt et al. implemented energy-aware duty-cycle management for a solar-powered IoT device using a low-cost solar control board integrated with the Serverless IoT Framework [24]. Their case study proactively adjusted the device sleep time according to predicted available solar energy over a sequence of cloudy days. The controlled variable was device activity timing. In HA-PM, the gateway adapts the nominal reporting period, while the node determines its operating plan subject to that reporting calendar and its mandatory local tasks.

Network-assisted energy control has also been used to coordinate access among harvesting-powered devices. Michelusi and Levorato placed the access decision at the base station, which adapted transmission probability using harvesting-state information and an estimate of the active-node population [25]. This policy controls when nodes attempt channel access. HA-PM uses a reporting-period output to inform node-side power-mode and task-scheduling plan selection, rather than directly setting an access probability.

The literature therefore contains several forms of harvesting-aware temporal adaptation, including duty-cycle control, capacitor-threshold scheduling, task-energy allocation, transmission-interval adaptation, and base-station-managed access probability. These methods are related through their use of energy information, but they differ in decision locus, controlled output, traffic assumptions, storage treatment, and evaluation boundary. Their published numerical results should consequently not be treated as directly comparable measures of a single control problem.

### 2.3. Node-Side Dynamic Power Management

Dynamic power management (DPM) aims to reduce energy demand by dynamically reconfiguring a system so that idle or partly utilized components can be turned off or operated at reduced performance while the required services and performance levels are maintained [26]. Changing a communication interval and selecting a hardware power state are therefore distinct decisions. The interval constrains scheduling opportunities, while the actual time available for low-power operation also depends on sensing, reporting, and other mandatory tasks. Miao et al. combined a node-mode switching strategy, under which sensor nodes execute tasks according to their current energy level, with an adaptive transmission-threshold strategy that also accounts for data importance [27]. This control path connects the node energy state and data importance with task execution and transmission decisions. Yuksel and Fidan presented an energy-aware architecture for batteryless LPWAN devices that combines solar harvesting, supercapacitor storage, heterogeneous communication interfaces, and power-aware embedded software based on component power modes [7]. This architecture provides a device-side precedent for multi-mode energy management.

In HA-PM, the gateway determines the nominal node-to-gateway reporting period, $T_{sync}$, while the node selects its power mode and task schedule. At each planning decision, the node compares a defined finite set of feasible plans that meet the same mandatory sensing, reporting, and timing requirements over a common horizon and under equivalent terminal conditions. It selects the plan with the lowest estimated total node energy, including power-state transition costs and node-controller overhead. The gateway’s reporting-period output is neither a direct power-mode command nor a sleep duration. The resulting energy minimum is relative to the evaluated candidate set and energy model; it does not establish the globally minimum realized energy consumption.

### 2.4. Fuzzy, Learning-Based, and Distributed Energy Management

Fuzzy energy-management methods also differ in how their rules are obtained and where inference is executed. Hsu et al. combined fuzzy inference with Q-learning and used the fuzzy inference system to determine the node duty cycle [28]. Prauzek et al. developed an evolutionary fuzzy-rule controller in which genetic programming selects the controller structure and inputs, while the resulting controller determines the next transmission period [16]. Their later digital-twin-based work used differential evolution to optimize a fuzzy energy management controller [17]. These methods use learning or evolutionary optimization to construct or tune their controllers. HA-PM adopts a predefined Mamdani rule base, which represents a different controller-design choice rather than an inherent performance advantage.

Kufakunesu et al. incorporated a Mamdani fuzzy inference system into the simulated end-device traffic generation process for LoRaWAN-based military health monitoring [29]. Body temperature, blood pressure, oxygen saturation, and heart rate are combined into a continuous health status score, which a sigmoid mapping converts to an adaptive transmission delay. The method was evaluated in NS-3 against no-flow-control and static-priority schemes under changes in network density and reporting interval. This work is a relevant precedent for fuzzy adaptation of application-data transmission timing. HA-PM uses energy, traffic, and weather context at the gateway to adapt the nominal reporting period and connects that output to local operating plan selection based on estimated total energy.

Distributed frameworks divide energy management functions between nodes and external infrastructure. RACEME combines sensor-side embedded machine learning for harvesting prediction with server-side online cluster-based data reduction and recovery; server feedback informs probabilistic adjustment of LoRa transmission activity [30]. This division of prediction, processing, and feedback provides a relevant comparison for the allocation of control functions between nodes and external infrastructure.

A separate line of work examines the implementation cost of fuzzy controllers. Mikuš et al. measured the evolutionary fuzzy-rule controller execution time on an embedded target and obtained RAM and flash occupancy from the compiler output under multiple implementation, compiler-optimization, and FPU configurations [31]. These measurements characterize that controller implementation on the tested platform; they do not provide resource measurements for HA-PM.

Ruíz-Guirola et al. considered base-station-controlled duty cycling and selective wake-up in event-driven, energy-harvesting IoT networks [32]. Their K-nearest-neighbor, Q-learning, and decision-transformer strategies use the modeled device state, battery availability, event-related sensing information, and spatial activity correlations to determine device activity and wake-up actions. Evaluation was limited to numerical simulation of device-state, stochastic energy-arrival, and battery dynamics. This work provides a relevant comparison for network-assisted activity control. In HA-PM, the gateway determines a nominal reporting period, and the node uses the resulting reporting calendar when selecting a feasible power-mode and task-scheduling plan based on estimated total energy.

Together, these studies show that node-local fuzzy duty-cycle decisions, evolutionary fuzzy-rule control, digital-twin-assisted optimization, sensor-side prediction with server feedback, and network-side fuzzy inference are established approaches. Meaningful comparison therefore requires attention to the information processed at each layer, the variable being controlled, and the connection between that variable and node operation. These dimensions provide the basis for positioning HA-PM.

### 2.5. Research Gap and Positioning of HA-PM

The studies reviewed above cover fuzzy ADR, harvesting-aware duty cycling, capacitor-aware task scheduling, data-transmission-interval adaptation, base-station access control, cross-layer resource management, multi-mode DPM, learning-based fuzzy policies, and distributed node-server energy management. These approaches establish relevant foundations for adapting communication and device activity to energy availability and application demand. For nodes operating under an adaptive reporting period, the design problem addressed here is how to connect gateway-level context assessment with local operating plan selection while making service feasibility and the estimated total energy cost explicit.

HA-PM addresses this problem by coordinating two decision levels. A gateway-side FLC uses the supercapacitor SoC, observed network traffic, and the weather context to determine the nominal node-to-gateway reporting period. For a given reporting calendar, the node selects the power-mode and task-scheduling plan with the lowest estimated total node energy from a defined finite set of feasible candidates. The focus is the coordination of reporting period adaptation and local plan selection under explicit sensing, reporting, and timing requirements. Fuzzy inference, external computation, adaptive timing, and DPM are established components of this design, rather than individually new techniques.

The reviewed studies serve as architectural and methodological comparators. Their published numerical results are not directly interchangeable because their objectives, workloads, storage assumptions, channel models, energy-accounting boundaries, and validation modalities differ. Quantitative comparisons require a common evaluation protocol and clearly stated adaptations. The evidence reported for HA-PM is interpreted within the specified simulation model and assumptions; it does not establish hardware-level, prototype, field, or deployment performance.

## 3. Materials and Methods

### 3.1. System Overview

HA-PM is a gateway-assisted energy management framework for energy-harvesting LPWAN nodes operating under variable energy availability and traffic conditions. At the gateway, a fuzzy logic controller adapts the nominal reporting period, $T_{sync}$, using supercapacitor SoC from delivered node reports, network traffic observed at the gateway, and weather/control-context information. Using the applied reporting period and its mandatory sensing, processing, reporting, and timing requirements, the node assesses the feasibility of power-mode and task-scheduling plans from a defined finite catalogue. Among feasible candidates, the node selects and executes the plan with the lowest estimated total node energy, including the costs of task execution, mode transitions, and node-side computation for control and planning. The selected schedule determines the available low-power intervals. In the numerical evaluation, scenario traces prescribe the weather/control context and harvesting inputs, while a separate declared model represents the channel.

### 3.2. Node and Service Model

**Figure 1:**
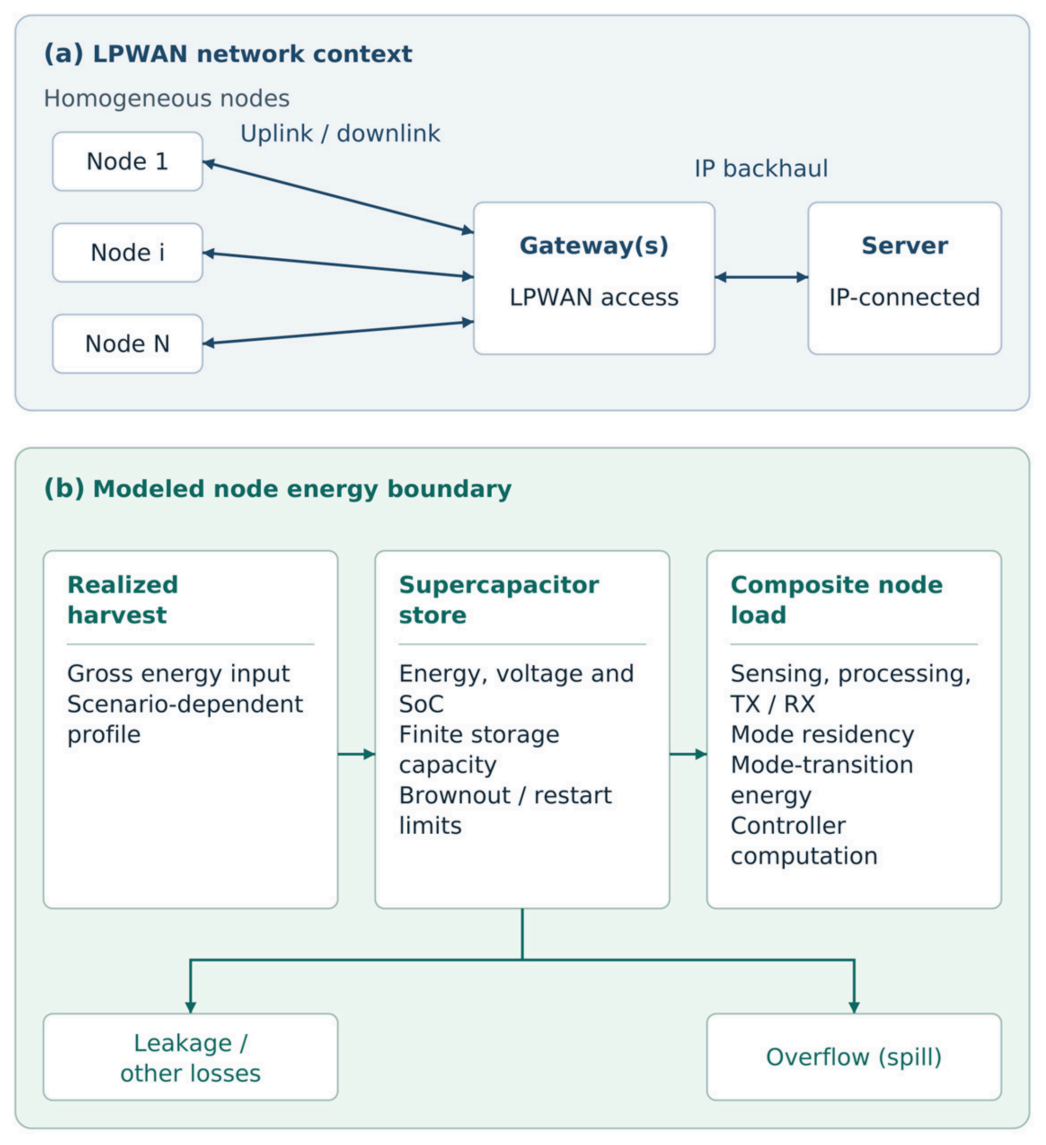
LPWAN network context and modeled node energy boundary.

**Figure 2:**
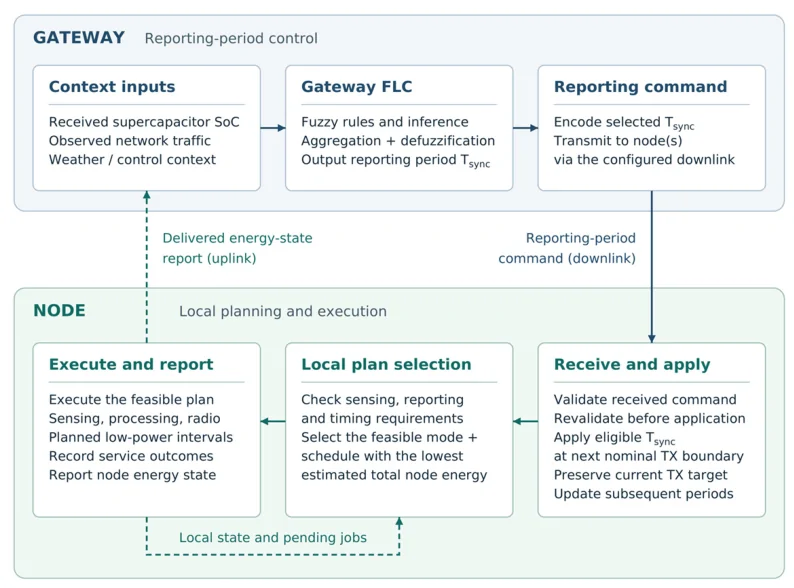
Gateway-assisted reporting-period control and local node planning in HA-PM.

Figure 1a presents the LPWAN deployment context, in which homogeneous end devices communicate directly with gateway infrastructure connected to a server on the Internet. Each evaluated run models one controlled energy-harvesting node, a gateway/channel abstraction, and exogenous background traffic. The node model incorporates a finite-capacity supercapacitor and the declared aggregate power-mode catalogue M0–M19. Node operation follows an explicit service calendar specifying job release times, deadlines, and phase durations. In the reference profile, all service and controller jobs execute in M0; the remaining modes are candidates for low-power residence between jobs. Figure 1b illustrates the node energy-accounting boundary, distinguishing gross harvested energy, node consumption, leakage and other physical losses, and overflow. Node consumption includes sensing, processing, transmission and reception, mode residence, transitions, and controller computation. Gateway energy lies outside this boundary. The storage state evolves over the executed phases according to these realized energy flows, with storage capacity and brownout/restart thresholds specified as model parameters. Figure 1 provides a schematic representation of the numerical model without specifying component-level hardware wiring. The information available to the node and its planning process are detailed in Figure 2.

Figure 2 shows the coordination between gateway-side reporting period control and node-side planning. The gateway generates reporting commands from the context available to it, and the node validates received commands before their application at an eligible nominal boundary. The node then evaluates feasible mode and scheduling plans using its local state and mandatory jobs. Energy demand, service outcomes, and storage behavior are determined by the subsequent physical execution under the declared model.

The feedback path in Figure 2 uses energy-state reports that have actually reached the gateway. Gateway traffic context is derived from received LPWAN activity, and the weather/control-context value is supplied by the scenario input. The gateway therefore acts on available information that can differ from the node’s current physical state. Command generation, transmission, reception, validation, pending status, and application are recorded as distinct stages. Node-side selection remains subject to local service requirements and energy-feasibility constraints.

**Figure 3:**
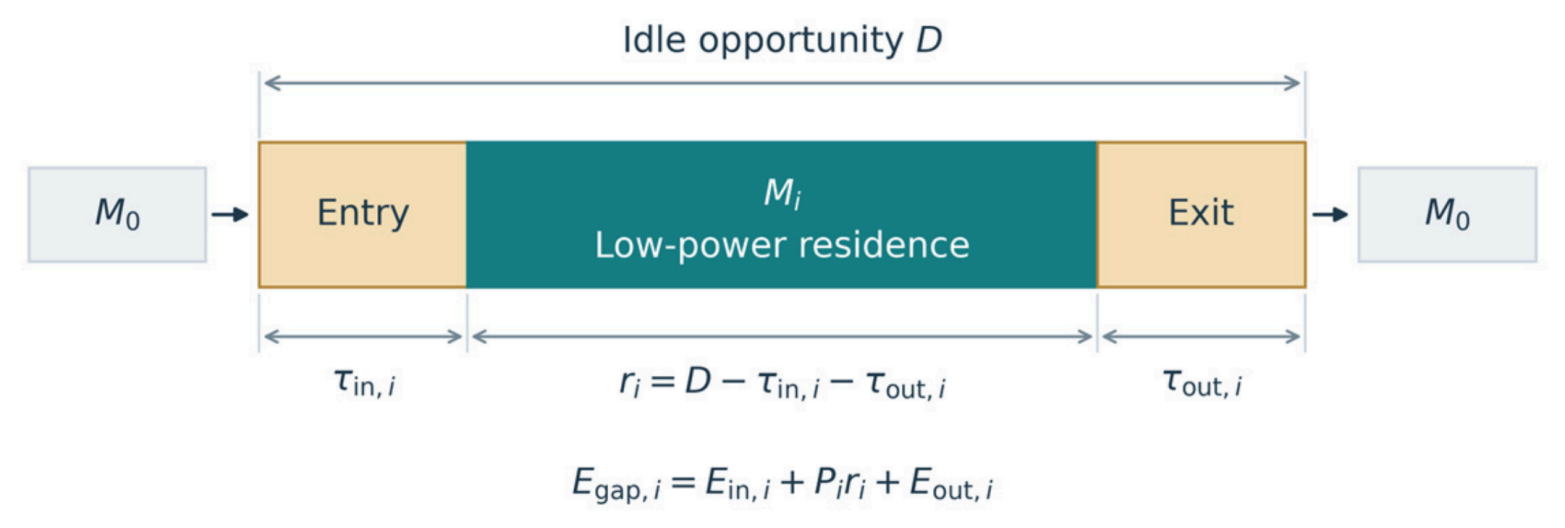
Phase durations and energy accounting within a node idle opportunity.

The source component-state abstraction is summarized in Table 1. For local planning, each candidate combines one of two fixed-order scheduling policies, EARLIEST or LATEST, with one idle mode from M0–M19, yielding a finite catalogue of 40 candidate plans. Within each comparison, all candidates are evaluated using the same local snapshot, mandatory job set, and planning horizon, under equivalent terminal conditions. Feasibility requires compatible capabilities, satisfaction of job precedence and data-readiness constraints, valid timing, admissible mode transitions (Table 2), and sufficient energy reserve throughout the plan. Among feasible candidates, the node controller selects the plan with the lowest estimated total node energy, including mode-transition and controller-computation costs; idle-mode power alone does not determine the selection (Figure 3).

Node energy is estimated over the complete phase sequence of each candidate plan p, including controller computation, mandatory service, mode transitions and idle residence. Let I be the local snapshot at decision time t, and let x be the endpoint of the interval being assessed. The full assessment uses the existing nominal reporting target x=H; a recovery assessment uses an earlier endpoint x=h<H while retaining H. Thus, x−t is the planning duration. The mandatory set $W_{x}$ contains each pending job whose deadline is at or before x, or whose latest feasible start is before x, together with its pending predecessors transitively. Each job is included once, and deferred jobs remain in the service registry.

For a candidate with idle mode $M_{i}$, let $G\left(p\right)$ contain its gaps between or after controller and service phases. Each gap g has total duration $D_{g}$, including entry into and exit from $M_{i}$. All gaps in a candidate use the same declared idle mode. Let $P_{i}$ be its residence power, and let $\tau_{in,i}$ and $\tau_{out,i}$ be the entry and exit durations. The net residence duration is

(1) $$r_{g}=D_{g}-\tau_{in,i}-\tau_{out,i},\;\;r_{g}\geq 0.$$

Durations in the energy expressions are in seconds, power is in watts and energy is in joules. The mode threshold $\theta_{i,ms}$ retains its tabulated numerical value in milliseconds. If $D_{g,ms}=1000D_{g}$ denotes the numerical value of the same gap duration in milliseconds, the threshold condition is

(2) $$D_{g,ms}\geq \theta_{i,ms}.$$

This condition applies to the complete gap, including both transitions. A zero-duration gap contributes no phase or energy. For an admissible positive-duration gap, the node load is

(3) $$E_{gap,i}\left(D_{g}\right)=E_{in,i}+P_{i}r_{g}+E_{out,i}.$$

Here, $E_{in,i}$ and $E_{out,i}$ are the total entry and exit load energies; active-mode baseline energy is already included in these transition costs. Storage losses are accounted for separately. For $M_{0}$, both transition durations and energies are zero. The thresholds are fixed admissibility settings; energy-based selection uses the complete plan estimate.

All controller and service work executes in $M_{0}$, with power $P_{0}$. Let $\tau_{ctl}$ and $\Delta E_{ctl}$ denote the duration and incremental energy of one controller search. For service job j, let $d_{j}$ and $\Delta E_{j}$ denote its duration and incremental energy. The corresponding load energies are

(4) $$E_{ctl}=q\left(P_{0}\tau_{ctl}+\Delta E_{ctl}\right),$$

(5) $$E_{srv}=\sum_{j\in W_{x}}\left(P_{0}d_{j}+\Delta E_{j}\right).$$

The search count is q=1 for the full assessment and q=2 when the recovery catalogue is also assessed. Rejection before that second assessment does not add a second search charge. The aggregate controller interval has duration $q\tau_{ctl}$ and precedes service and idle phases. The controller, service and gap phases partition $\left[t,x\right]$ without overlap. With the configured constant loss estimate $P_{loss,est}$, the total estimated energy of candidate p is

(6) $${\hat{E}}_{tot}\left(p\right)=E_{ctl}+E_{srv}+\sum_{g\in G\left(p\right)}E_{gap,i}\left(D_{g}\right)+P_{loss,est}\left(x-t\right).$$

The loss term covers the complete interval once. At admission, let $E_{available}\left(I\right)$ be the storage-energy observation supplied in the snapshot, before subtracting uncertainty or the reserve floor, and let $u_{E}\left(I\right)$ be its nonnegative uncertainty allowance. If the candidate contains $K\left(p\right)$ ordered phases, reserve feasibility requires

(7) $$E_{available}\left(I\right)-u_{E}\left(I\right)-\sum_{l=1}^{k}\left(E_{load,l}+{\hat{E}}_{loss,l}\right)\geq E_{brownout}+R,k=0,1,\ldots ,K\left(p\right).$$

The empty sum at k=0 checks the initial state. For phase l, $E_{load,l}$ is its load energy and ${\hat{E}}_{loss,l}=P_{loss,est}\Delta t_{l}$, where $\Delta t_{l}$ is its duration. Each estimated phase has a constant nonnegative debit and credits no future harvest, so its minimum estimated storage energy occurs at its end. The prefix checks therefore enforce the reserve floor throughout the estimated plan. The margin R is a constraint above the brownout threshold, not an additional energy charge.

Physical execution uses realized harvesting and physical storage losses. Search costs accrue once during execution, including assessed searches that find no feasible plan, and may be truncated at the observation limit. The configured loss estimate is not a guaranteed upper bound on physical losses under every operating condition. Admission and selection remain conditional on the declared model and catalogue.

The node first evaluates the full planning horizon defined by the existing nominal reporting target. If no feasible plan is found, it may assess a separately recorded causal recovery horizon using the same retained job registry. A recovery plan certifies only its own horizon; the original target and the failed full-horizon assessment remain recorded. If no executable plan is available, the node accrues the cost of searches actually assessed during execution and, if time remains, performs finite M0 or OFF waiting, with actual missed work retained in the service record.

### 3.3. Gateway-Constrained Node Planning

The applied reporting period constrains the node’s nominal reporting calendar. For each decision, the existing nominal target defines the full planning horizon H, and the node derives the mandatory jobs and their pending predecessors from the persistent service registry. Candidate plans are compared against this common workload and horizon. Locally observed events update the node state and pending work; the proposed selector evaluates their service requirements within the declared feasible catalogue.

An operational node validates each received reporting command for its identifier, sequence, finite values, causal timestamps, allowed period range, and age. A valid command is retained as pending; receipt does not move the current nominal target. At an operational nominal boundary, the pending command is revalidated. If none is eligible, the node uses a still-valid cached command or the declared default period. Reusing a cached command does not renew its reception timestamp. The following target is computed from the current nominal target and the applied reporting period. An operational nominal boundary may record a missed TX and is not contingent on successful node transmission or gateway delivery. While OFF, nominal-calendar bookkeeping advances the requested reporting targets using the existing period, without applying a command or the default; this bookkeeping does not represent powered node computation or service.

Algorithm 1 summarizes one decision and execution step for the proposed node selector. SelectFeasible assesses the declared candidates under the same local snapshot, mandatory jobs, horizon, and terminal requirements. Among feasible candidates, selection follows the lexicographic order of estimated total energy, transition count, and plan identifier. Installation revalidates the selected plan before any physical phase is executed. A stale selection returns for replanning; infeasibility, unavailable control, OFF waiting, and incomplete physical execution remain distinct outcomes.
**Algorithm 1.** HA-PM node planning and execution**Inputs:** Node state N, configuration Γ, received events ℰ, observation limit *t*_stop_. **Output:** Decision and actual execution record R; updated node state.
01 procedure NodeControl(N, Γ, ℰ, *t*_stop_)
02  Process received events; settle elapsed nominal targets and actual misses.
03  Restart only at an eligible checkpoint; materialize jobs through the current target H.
04  I ← immutable local snapshot; *W*_H_ ← required jobs and pending ancestors for H.
05  R ← SelectFeasible(I, Γ, *W*_H_, H, 1); retain full-horizon assessments.
06  if R.status = NO_FEASIBLE_PLAN then
07   h ← CausalNearHorizon(I, Γ) using only known jobs and the calendar.
08   if h is defined and t + 2*τ*_ctl_ < h < H then
09   *W*_h_ ← required jobs and pending ancestors for h; retain all other jobs.
10   *R*_h_ ← SelectFeasible(I, Γ, *W*_h_, h, 2); retain the recovery assessment.
11   Record the searches actually attempted; preserve the original target H.
12   if *R*_h_ contains a feasible plan then
13    Attach its plan to R and mark RECOVERY_SELECTED.
14   end if
15   end if
16  end if
17  if R contains a selected plan then
18   Revalidate snapshot, time, calendar and available energy before installation.
19   If stale or the energy check fails, return R with the applicable replan status.
20   Execute the plan under the physical-execution contract, up to *t*_stop_.
21  else
22   Execute only the controller charge for searches actually attempted.
23   If time remains, perform finite *M*_0_ or OFF waiting; record actual missed work.
24  end if
25  return R, retaining actual phases, service outcomes and any censored tail.
26 end procedure
27 function SelectFeasible(I, Γ, W, H, q)
28  Validate input and decision-state preconditions; reject malformed input.
29  Return distinct OFF, energy-observation or control-unavailable status when applicable.
30  C ← {EARLIEST, LATEST} × {*M*_0_, …, *M*_19_}, including the q-search cost.
31  Assess every candidate under the same I, W, H and equivalent terminal conditions.
32  Check timing, capabilities, predecessors, data and mode-transition constraints.
33  Check phase energy and reserve throughout the plan; credit no future harvest.
34  F ← {p ∈ C: p satisfies every feasibility check}.
35  if F = ∅ then
36   return NO_FEASIBLE_PLAN with all candidate assessments.
37  end if
38  p^*^ ← lexicographic arg min over p ∈ F of (*Ê*_tot_(p), *n*_tr_(p), id(p)).
39  return SELECTED, p^*^ and all candidate assessments.
40 end function

For the local snapshot I, configuration Γ, required jobs $W_{x}$, endpoint x and search count q, let $F\left(I,\Gamma ,W_{x},x,q\right)$ denote the feasible subset of the defined catalogue. The catalogue combines EARLIEST or LATEST placement of the fixed job order with one idle mode from $M_{0}$–$M_{19}$, giving 40 candidates before feasibility filtering. Candidates are compared using the same local information, mandatory jobs and endpoint, with terminal mode $M_{0}$, equivalent completed work and the same deferred workload. Feasibility includes timing, capabilities, precedence, data readiness, admissible transitions and the energy-reserve checks defined above.

When F is nonempty, the proposed selector uses the lexicographic minimum

(8) $$p^{*}=\underset{p\in F\left(I,\Gamma ,W_{x},x,q\right)}{{arg\;min}^{lex}}\left({\hat{E}}_{tot}\left(p\right),\;n_{tr}\left(p\right),\;id\left(p\right)\right).$$

Estimated total energy is minimized first. Exact energy ties are resolved by the number of entry and exit phases $n_{tr}\left(p\right)$, followed by the implementation’s plan-identifier ordering. Each explicit entry or exit phase counts once; zero-duration transitions that produce no phase add no count. This rule selects the minimum within the assessed feasible catalogue.

The full assessment uses x=H. If a causal recovery assessment is performed, it uses x=h<H and establishes feasibility only through h; the original nominal target H and the failed full-horizon assessment remain recorded. After the input and control-availability checks have passed, an empty assessed feasible set returns NO_FEASIBLE_PLAN. Earlier guard failures retain their own statuses. This selection rule is used within the search, installation and execution procedure specified by Algorithm 1 and its supporting definitions.

Gateway adaptation and node planning operate at distinct levels. The gateway selects a reporting period from the context available to it; the node determines whether and how its mandatory work can be executed within the resulting calendar. A longer reporting period can change the available low-power opportunities, but actual energy and service outcomes depend on the selected feasible plan and the realized harvesting, storage, and channel conditions. These outcomes are evaluated explicitly in the numerical experiments.

### 3.4. Fuzzy Logic Controller Design

#### 3.4.1. Gateway-Side Control Objective

The gateway FLC proposes a node-to-gateway reporting period from delivered energy-state information, observed network traffic, and current weather/control context. The node validates received commands and incorporates an eligible period into its reporting calendar. Algorithm 1 then selects the feasible power mode and schedule with the lowest estimated total node energy, including transition and controller costs, while satisfying mandatory sensing, processing, transmission (TX) and reception (RX) requirements. The reporting period constrains the calendar; the selected local schedule determines the actual low-power residence.

The nominal reporting boundary is node-side TX completion. Gateway command generation, physical transmission, node reception, validation, and application are distinct events, as are uplink TX completion and successful gateway delivery. This interface permits context-dependent period proposals while retaining node-side timing, service and energy checks. The FLC uses the membership functions, 27 nominal rules and Mamdani operators specified below. Its coordinates, normalization constants and physical control cadence are declared model assumptions for this evaluation. The reporting period defines the reporting cadence; the local feasible schedule determines the actual low-power residence.

#### 3.4.2. Input and Output Variables

The FLC uses three inputs: usable supercapacitor SoC, $S_{SoC}$, gateway-observed network traffic, $N_{load}$, and weather/control-context information, $W_{cond}$. Their linguistic sets are Low/Medium/High, Low/Normal/High, and Poor/Average/Good, respectively. The output sets are Short/Medium/Long reporting periods. The gateway-proposed period $T_{\mathrm{cmd}}$ represents the node-side reporting period $T_{\mathrm{sync}}$; reception and subsequent node validation determine whether it is applied. Throughout this section, t denotes physical simulation time and u denotes a candidate reporting period, both in seconds.

Usable-energy SoC is calculated from node energy E sampled at actual target-report TX start:

(9) $$S_{SoC}=100\mathrm{clip}\left(\frac{E-E_{\mathrm{brownout}}}{E_{\mathrm{capacity}}-E_{\mathrm{brownout}}},0,1\right).$$

Here, $E_{\mathrm{brownout}}$ and $E_{\mathrm{capacity}}$ are the brownout energy and storage capacity, and $\mathrm{clip}\left(x,a,b\right)=\min\left(\max\left(x,a\right),b\right)$. The first successful physical delivery of a report updates the gateway SoC cache while retaining its original TX-start sampling time $\tau_{K}$. The gateway therefore uses delivered telemetry, not direct access to the node’s current energy.

Let $n_{\mathrm{rx}}(\left(t-W,t]\right)$ count successfully received background packets plus the first successful delivery of each target report in window (t−W,t]. With W= 3600 s and reference rate $r_{\mathrm{ref}}\;=\;1/60$ Hz, the traffic input is

(10) $$N_{load}\left(t\right)=\min\left(1,\frac{n_{\mathrm{rx}}(\left(t-W,t]\right)}{Wr_{\mathrm{ref}}}\right)=\min\left(1,\frac{n_{\mathrm{rx}}(\left(t-3600,t]\right)}{60}\right).$$

A target report contributes once even if both physical copies succeed, and a zero count gives N= 0. This delivery-based network signal is distinct from the node’s observed physical-event rate.

The weather/control-context input $W_{cond}\in \left[0,1\right]$ represents harvesting suitability, with larger values indicating more favorable conditions. At each opportunity, it is the current value of the prescribed 300 s trace bin. The evaluated synthetic context trace spans $\left[0.3,\;1\right]$, within the full inference domain. It modulates harvesting and supplies the FLC context; radio transactions use a separate channel model. It provides no credit for future harvested energy. The executed caller derives current traffic and context directly and applies the sampling-age gate to delivered SoC. Its inference call requires finite inputs in their stated domains; missing or nonfinite values are not substituted with zeros.

#### 3.4.3. Membership Functions

**Figure 4:**
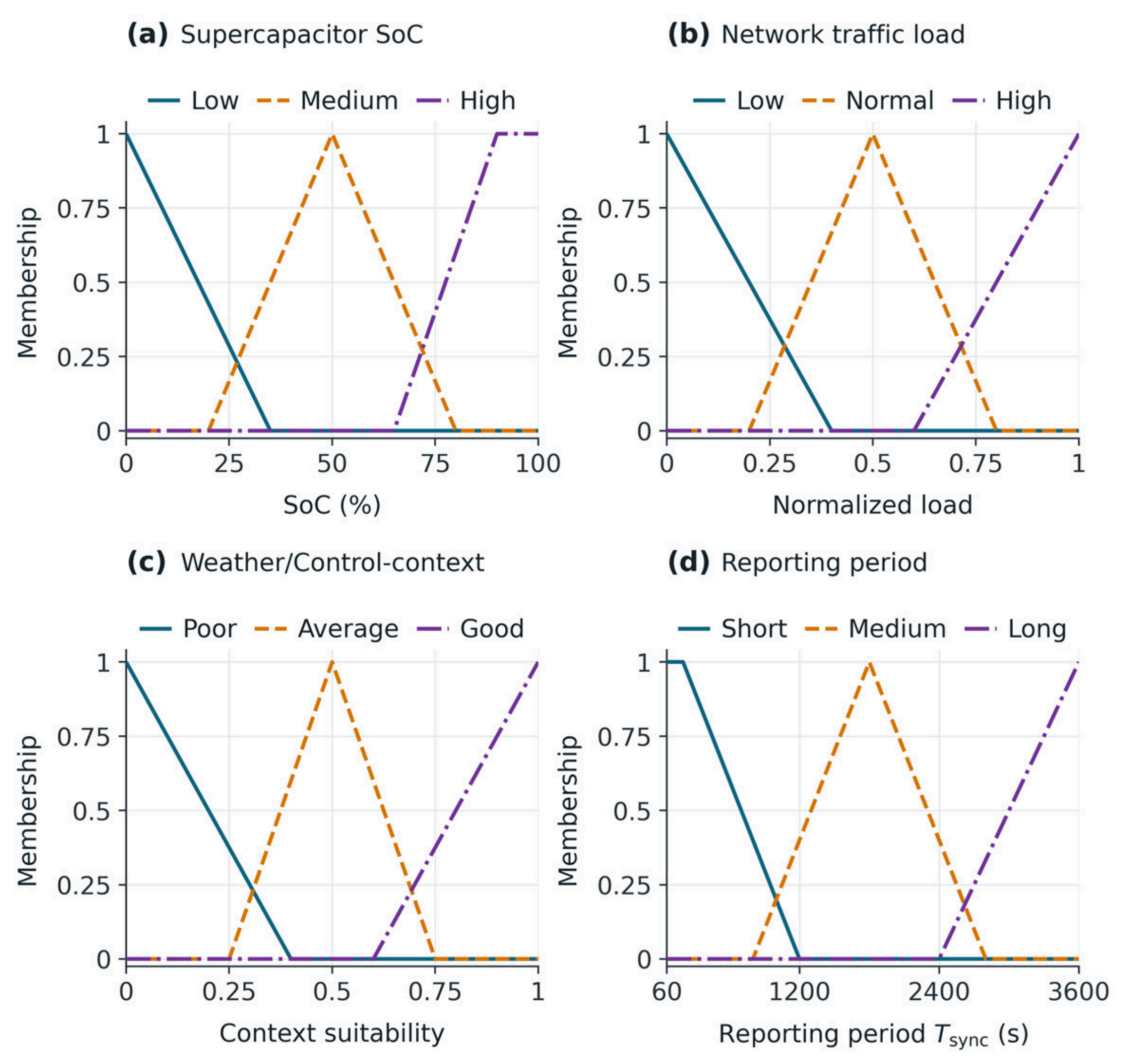
Input and output membership functions of the gateway FLC.

Figure 4 specifies the piecewise-linear membership functions. The input domains are $S_{SoC}\in \left[0,100\right]$% for supercapacitor SoC and $N_{load},W_{cond}\in \left[0,\;1\right]$ for traffic and weather/control context; the output domain is $\Omega =\left[60,\;3600\right]$ s. Overlapping sets and endpoint shoulders cover each full input domain. Consequently, at least one rule has positive activation for every valid input triple, yielding a positive aggregate area and a defined centroid. The knot coordinates are declared parameters of the numerical controller.

The centroid is computed from the continuous aggregated output membership function. The final reporting-period command is quantized downward in 1 ms increments. This distinction separates fuzzy inference from the resolution of the transmitted value.

#### 3.4.4. Rule Base

The complete rule base contains the 27 combinations of the three linguistic sets for each input, as summarized in Table 3. For rule $R_{ijk}\in \{1,\ldots ,27\}$, the antecedent labels $A_{i}$, $B_{j}$, and $C_{k}$ refer to the SoC, network traffic, and weather context sets, respectively, and $D_{ijk}$ is the output.

(11) $$R_{ijk}:\left(A_{i},B_{j},C_{k}\right)\mapsto D_{ijk}.$$

$A_{i}\in \{Low,Medium,High\}$, $B_{j}\in \{Low,Normal,High\}$, $C_{k}\in \{Poor,Average,Good\}$, and $D_{ijk}\in \{Short,Medium,Long\}$. Each rule has unit weight in the full controller.

The nominal rule base and the SoC-dependent output adjustment form two successive stages. Table 3 defines fuzzy inference. After defuzzification and clipping, the Low-SoC membership degree determines how far the reporting period is shifted toward its upper bound. This adjustment is not an additional fuzzy rule.

#### 3.4.5. Inference, Aggregation, and Defuzzification

For a rule $R_{ijk}$, the firing strength is computed using the minimum t-norm for the logical AND operation:

(12) $$\alpha_{ijk}=min\left\{\mu_{A_{i}}\left(S_{SoC}\right),\mu_{B_{j}}\left(N_{load}\right),\mu_{C_{k}}\left(W_{cond}\right)\right\},$$

where $\alpha_{ijk}$ is the firing strength of $R_{ijk}$, and $\mu_{A_{i}}\left(S_{SoC}\right)$, $\mu_{B_{j}}\left(N_{load}\right)$, and $\mu_{C_{k}}\left(W_{cond}\right)$ are the corresponding input membership functions. After rule activation, Mamdani minimum-implication clips the output membership function to obtain each rule’s output contribution. The output fuzzy set generated by rule $R_{ijk}$ is expressed as

(13) $$g_{ijk}\left(u\right)=min\left[\alpha_{ijk},\mu_{D_{ijk}}\left(u\right)\right],$$

where $\mu_{D_{ijk}}\left(t\right)$ is the output membership function associated with the consequent linguistic set $D_{ijk}$, and $u\epsilon \Omega_{t}=\left[T_{min},T_{max}\right]$ denotes a candidate reporting period value in the output universe of discourse.

The outputs of all evaluated rules are then combined by bounded-sum aggregation.

(14) $$g\left(u\right)=\min\left\{1,\sum_{ijk=1}^{27}g_{ijk}\left(u\right)\right\}.$$

Thus, rules supporting the same consequent can reinforce one another, and conflicting recommendations remain simultaneously active. There are no OR antecedents or winning-rule selection. The continuous aggregate $g\left(u\right)$ is converted to the preliminary reporting period by centroid defuzzification:

(15) $$T_{\mathrm{raw}}=\frac{\int_{60}^{3600}u\;g\left(u\right)\;du}{\int_{60}^{3600}g\left(u\right)\;du}.$$

The denominator is the aggregate area A in seconds, and the numerator is its first moment M in seconds squared. The implementation inserts active-consequent membership knots, implication-clipping intersections and bounded-sum saturation crossings, then integrates each resulting affine segment analytically using binary64 arithmetic. It uses no sampled output grid, and its arithmetic introduces no additional physical control-loop cadence.

Nonfinite or out-of-domain inputs, a nonpositive or nonfinite area, or a nonfinite moment raise an explicit FLCError. The executed gateway caller invokes inference directly and does not convert these errors into a silent no-command fallback. This evaluation-error behavior is distinct from ordinary opportunity ineligibility due to outage or unavailable/stale SoC.

#### 3.4.6. Reporting-Period Adjustment, Dissemination, and Fault Tolerance

The continuous centroid is first bounded, then adjusted using the SoC Low membership, and finally quantized. In the evaluated profile, $T_{\min\;}=\;60$ s, $T_{\mathrm{cap}}\;=\;T_{\max}\;=\;3600$ s and q=0.001 s:

(16a) $$T_{0}=\mathrm{clip}\left(T_{\mathrm{raw}},T_{\min},T_{\mathrm{cap}}\right),$$

(16b) $$T_{\mathrm{env}}=T_{0}+\mu_{\mathrm{Low}}\left(S\right)\left(T_{\mathrm{cap}}-T_{0}\right),$$

(16c) $$T_{\mathrm{cmd}}=Q\left(T_{\mathrm{env}}\right),\;Q\left(x\right)=\mathrm{clip}\left(q\left\lfloor \frac{x}{q}\right\rfloor ,T_{\min},T_{\mathrm{cap}}\right).$$

Here, $T_{0}$ is the bounded pre-adjustment period and $T_{\mathrm{env}}$ is the unquantized adjusted period. The library also permits a finite service cap, in which case $T_{\mathrm{cap}}\;=\;\min\left(T_{\max},T_{\mathrm{service},\max}\right)$; the evaluated profile supplies no additional cap. A nonfinite cap or an empty admissible interval is rejected. A service cap changes the post-centroid bound, not the integration universe Ω.

For the same valid input and fixed bounds, the real-arithmetic construction gives $T_{0}\leq T_{\mathrm{env}}\leq T_{\mathrm{cap}}$ and, using the same monotone quantizer on both outputs, $Q\left(T_{0}\right)\leq T_{\mathrm{cmd}}\leq T_{\mathrm{cap}}$. These inequalities compare adjusted and unadjusted outputs at the same input and fixed bounds; the full mapping is not generally monotone in each input. Interval bounding defines admissible reporting commands; local service and energy feasibility remain node-side decisions. The source applies floor-and-clamp operations to binary64 values; values near a quantization boundary may differ by one nominal quantum from exact-real arithmetic.

Algorithm 2 describes the executed full-input controller for one target node. Gateway opportunities occur at t, aligning with the start of each scheduled 0.1 s node RX window. Gateway outage, a missing delivered SoC cache, or a SoC sampling age greater than 7200 s suppresses command creation. Current traffic and trace context are obtained at an eligible opportunity. Every valid evaluation creates one newly sequenced command, even when its proposed period is unchanged.

Two predetermined 0.04 s copies of the same command are offered at t and t+ 0.05 s, irrespective of first-copy success. Their completion times are t+0.04 s and t+ 0.09 s. Channel-successful copies reach node validation only after the matching 0.1 s RX service completes in full, while retaining their physical copy-completion reception times. A generated or transmitted command is therefore not automatically a received or applied period.

On valid reception, the node retains its current nominal TX-completion target and holds the command pending. At an operational nominal boundary, it revalidates the pending command; otherwise, it reuses an unexpired active command, or uses the 900 s default. Command age is limited to 1800 s from original reception, and duplicate copies or reuse do not refresh that age. During OFF, no command or default period is applied; after restart, eligibility is checked again at an operational nominal boundary. Node-side calendar and admission checks continue to determine service and energy feasibility. Gateway inference therefore changes the proposed reporting cadence without replacing local schedule selection.
**Algorithm 2.** Gateway inference and reporting-period command generation**Scope and output:** Full-input controller for one target node. Each valid opportunity produces a sequenced period command $T_{\mathrm{cmd}}$, the proposed $T_{\mathrm{sync}}$.
**Inputs and state:** Delivered SoC telemetry and sampling times; completed uplink deliveries; current trace context; the defined memberships and 27-rule table. Cache $K=\left(S_{K},\tau_{K}\right)$ stores usable-energy SoC (%) and its sampling time; s is the sequence.
**Profile:** $t\in T_{g}=\{\left(300m-1\right)\;s:m=1,2,\ldots \}$; $T_{\mathrm{age}}=7200\;s$;
W=3600 s, $r_{\mathrm{ref}}=1/60\;\mathrm{Hz}$; $T_{\min}=60\;s$, $T_{\mathrm{cap}}=3600\;s$, q=0.001 s;
Ω=[60,3600] s; S∈[0,100], normalized traffic N∈[0,1], context C∈[0,1]. *Delivered telemetry*
1 Initialize K←missing and s←0
2 On the first successful physical delivery of a target report:
3  K←(reported SoC, telemetry sampling time)
4  Record this report’s first successful delivery time
*Scheduled inference*
5 At each opportunity $t\in T_{g}$:
6  Commit completed physical uplink observations through t
7  If the gateway is available and K is present with $t-\tau_{K}\leq T_{\mathrm{age}}$:
8   $S\leftarrow S_{K}$; $C\leftarrow \mathrm{CurrentContext}\left(t\right)$
9   $N\leftarrow \min\;\left(1,\mathrm{DeliveredCount}(\left(t-W,t]\right)/\left(Wr_{\mathrm{ref}}\right)\right)$
10   Compute membership degrees for S, N, and C
11   For each rule r in the 27-rule table:
12   $\alpha_{r}\leftarrow$ minimum of its three antecedent membership degrees
13   $g_{r}\left(u\right)\leftarrow \min\;\left(\alpha_{r},\mu_{D_{r}}\left(u\right)\right)$, for consequent $D_{r}$
14   End for
15   $g\left(u\right)\leftarrow \min\;\left(1,\sum_{r=1}^{27}g_{r}\left(u\right)\right)$
16   $T_{\mathrm{raw}}\leftarrow \frac{\int_{\Omega }u\;g\left(u\right)\;du}{\int_{\Omega }g\left(u\right)\;du}$ (analytic continuous centroid)
*Period construction and dissemination*

17   $T_{0}\leftarrow \mathrm{clip}\left(T_{\mathrm{raw}},T_{\min},T_{\mathrm{cap}}\right)$
18   $T_{\mathrm{env}}\leftarrow T_{0}+\mu_{\mathrm{Low}}\left(S\right)\left(T_{\mathrm{cap}}-T_{0}\right)$
19   $T_{\mathrm{cmd}}\leftarrow \mathrm{clip}\;\left(q\lfloor T_{\mathrm{env}}/q\rfloor ,T_{\min},T_{\mathrm{cap}}\right)$
20   s←s+1; create command (identifier, s, generation time t, $T_{\mathrm{cmd}}$)
21   Offer two 0.04 s blind copies of this command at t and t+0.05 s
22  Else: generate no command at this opportunity
23  End if
24 End
**Data contract:** Telemetry is sampled at actual target TX start. DeliveredCount includes successful background deliveries and the first successful delivery of each target report. CurrentContext returns the current prescribed trace value.
**Evaluation contract:** Inputs are finite and in the stated domains; the centroid has positive finite area and finite first moment. An invalid evaluation raises FLCError. The Low-SoC envelope follows the centroid and clipping; quantization floors to 1 ms.
**Node interface:** After the matching RX service completes, channel-successful copies reach Algorithm 1 for validation and subsequent application at an operational nominal boundary.

### 3.5. Simulation

The HA-PM framework was evaluated using a custom, event-driven LPWAN reference simulator with controlled energy-harvesting nodes, a gateway, and exogenous background uplink traffic. The simulator couples physical event arrivals, mandatory sensing and processing, data queues, radio transactions, gateway commands, node power-state transitions, and supercapacitor energy dynamics. Node-side total-energy selection is limited to the declared catalogue of 40 candidates, comprising 20 aggregate power modes and 2 fixed-order schedules. The reporting period $T_{sync}$ denotes the nominal reporting period whose boundary is node TX completion; sensing and receiver-listening obligations follow their own service calendars.

The completed campaign contains 432 simulations of 72 h each. At both the 1× and 8× buffer profiles, eight matched exogenous seeds were used for six scenarios: nominal, mixed traffic, gateway outage, elevated temperature, elevated leakage, and no harvesting. Four internal arms combine either a fixed period or the full FLC with either the lowest-residence-power feasible EARLIEST selector or minimum estimated total-energy selection. Three structural input-removal variants of the full framework were additionally evaluated under mixed traffic. Within each seed and scenario, the arms share external event, weather, harvest, temperature, and keyed random-channel inputs, while energy, queues, delivered information, and gateway knowledge evolve independently. The 8× profile scales capacitance, stored-energy thresholds and reference leakage together and is a declared model profile, not measured hardware.

Physical events and offered background packets follow separate temporal arrival processes. Events remain detectable and are observed only when sensing physically completes; finite queue and packet capacities then determine reporting opportunities. Background packets affect collision losses and the gateway traffic input, which counts successfully received background and studied-node uplinks during the preceding hour. A synthetic weather index modulates the diurnal harvest profile and supplies $W_{cond}$ to the FLC. Radio loss is modeled separately through interval overlaps, gateway availability, and time-keyed random losses; this model does not include weather-dependent propagation or spatial coverage.

Energy and service are evaluated over the fixed 72 h observation window. Completed, missed and pending jobs and undelivered queue contents are retained separately. Deadline-based service success requires every job due by the horizon to complete by its own physical deadline; a run without a first brownout is right-censored at 72 h. Actual node load energy is reported alongside node draw, including leakage and other losses, and the separately stated node-plus-gateway total; background-device energy is outside that system boundary. Energy differences are interpreted together with offered and completed service, because full-loop arms need not perform equal work. These results characterize the declared simulation and do not establish hardware performance, lifetime beyond the observation window, or guaranteed non-depletion.

The three structural variants remove one FLC antecedent and average the consequent weights over its three linguistic categories. Removing SoC also disables the Low-SoC output adjustment. However, the runner retains its common delivered-SoC presence and age gate in all adaptive variants. The SoC removal comparison therefore tests removal of SoC from the inference and adjustment, rather than removal of every SoC dependency from the protocol.

Table 4 summarizes the simulation settings. The mode catalog and energy accounting are described in the methodology section. Additional results are provided in the Supplementary Materials.

## 4. Results and Analysis

### 4.1. FLC Decision-Surface Behavior

The first set of results examines the decision-surface behavior of the gateway-side fuzzy logic controller. The objective of this analysis is to assess whether the FLC produces a reporting period that is consistent with the rule-base design. The decision surfaces visualize the mapping from the input context variables ($S_{SoC}$, $N_{load}$, and $W_{cond}$) to the adaptive reporting period ($T_{sync}$).

**Figure 5:**
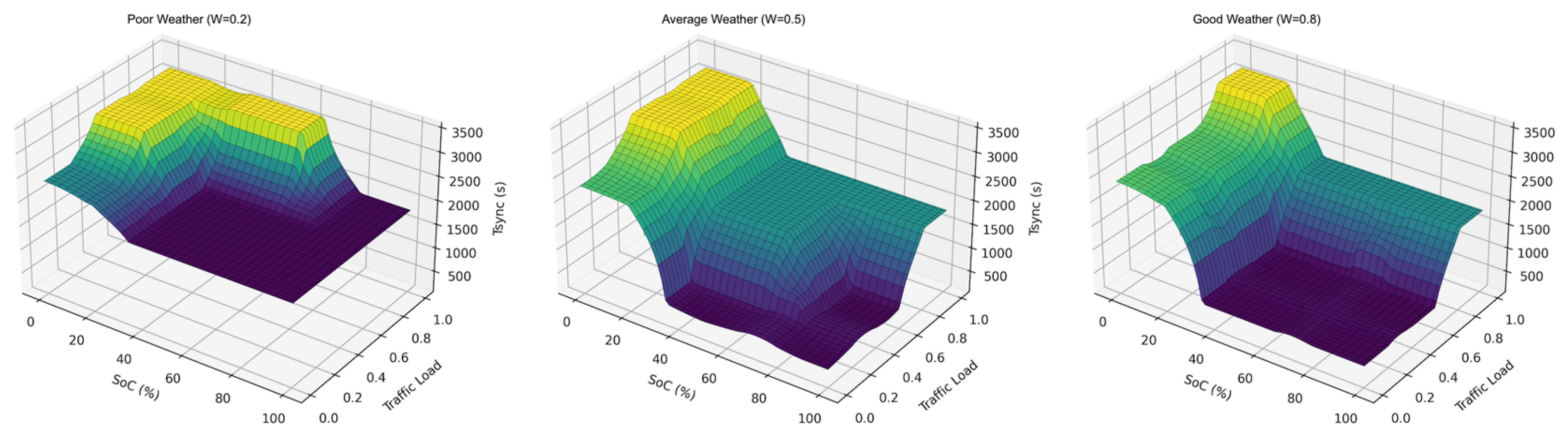
FLC decision surfaces ($T_{sync}$ as a function of $S_{SoC}$, $N_{load}$, and $W_{cond}$).

As shown in Figure 5, the FLC output varies gradually across the input space because of the overlapping membership functions and centroid-based defuzzification. This behavior is desirable for gateway-side synchronization control because small variations in the input context are translated into moderated changes in $T_{sync}$. Instead of relying on abrupt threshold-based switching, the controller adjusts the reporting period according to the combined energy, traffic, and environmental conditions.

The decision surfaces show three main operating regions. First, when $S_{SoC}$ is low, the controller shifts $T_{sync}$ toward longer reporting periods. This behavior is consistent with the Low-SoC safety-priority logic, which biases the aggregated fuzzy output toward energy-conservative synchronization decisions. In this region, longer reporting periods reduce the expected frequency of wake-up and radio activity, thereby supporting increased low-power residency at the node side. Second, when $N_{load}$ is high, the controller tends to increase $T_{sync}$ toward medium or long periods. This behavior reflects the congestion-aware component of the rule base. Under a high traffic load, reducing the frequency of synchronization updates can decrease control-plane pressure and moderate additional radio activity associated with frequent synchronization. Therefore, the FLC output is consistent with the intended network traffic aware adaptation mechanism. Third, shorter reporting periods are primarily observed under favorable operating conditions, particularly when energy availability is high, traffic load is low, and weather conditions are favorable. This region represents opportunistic responsiveness: when the node has sufficient energy and the network is not heavily loaded, the controller allows more frequent synchronization to improve responsiveness. These shorter periods remain constrained by the system-level bounds.

Across the fixed-weather decision surfaces in Figure 5, the controller preserves the same general trend while adapting the output according to the weather condition. Less favorable weather shifts the controller toward more conservative synchronization behavior, whereas favorable weather permits shorter reporting periods when the energy and traffic conditions also support them. This behavior is consistent with the role of $W_{cond}$ as an external context variable affecting the synchronization decision.

The decision-surface behavior indicates that the FLC produces interpretable synchronization decisions aligned with the three design principles of the rule base: energy preservation under low-SoC conditions, traffic-aware moderation under high-load conditions, and responsiveness under favorable energy and network states. These results support the use of the gateway-side FLC as a transparent context-inference mechanism for adaptive synchronization in the proposed HA-PM framework (see SM).

### 4.2. Energy Consumption and Mode Residency

We evaluate the gross daily device energy consumption and node-side mode-residency behavior of the proposed HA-PM framework. The analysis uses the system-level power-mode abstraction defined in Table 1 and the corresponding power-mode parameters reported in Table 2. In particular, the mode power $P_{i}$, transition latency $t_{i}$, and minimum idle-time threshold $T_{i}$ define how the EH-DPM policy selects among the available operating modes $M_{0}$–$M_{19}$.

The fixed synchronization baselines are defined using constant reporting periods of 5 min, 10 min, 15 min, 30 min, and 1 h. These baselines are compared with five HA-PM operating scenarios generated by the gateway-side FLC: critical survival, low-energy favorable, typical balanced, congested energy-rich, and opportunistic ideal. For each HA-PM scenario, the reporting period $T_{sync}$ is not manually assigned; it is computed by the FLC from the corresponding $S_{SoC}$, $N_{load}$, and $W_{cond}$ values.

For a given synchronization policy, the node is assumed to enter the appropriate active mode whenever an event occurs and then transition to the deepest feasible low-power mode during the inactive interval. The feasible idle mode is selected according to the threshold values in Table 2. Specifically, a candidate low-power mode is selected only when the inactive interval is long enough to satisfy its corresponding $T_{th,i}$. This mechanism allows deeper modes to be used only when the synchronization schedule provides sufficient time to amortize the corresponding transition overhead. In this formulation, the energy calculation is based on the accumulated residency time in each mode, while transition feasibility is represented through the idle-time threshold logic.

**Figure 6:**
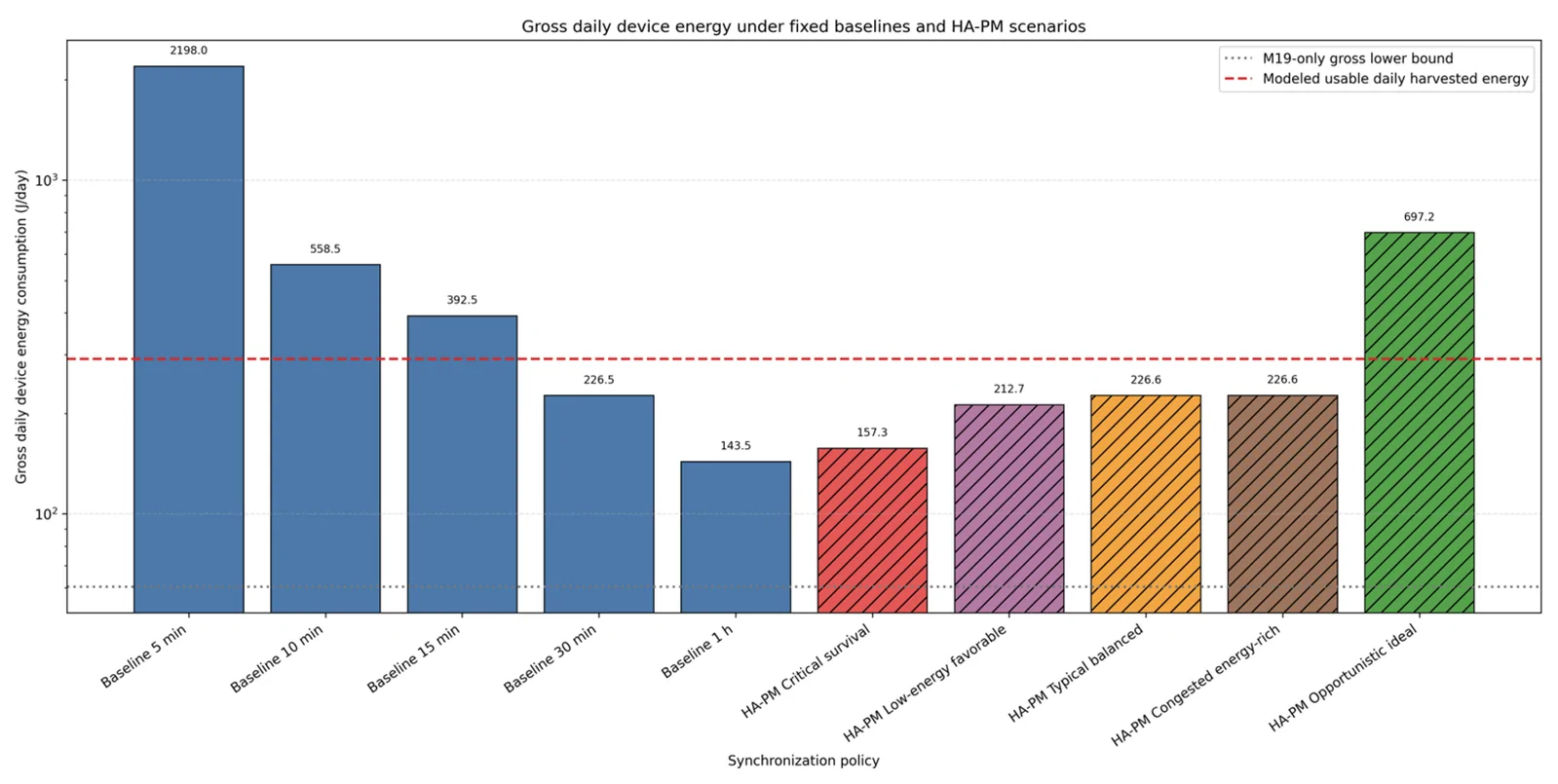
Gross daily device energy consumption.

Figure 6 shows the gross daily device energy consumption under the fixed synchronization baselines and the FLC-derived HA-PM scenarios. The $M_{19}$-only lower bound is also shown as a physical consistency reference. Since $M_{19}$ consumes 0.7 mW, a device that remains continuously in $M_{19}$ for one day still consumes approximately 60.48 J/day.

Shorter fixed reporting periods lead to higher gross daily energy consumption because they increase the number of wake-up and synchronization events. As the fixed reporting period becomes longer, the node spends a larger fraction of the day in lower-power modes, and the gross daily energy consumption decreases. The HA-PM scenarios follow the same physical principle, but their reporting periods are derived from the operating context rather than fixed a priori. Consequently, HA-PM can produce more conservative synchronization behavior under low-energy or congested conditions while allowing shorter reporting periods under favorable conditions.

**Figure 7:**
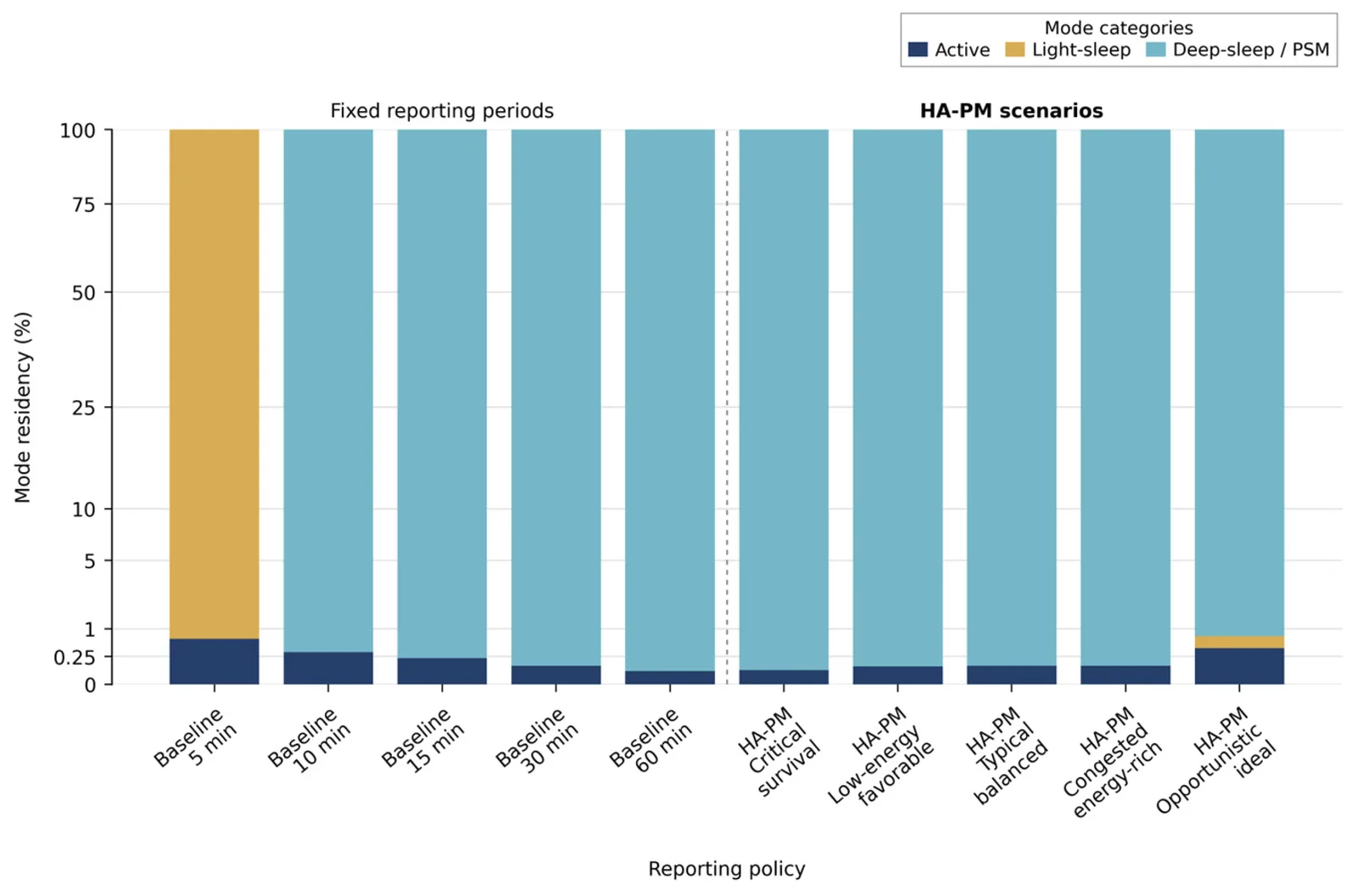
Grouped node-side mode-residency distribution.

Mode residency is evaluated to explain how the energy differences arise from EH-DPM behavior. For each operating mode $M_{i}$, the residency ratio is computed as

(17) $$R_{i}=\left(\frac{T_{i}}{\sum_{0}^{19}T_{j}}\right)\times 100\%,\;$$

where $T_{i}$ is the accumulated time spent in mode $M_{i}$ over the daily evaluation horizon. For a multi-node simulation, $T_{i}$ can equivalently be interpreted as the aggregate residency time of mode $M_{i}$ across all evaluated nodes. Figure 7 groups the detailed modes into functional categories, including active, communication-active, clock-gated, sleep, light-sleep, and deep-sleep/PSM states, according to Table 1.

The mode-residency results show that longer reporting periods increase the opportunity for deeper low-power residency. When the inactive interval exceeds the thresholds of the deeper modes, the EH-DPM policy can select $M_{18}$ or $M_{19}$ rather than remaining in higher-power modes. Conversely, shorter reporting periods limit the feasible idle-mode depth because the inactive period may not be long enough to satisfy the corresponding threshold. This behavior is consistent with the threshold sequence in Table 2, where $M_{15}$, $M_{16}$, $M_{17}$, $M_{18}$, and $M_{19}$ require minimum idle-time thresholds of 7 ms, 15 ms, 30 ms, 189 ms, and 300 ms, respectively.

The results indicate that the proposed HA-PM framework reduces gross daily device energy consumption under the evaluated scenarios by coupling gateway-side context-aware synchronization with node-side EH-DPM mode selection. Unlike fixed periodic synchronization, HA-PM adapts $T_{sync}$ according to the joint effect of supercapacitor SoC, network traffic load, and weather condition. This allows the node to increase low-power residency under energy-constrained or congested conditions while preserving more responsive operation when the operating context is favorable.

### 4.3. Supercapacitor SoC Evolution and Operational Lifetime

We evaluate the storage-level behavior of the proposed HA-PM framework by analyzing the evolution of the supercapacitor voltage and the resulting operational lifetime of the device. The objective is to determine whether HA-PM-driven synchronization adaptation can slow storage depletion under explicitly specified harvesting, leakage, workload, and power-mode assumptions. The operational lifetime is not imposed as a target value; instead, it is computed from the simulated voltage trajectory as the first time at which the supercapacitor reaches the minimum operational voltage threshold.

In the dynamic HA-PM lifetime simulation, the supercapacitor SoC is not treated as a fixed scenario input. Instead, the usable SoC is recomputed from the current stored energy at each simulation step:

(18) $$S_{SoC}\left(t\right)=100\cdot \frac{E_{SC}\left(t\right)-E_{min}}{E_{max}-E_{min}},$$

where $S_{SoC}\left(t\right)$ is clipped to the interval [0, 100]. This feedback formulation allows the HA-PM controller to react to progressive storage depletion. At each control step, the current supercapacitor SoC, time-varying network traffic condition, and weather/harvesting-context condition are passed to the gateway-side FLC:

(19) $$\left(S_{SoC}\left(t\right),N_{load}\left(t\right),\;W_{cond}\left(t\right)\right)\to T_{sync}\left(t\right).$$

The resulting reporting period $T_{sync}\left(t\right)$ is quantized to an implementable gateway command resolution and then passed to the EH-DPM energy model. The EH-DPM policy determines the active and inactive residency times and selects the deepest feasible low-power mode according to the threshold constraints defined in Table 2. The gross daily device energy consumption at time t is computed from the resulting mode-residency distribution as

(20) $$E_{cons}\left(t\right)=\sum_{i}\left(\frac{P_{i}}{1000}\right)T_{i}\left(t\right),$$

where $P_{i}$ is the gross device power of mode $M_{i}$ in milliwatts, and $T_{i}\left(t\right)$ is the accumulated daily residency time of mode $M_{i}$ in seconds. In the evaluated configuration, the deepest sleep mode $M_{19}$ has a nonzero power consumption of 0.7 mW, corresponding to 60.48 J/day.

The supercapacitor energy is updated using a discrete-time energy-balance model. For a simulation interval of length Δt, expressed in days, the stored energy is updated as

(21) $$E_{SC}\left(t+\Delta t\right)=min\left[E_{max},max\left(0,E_{SC}\left(t\right)+\left(E_{harv}\left(t\right)-E_{cons}\left(t\right)\right)\Delta t-E_{leak}\left(t,\Delta t\right)\right)\right],$$

where $E_{harv}\left(t\right)$ is the harvested energy per day, $E_{cons}\left(t\right)$ is the gross daily device energy consumption, and $E_{leak}\left(t,\Delta t\right)$ is the supercapacitor leakage loss over the interval. When leakage is included, the leakage energy is modeled as

(22) $$E_{leak}\left(t,\Delta t\right)=I_{leak}V_{SC}\left(t\right)86400\Delta t,$$

where $I_{leak}$ is the leakage current. Because supercapacitor leakage depends on the selected component, voltage, temperature, aging, and hardware implementation, $I_{leak}$ is treated as an explicit simulation parameter rather than a universal constant.

After each energy update, the corresponding supercapacitor voltage is obtained as

(23) $$V_{SC}\left(t\right)=\sqrt{\frac{2E_{SC}\left(t\right)}{C}}.$$

The operational lifetime is defined as the first time at which the simulated voltage reaches the minimum operational threshold:

(24) $$L_{op}=min\left\{t:V_{SC}\left(t\right)\leq V_{min}\right\}.$$

If $V_{SC}\left(t\right)$ does not reach $V_{min}$ within the simulated horizon, the result is reported as right-censored at the horizon rather than as an infinite or guaranteed lifetime. For example, for a 10-year simulation horizon, this is expressed as $L_{op}\geq 10$ years. This notation means only that the threshold was not reached within the simulated horizon under the specified assumptions; it does not constitute a hardware lifetime guarantee.

**Figure 8:**
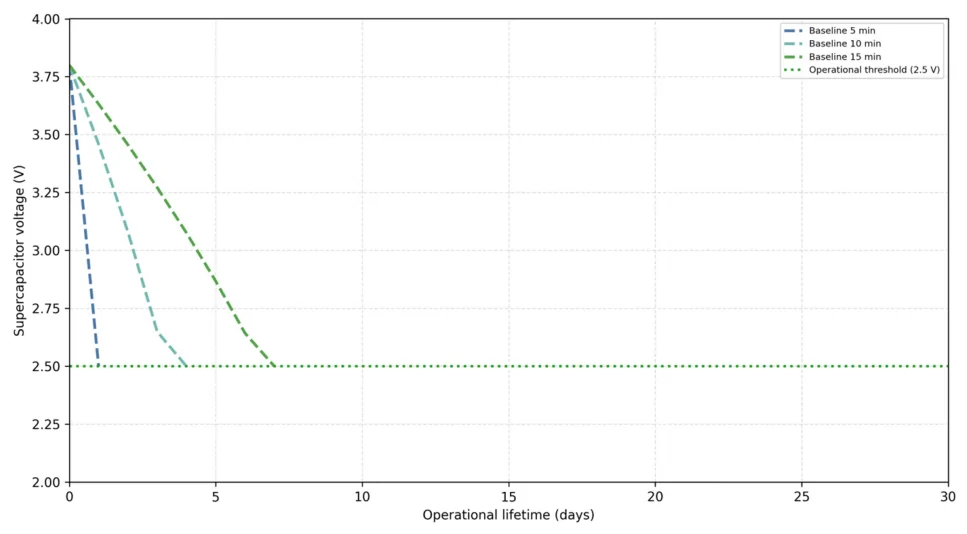
Short-horizon voltage projection for rapidly depleted fixed baselines.

Figure 8 presents a short-horizon voltage projection for the rapidly depleted fixed synchronization baselines. This zoomed view is included because the 5, 10, and 15 min reporting periods reach the minimum operational threshold within days and would otherwise be visually compressed on the multi-year lifetime axis. The result shows that frequent synchronization substantially accelerates supercapacitor depletion under the evaluated harvesting, leakage, workload, and power-mode assumptions.

**Figure 9:**
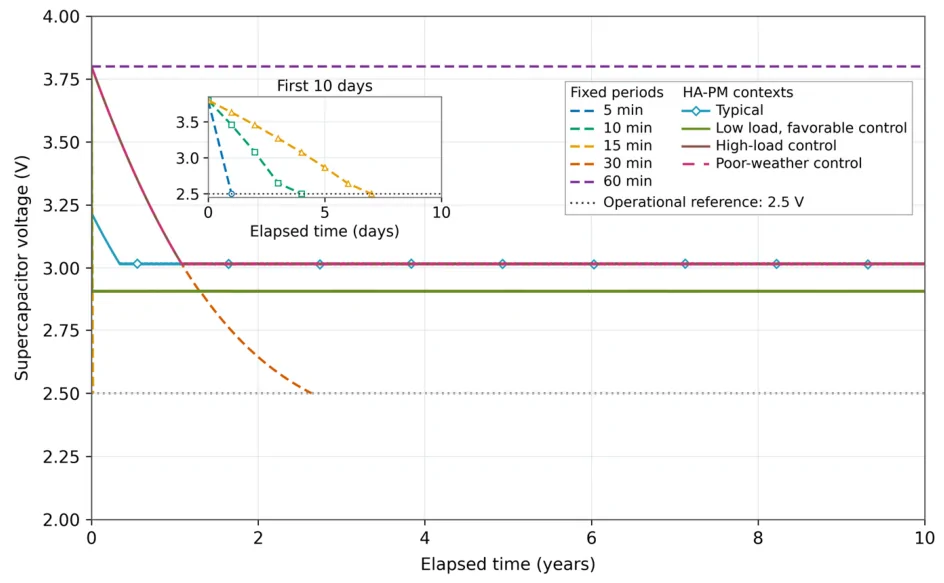
Voltage projection for all fixed baselines and dynamic HA-PM contexts.

Figure 9 presents the supercapacitor voltage trajectories for all fixed synchronization baselines and dynamic HA-PM contexts. The results show that operational continuity is strongly affected by synchronization frequency and control context. Under the evaluated harvesting, leakage, workload, and power-mode assumptions, HA-PM maintains the voltage above the minimum operational threshold throughout the simulated horizon for the evaluated contexts, whereas frequent fixed synchronization baselines deplete much earlier.

**Figure 10:**
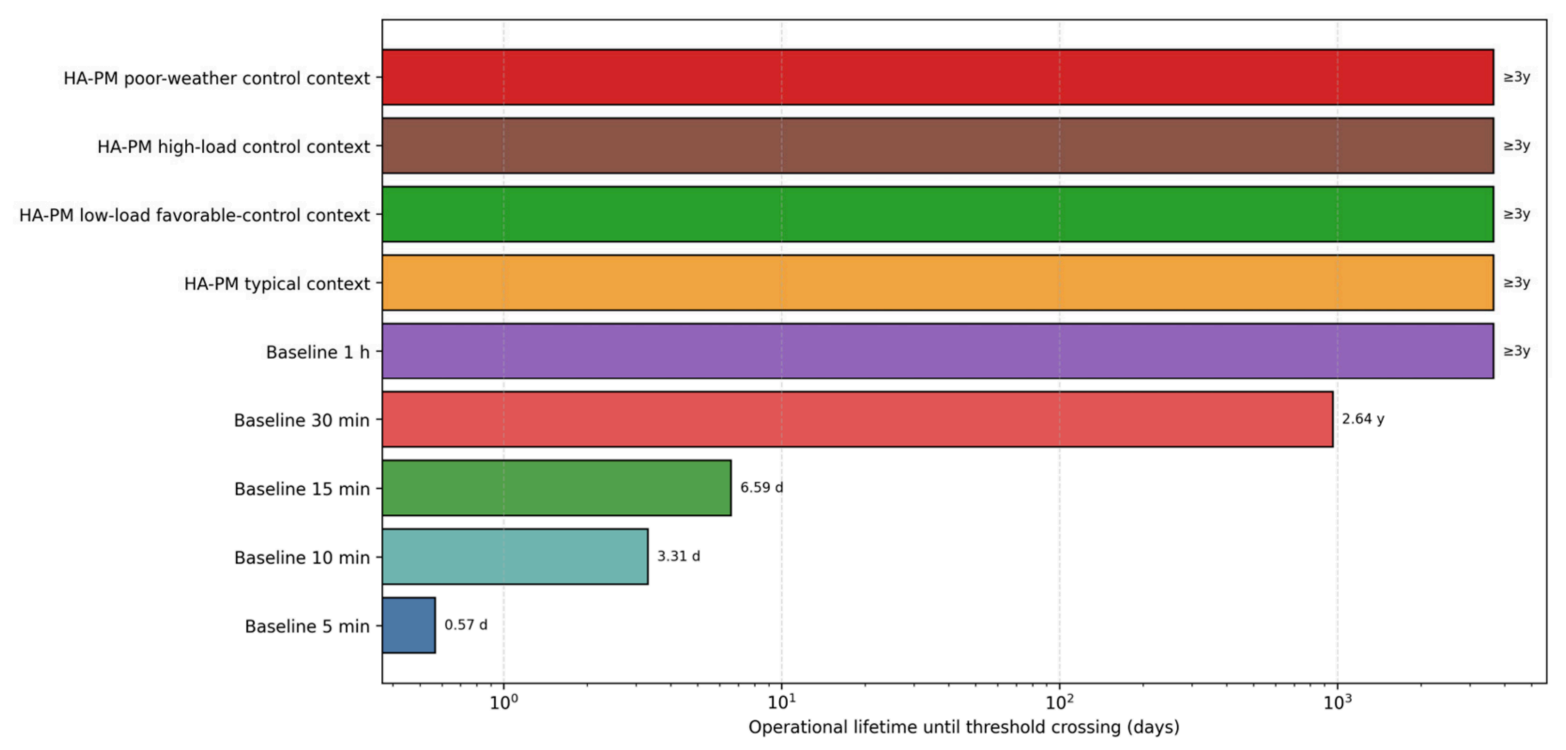
Operational lifetime projection under identical harvesting assumptions.

Figure 10 summarizes the computed operational lifetimes using a logarithmic day-scale representation. The logarithmic scale is appropriate because the evaluated policies span a wide range of threshold-crossing times, from less than one day to right-censored multi-year results.

Under the evaluated assumptions, the HA-PM contexts converge toward a stable medium synchronization region. This indicates that the controller does not operate as a purely aggressive lifetime-maximization policy; rather, it stabilizes around reporting periods determined by the combined effect of supercapacitor SoC, network traffic load, weather/control context, and the Low-SoC safety-priority rule.

**Figure 11:**
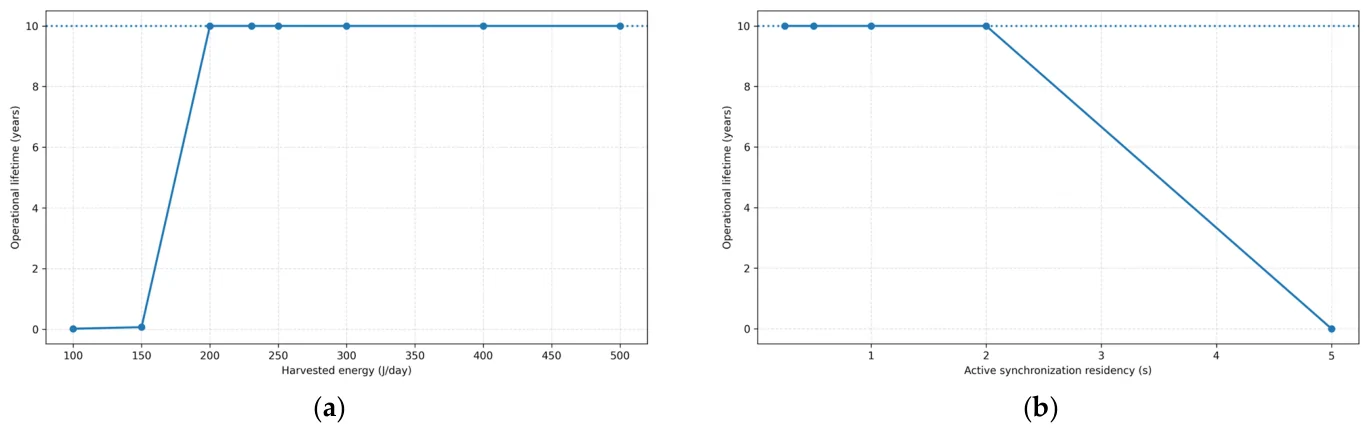
Sensitivity analysis of HA-PM on operational lifetime. (**a**) Sensitivity of dynamic HA-PM operational lifetime to harvested energy. (**b**) Sensitivity of dynamic HA-PM operational lifetime to active synchronization residency.

Figure 11 presents sensitivity analyses for key physical and workload assumptions. These analyses show that the reported lifetime depends strongly on assumed harvesting, leakage current, and synchronization-workload conditions. Therefore, the reported operational lifetime should always be interpreted together with the corresponding assumptions and sensitivity results.

The storage-level analysis indicates that the proposed HA-PM framework improves operational lifetime relative to frequent fixed synchronization baselines under the evaluated deterministic assumptions. The improvement is achieved by adapting $T_{sync}\left(t\right)$, which changes the node’s mode-residency distribution and gross daily energy consumption. However, HA-PM should not be interpreted as a universal lifetime guarantee or as the most energy-conservative possible policy. The results support the conclusion that HA-PM provides a context-aware trade-off between energy preservation and synchronization responsiveness, with operational lifetime remaining conditional on the specified harvesting, leakage, workload, and device-power assumptions (see SM).

### 4.4. Stress-Test Performance Under Adverse Conditions

We evaluate the behavior of the proposed HA-PM framework under adverse environmental and network traffic conditions. The analysis focuses on short-term operational resilience during abrupt stress events. The objective is to determine whether HA-PM can adapt its reporting period and preserve the supercapacitor voltage above the minimum operational threshold under combined harvesting degradation and network traffic variation.

A multi-phase stress scenario was constructed to represent rapidly changing operating conditions. The scenario includes favorable operation, increased network congestion, severe weather-induced harvesting reduction, post-stress recovery, and intermediate mixed conditions. The stress inputs are represented by the weather/control-context variable $W_{cond}\left(t\right)$ and the network traffic load variable $N_{load}\left(t\right)$. These variables are supplied to the gateway-side FLC together with the current supercapacitor SoC, as formulated in Equation (13).

The resulting reporting period $T_{sync}\left(t\right)$ is then used by the EH-DPM model to determine active and low-power residency. Under adverse conditions, HA-PM increases $T_{sync}\left(t\right)$ to reduce the frequency of communication events and increase low-power-mode residency. Under more favorable conditions, the controller can reduce $T_{sync}\left(t\right)$ to improve responsiveness while remaining within the available energy budget.

**Figure 12:**
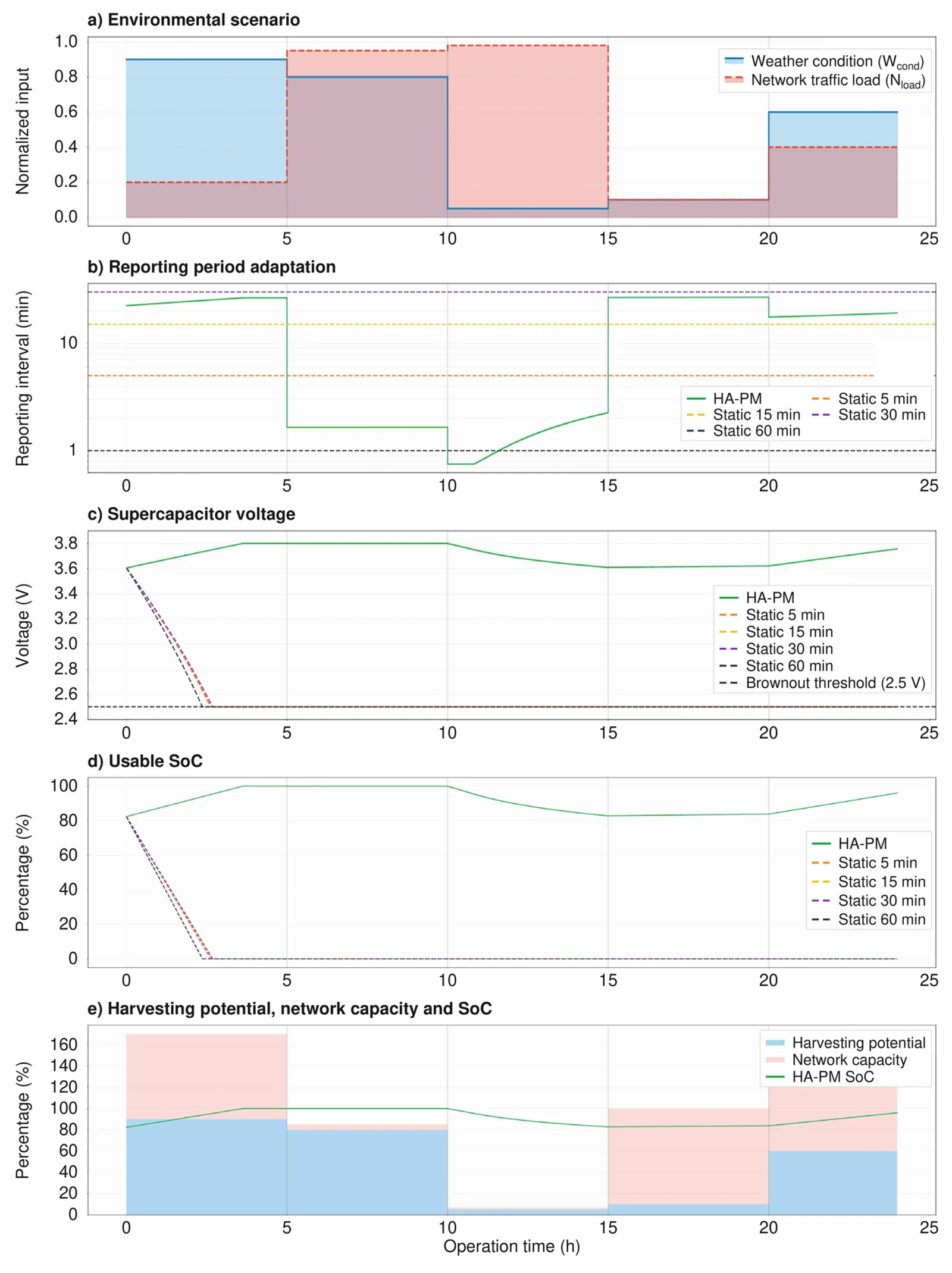
Multi-phase stress-test performance under adverse harvesting and network traffic conditions.

Figure 12 presents the multi-phase stress-test behavior of HA-PM and representative fixed synchronization baselines. The upper panel defines the time-varying stress inputs, including changes in harvesting condition and network traffic. The reporting period panel shows that HA-PM dynamically adjusts $T_{sync}\left(t\right)$ in response to these changes, whereas the fixed baselines remain insensitive to environmental and traffic variations. This difference is important because static reporting periods cannot react to temporary energy scarcity or traffic congestion.

The supercapacitor voltage response shows that frequent fixed synchronization baselines deplete rapidly during the adverse phases of the scenario. In contrast, HA-PM maintains the voltage above the minimum operational threshold throughout the simulated stress horizon by extending the reporting period and increasing low-power residency when the energy condition deteriorates. This result indicates that adaptive synchronization can reduce the risk of threshold crossing during short-term adverse events.

The corresponding SoC profile further supports this interpretation. Static baselines with short reporting periods exhibit rapid SoC collapse because repeated communication events dominate the energy budget. HA-PM, by contrast, preserves a higher usable SoC during the stress sequence. This behavior is consistent with the control objective of HA-PM: balancing responsiveness and energy preservation rather than maintaining a fixed communication schedule under all operating conditions.

The energy-balance panel illustrates the interaction among harvesting potential, network demand, and HA-PM control response. During low-harvesting or high-load periods, HA-PM compensates by increasing $T_{sync}\left(t\right)$, thereby reducing communication-related energy consumption. During recovery phases, the controller gradually returns toward shorter reporting periods as the energy condition improves. This behavior demonstrates context-aware adaptation under adverse operating conditions.

**Figure 13:**
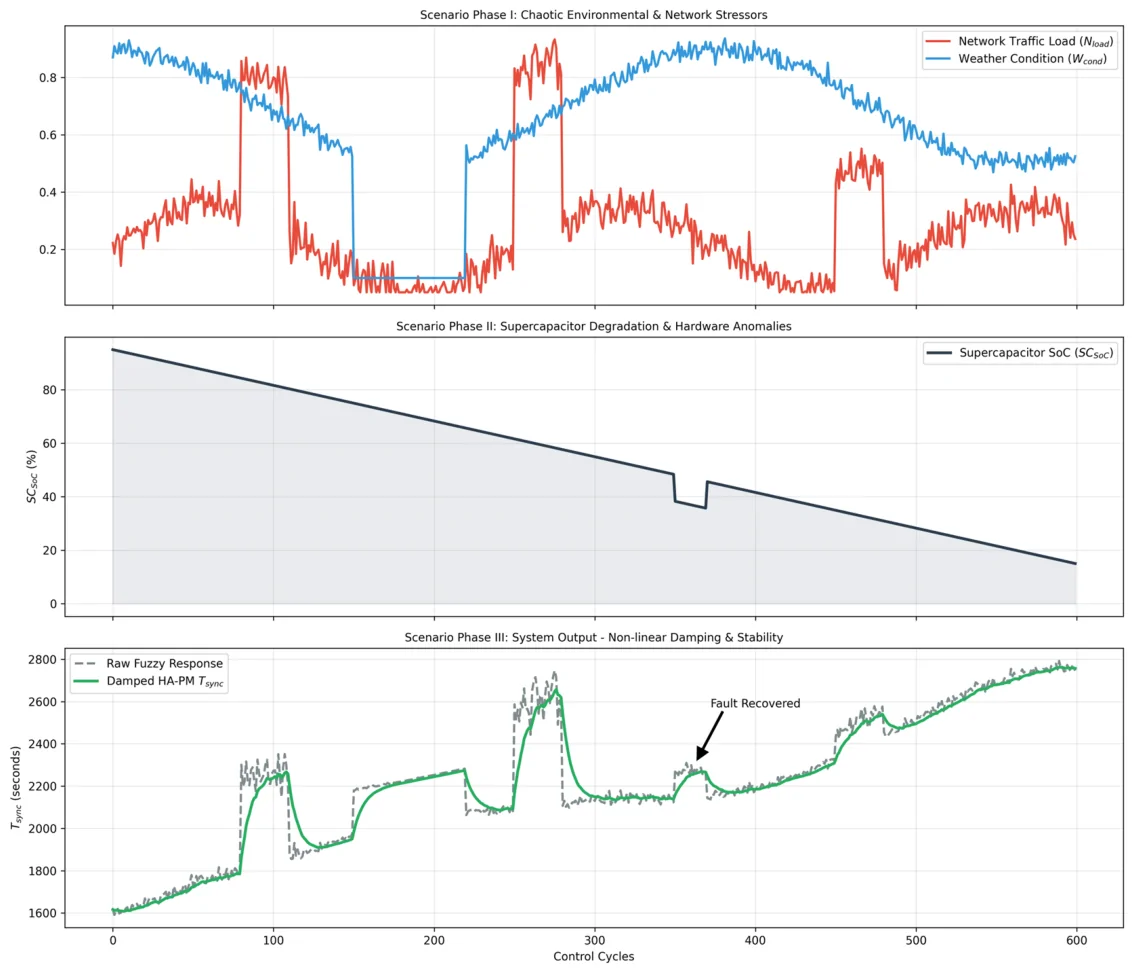
HA-PM response under stochastic environmental, network traffic, and SoC disturbances.

Figure 13 further evaluates HA-PM under simultaneous stochastic traffic fluctuation, weather variation, supercapacitor degradation, and a transient SoC disturbance. The first panel shows noisy environmental and network traffic inputs. The second panel shows progressive SoC degradation with a temporary disturbance event. The third panel compares the raw fuzzy response with the damped HA-PM synchronization response. The damped response follows the overall control trend while reducing high-frequency fluctuations, indicating smoother reporting period adaptation under noisy operating conditions.

The transient SoC disturbance around t=350 provides an additional stress case. After the disturbance, the HA-PM response returns to a stable synchronization trajectory rather than producing persistent oscillatory behavior. This suggests that the smoothing mechanism can reduce control chattering and unnecessary power-state transitions under the evaluated stochastic scenario. However, this result should be interpreted as simulation-based evidence of disturbance tolerance, not as a formal proof of global stability.

The stress-test results show that HA-PM improves operational resilience relative to frequent fixed synchronization baselines under the evaluated stress scenarios. The improvement arises from dynamic reporting period adaptation and the resulting increase in low-power residency during adverse phases. The observed resilience remains conditional on the specified harvesting model, traffic-load pattern, supercapacitor parameters, power-mode assumptions, damping mechanism, and FLC rule base.

## 5. Discussion

The HA-PM framework provides a context-aware energy management strategy for energy-harvesting LPWAN IoT nodes. Unlike fixed reporting policies, HA-PM adapts the reporting period according to supercapacitor SoC, network traffic load, and weather/control-context information. This adaptive behavior enables the system to increase low-power residency during energy-constrained or high-load periods while allowing shorter reporting periods when the operating conditions are more favorable.

The FLC decision-surface analysis shows that the controller produces interpretable synchronization decisions across different operating regions. In particular, low SoC and adverse operating conditions lead to longer reporting periods, whereas favorable energy and workload conditions allow shorter periods. This behavior is consistent with the intended objective of HA-PM: balancing energy preservation and communication responsiveness rather than optimizing either objective independently.

The energy-consumption and mode-residency results indicate that the energy benefit of HA-PM is primarily associated with reduced communication frequency and increased residency in low-power modes. These findings support the proposed coupling between gateway-side synchronization control and node-side EH-DPM mode selection. The gateway-derived reporting period defines the available sleep opportunity, while the node-side policy selects the lowest-power feasible operating mode according to the corresponding transition constraints.

The supercapacitor SoC and voltage analyses indicate that HA-PM can preserve operational continuity more effectively than frequent fixed synchronization baselines in the evaluated scenarios. However, these results should be interpreted as conditional simulation-based outcomes. The reported lifetime values depend on the assumed harvesting model, leakage current, active synchronization duration, power-mode parameters, and supercapacitor characteristics. Right-censored lifetime results indicate only that the minimum operational voltage was not reached within the simulated horizon; they do not establish a universal hardware lifetime.

The stress-test analysis further shows that HA-PM can respond to abrupt adverse conditions by increasing the reporting period and reducing communication-related energy demand. This behavior is relevant to LPWAN deployments exposed to variable energy harvesting, traffic bursts, and temporary environmental degradation. Nevertheless, the stress-test results remain scenario-dependent and should be validated using additional measured environmental and network traces before broader generalization.

The sensitivity results show that long-horizon operational continuity is influenced by harvested energy and synchronization-related workload assumptions. This finding reinforces the need to interpret the reported lifetime values together with the underlying simulation parameters. In practical deployments, operational continuity may also depend on component selection, installation geometry, temperature, storage aging, converter efficiency, and communication workload.

Overall, HA-PM should be interpreted as an adaptive energy management framework rather than as a universal lifetime-maximization mechanism. Its principal contribution is the provision of an interpretable and context-aware trade-off among energy preservation, synchronization responsiveness, and operational continuity. This makes the framework relevant to energy-harvesting LPWAN IoT scenarios in which operating conditions vary over time and fixed synchronization policies lack sufficient adaptability.

## 6. Conclusions

This study presents HA-PM as a model-based framework that connects adaptive reporting with local service obligations in energy-harvesting LPWAN IoT nodes. The gateway employs fuzzy logic to determine a nominal reporting period from the supercapacitor’s usable SoC, observed network traffic, and the current weather/control context. The node then selects the power mode and task-scheduling plan with the lowest estimated total node energy from a defined finite set of feasible candidates. By centralizing the inference process, the system achieves interpretable, context-aware energy management without imposing a significant computational burden on the nodes. All candidate plans share the same mandatory work, comparison horizon, and equivalent terminal conditions, making energy selection conditional on comparable service obligations. This coordination allows the reporting calendar to adapt while maintaining the required sensing and local timing conditions within the planning decision.

HA-PM integrates established lines of research on harvesting-aware communication timing, fuzzy context assessment, and dynamic power management. It provides a practical balance among energy preservation, synchronization responsiveness, and operational continuity for energy-harvesting LPWAN IoT nodes. Its methodological contribution is the explicit connection between these decisions: a proposed communication interval defines a scheduling constraint, while local planning determines task execution and low-power residence. Total-plan energy accounting ties each candidate’s estimated cost to the work and state changes required to execute it, including the cost of the local controller’s own activity. The predefined fuzzy rules and explicit feasibility conditions also leave the connection between reporting preferences and local operating choices open to inspection. For LPWAN IoT designs, this structure supports the formulation of reporting policies around application workloads, including data acquisition and processing that remain due between reports. This provides a way to consider available energy alongside the services that the application requires.

Future work may investigate how alternative reporting policies interact with application-specific task planning while retaining the separation between gateway context inference and node feasibility assessment. Such studies could vary sensing workloads and reporting requirements to examine how they shape the feasible plan set and its estimated energy costs under a common service definition. Overall, HA-PM contributes a structured basis for defining and comparing energy-aware LPWAN operating choices in relation to explicit application service obligations.

## Figures and Tables

**Table 1:** System power modes of the LPWAN IoT node (FiPy, Pycom Ltd., UK).

Power Mode	MCU Domain	Memory Domain	Peripherals Domain	RTC Domain	Communication Domain			
					LTE	Wi-Fi	BLE	LoRa
M0	Active	Active	Active	Active	Active	Active	Active	Active
M1	Active	Active	Active	Active	Active	Active	Active	Sleep
M2	Active	Active	Active	Active	Active	Active	Disable	Active
M3	Active	Active	Active	Active	Sleep	Active	Active	Active
M4	Active	Active	Active	Active	Active	Active	Disable	Sleep
M5	Active	Active	Active	Active	Sleep	Active	Active	Sleep
M6	Active	Active	Active	Active	Active	Disable	Active	Active
M7	Active	Active	Active	Active	Active	Disable	Active	Sleep
M8	Active	Active	Active	Active	Active	Disable	Disable	Active
M9	Active	Active	Active	Active	Sleep	Active	Disable	Active
M10	Active	Active	Active	Active	Active	Disable	Disable	Sleep
M11	Active	Active	Active	Active	Sleep	Active	Disable	Sleep
M12	Active	Active	Active	Active	Sleep	Disable	Active	Active
M13	Active	Active	Active	Active	Sleep	Disable	Active	Sleep
M14	Active	Active	Active	Active	Sleep	Disable	Disable	Active
M15	Clock-gated	Active	Active	Active	Idle	Idle	Idle	Standby
M16	Clock-gated	Active	Active	Active	Sleep	Disable	Disable	Sleep
M17	Clock-gated	Standby	Standby	Active	Sleep	Disable	Disable	Sleep
M18	Light-sleep	Sleep	Standby	Active	Sleep	Disable	Disable	Sleep
M19	Deep-sleep	Sleep	Sleep	Active	PSM	Disable	Disable	Sleep

**Table 2:** Power-mode and transition parameters.

Modes	Power (W)	Entry and Exit s Each	Entry and Exit J Each	Tth (ms)
M0	1.73	0	0	0
M1	1.64	0	0	0
M2	1.54	0	0	0
M3	1.45	0	0	0
M4	1.35	0	0	0
M5	1.25	0	0	0
M6	1.16	0	0	0
M7	1.06	0	0	0
M8	0.96	0	0	0
M9	0.87	0	0	0
M10	0.77	0	0	0
M11	0.67	0	0	0
M12	0.58	0	0	0
M13	0.48	0	0	0
M14	0.38	0	0	0
M15	0.3	0.0025	0.0043	7
M16	0.26	0.005	0.009	15
M17	0.11	0.01	0.017	30
M18	0.014	0.09	0.16	189
M19	0.0007	0.15	0.26	300

**Table 3:** Consequents of the 27-rule reporting-period controller.

Rules	S_SoC_	N_load_	W_cond_: Poor	W_cond_: Average	W_cond_: Good
1–3	Low	Low	Medium	Medium	Medium
4–6	Low	Normal	Long	Long	Medium
7–9	Low	High	Long	Long	Long
10–12	Medium	Low	Medium	Short	Short
13–15	Medium	Normal	Medium	Medium	Short
16–18	Medium	High	Long	Medium	Medium
19–21	High	Low	Medium	Short	Short
22–24	High	Normal	Medium	Short	Short
25–27	High	High	Medium	Medium	Medium

**Table 4:** The closed-loop reference simulation settings.

Setting	Declared Value
Controlled system	Energy-harvesting nodes, gateway context, and exogenous background packets
Observation window	259200 s (72 h); matched seeds 1101–1108
Experimental matrix	A–D in six scenarios, three input-removal variants in mixed traffic only; two buffer profiles; $2\times 8\times \left(6\times 4+3\right)=432$
Arms A, B, C, D	A: fixed period + baseline selector; B: fixed period + total-energy selector; C: FLC + baseline selector; D: FLC + total-energy selector
Baseline selector	Lowest-residence-power feasible EARLIEST candidate
Proposed selector	Minimum estimated total plan energy among 40 EARLIEST/LATEST × M0–M19 candidates
Reporting commands	Default 900 s; range 60–3600 s; downward quantum 1 ms; timeout 1800 s
Sensing and processing	Sensing every 60 s, duration 0.02 s, deadline 0.1 s, start slack 0.01 s; processing duration 0.04 s, deadline 0.2 s
Receiver service	Every 300 s; duration 0.1 s; gateway control opportunity at the receiver-window start
Event model	Rate (1/600) s^−1^ outside mixed traffic; mixed alternates (1/1800) s^−1^ and (1/30) s^−1^ every 12 h, with three simultaneous events at (6 + 12k) h; persistence: 120 s
Background traffic	Rate 1/60 s^−1^ outside mixed traffic; mixed alternates 1/120 and 1/12 s^−1^; 0.12 s packet airtime
Queue and packet capacity	1000 ten-byte records; FIFO with drop-newest overflow; maximum packet 250 bytes
Radio	Two 0.12 s uplink copies; two 0.04 s downlink copies, 0.05 s apart; target-copy random loss 0.1 plus interval-overlap and availability checks
Context and harvest	Independent prescribed draws W∼U[0.3,1] per 300 s bin; $P_{h}=0.025max[0,sin\left(\pi \left(h-6\right)/12\right)]W$ W, where h is hour of day; harvest set to zero in no-harvest scenario
Stressors	Gateway outage [12 h,48 h); temperature profile $25+10sin\left(2\pi \left(h-9\right)/24\right)$ °C, plus 20 °C in hot; leakage multiplied by 10 in high-leakage scenario
Storage profiles	Base capacitance 270 F, capacity 1949.4 J, initial energy 1749.6 J, brownout 843.75 J, restart 1215 J; 8× scales these and reference leakage together; absolute reserve remains 5 J
Leakage	$I_{leak}=I_{ref}\left(V/V_{ref}\right)2^{\left(T-25\right)/10};$ base $I_{ref}=20$ µA, $V_{ref}=3.8$ V
Gateway energy	Baseline 1 W; 0.001 J per available-gateway compute attempt, including stale-input attempts; modeled TX 0.5 W and RX 0.05 W over their accounted airtimes

## Data Availability

The data presented in this study are available from the corresponding author upon reasonable request.

## Supplementary Materials

The following supporting information can be downloaded at: https://www.mdpi.com/article/10.3390/s26196257/s1.
