# Supplementary material for: Gateway-Driven Hybrid Adaptive Power Management for Energy-Harvesting LPWAN IoT Nodes Using Fuzzy Logic
Source: Sensors (Basel). 2026 Oct 2;26(19):6257. doi: 10.3390/s26196257 (PMC13667690; doi:10.3390/s26196257)
Supplement: Supplementary file 1 [file sensors-26-06257-s001.zip › sensors-4488276-supplementary.pdf]

Supplementary Material

Gateway-Driven Hybrid Adaptive Power Management for Energy-Harvesting LPWAN IoT Nodes Using Fuzzy Logic

Table of Contents

S1 Current Controller Definition ... 6
S1.1 Gateway information and command application ... 6
S1.2 Membership coordinates and domain coverage ... 6
S1.3 Rule aggregation and bounded command formation ... 8
S1.4 Finite energy-minimizing catalogue ... 10
S1.5 Persistent service identities and terminal equivalence ... 10
S1.6 Causal sensing, processing and radio payloads ... 11
S1.7 Continuous storage accounting and first crossing ... 11
S1.8 Paid interruption and boundary ordering ... 12
S1.9 Controlled near-service recovery ... 12
S1.10 Model validation and controlled failure outcomes ... 13
S2 Static Controller Diagnostics ... 13
S2.1 Directional reversals and central controller slices ... 13
S2.2 Operating-point commands and finite differences ... 15
S2.3 Quantization and exact-input comparisons ... 16
S3 Ablation Study ... 17
S3.1 Structural input removal and static effect magnitudes ... 17
S3.2 Reduced-controller command ranges ... 17
S3.3 Mixed-workload ablation energy and delivery ... 18
S3.4 Absolute outcomes under input removal ... 21
S3.5 Historical structural input removal ... 21
S3.6 Midpoint input clamps in the 24 h model ... 23
S3.7 Central-reference information neutralization ... 25
S3.8 Reference robustness and conditional input influence ... 25
S3.9 Conditional energy under invariant M19 selection ... 29
S4 Current Evaluation Protocol ... 29
S4.1 Policy factors and matched exogenous traces ... 29
S4.2 Scenario design and the completed cohort ... 30
S5 Energy and Service Outcomes ... 31
S5.1 Energy and service across the four arms ... 31
S5.2 Complete retained-profile outcome census ... 35

S5.3 Complete larger-buffer outcome census ... 37
S5.4 Paired selector effects and interaction ... 39
S6 Leakage and Outage Behavior ... 43
S6.1 Leakage and first-brownout observations ... 43
S6.2 Outage voltage, period and queue trajectories ... 44
S6.3 Outage recovery endpoints ... 47
S6.4 Node, loss and gateway energy boundaries ... 48
S7 Radio and Communication Costs ... 49
S7.1 Downlink and receiver costs ... 49
S7.2 Direct radio effects within the current campaign ... 50
S7.3 Fixed-trace downlink collision replay ... 52
S8 Implementation and Verification ... 53
S8.1 Common-service selector replay ... 53
S8.2 Bounded implementation resource requirements ... 53
S8.3 Exact scheduling representation and recovery additions ... 54
S8.4 Recovery sensitivity fixtures ... 55
S8.5 Controller fixtures and independent energy oracle ... 55
S8.6 Command lattice residuals and exact recurrences ... 56
S8.7 Retained centroid-reference checks and unresolved integration step ... 56
S8.8 Historical eight-job compiled subsystem ... 57
S8.9 Reported numerical consistency and execution provenance ... 57
S9 Parameter and Evidence Registers ... 58
S9.1 Mode powers and their evidence boundary ... 58
S9.2 Inherited transition conventions and admission units ... 59
S9.3 Sensing, processing and radio service settings ... 62
S9.4 Storage and reserve assumptions ... 63
S9.5 Inherited storage and incremental service evidence ... 64
S9.6 Synthetic environment and leakage settings ... 65
S9.7 Physical events and channel realization ... 66
S9.8 Gateway observation and command timing ... 67
S9.9 Requested service and bounded queue ... 68
S9.10 Blind-copy transactions and receive windows ... 68

S9.11 Node and gateway energy increments 69
S9.12 Numerical criteria and causal execution semantics 70
S10 72 h Evaluation 71
S10.1 Four-arm energy and sensing 71
S10.2 Selector interaction 73
S10.3 Outage and restart behavior 74
S10.4 Communication boundary 77
S11 Independent 24 h Network Evaluation 77
S11.1 Finite static diagnostics in the 24 h model 77
S11.2 Nominal energy and physical-event delivery 80
S11.3 Complete physical-event outcome partition 81
S11.4 Complete node-time occupancy 82
S11.5 Stress-family outcomes 83
S11.6 Command transport and receive/fallback diagnostics 84
S11.7 Predesignated combined-stress node histories 87
S11.8 Control and transition sensitivity 88
S11.9 Environmental and channel sensitivity 91
S11.10 Local-weight response under nominal and burst workloads 93
S11.11 Censor-aware 24 h horizon outcomes 93
S11.12 Device and service assumptions in the 24 h model 95
S11.13 Control, storage and communication assumptions 96
S11.14 Membership, stress and analysis settings 97
S11.15 Storage-side energy accounting in the 24 h model 98
S12 Bounded-Input Numerical Replay 99
S12.1 Bounded numerical properties and their limits 99
S12.2 Prespecified bounded-input replay design 100
S12.3 Raw-output variation across stochastic replays 101
S12.4 Projected-storage threshold diagnostics 103
S12.5 Externally forced alternating replay 106
S13 Deterministic Prolonged Outage Study 108
S13.1 Primary deterministic prolonged-outage case 108
S13.2 Complete deterministic outage domain 110

S14 Provisional Thermal Diagnostic 112
S15 Energy Consumption Distributions and Variability 113

S1 Current Controller Definition

S1.1 Gateway information and command application

The gateway proposes reporting periods from information that has reached it, while the node retains responsibility for execution. Usable-energy state of charge (SoC) is sampled at the actual start of a target transmission and cached only after its first successful delivery. Traffic counts successfully received background packets and first deliveries of target reports during the preceding 3600 s, normalized by 60 and capped at one. Duplicate report copies do not increase that count. The context input is the current prescribed trace value and supplies no credit for future harvesting. These signals therefore represent delivered energy information, observed network activity, and contemporaneous external context, respectively.

Control opportunities occur at 299, 599, and subsequent 300 s intervals. Gateway outage, absent telemetry, or SoC older than 7200 s suppresses command creation; equality at the age limit remains eligible. Invalid inference instead raises an explicit FLCError, as specified in the main manuscript's Methods. Each valid opportunity creates one sequenced command and schedules two predetermined copies, irrespective of first-copy success. Generation, transmission, reception, validation, and application remain distinct events. The node initially uses a 900 s period and revalidates received commands at the next nominal transmission-completion boundary. An applied command governs subsequent periods without replacing the current deadline. This interface explains why a proposed reporting period constrains the local schedule but does not itself specify sleep duration or demonstrate completed service.

S1.2 Membership coordinates and domain coverage

The numerical controller uses twelve piecewise-linear membership sets across three inputs and one output. Table S1 preserves every coordinate, including zero-membership endpoints; Figure S1 shows the corresponding geometry. SoC spans 0–100%, normalized traffic and context each span 0–1, and the output period spans 60–3600 s. Adjacent input sets overlap, while endpoint shoulders cover the domain boundaries. Thus, linguistic categories represent overlapping degrees of membership rather than disjoint operating regions.

The central SoC set peaks at 50%, and the central traffic and context sets peak at 0.5. Their supports nevertheless differ, so a common central coordinate does not make their response functions interchangeable. Similarly, the Short output has a plateau from 60 to 200 s, whereas Medium peaks at 1800 s and Long reaches unit membership at 3600 s. These definitions specify the numerical profile underlying the supplementary diagnostics and expand the membership-function description in the main manuscript. The coordinates are adopted modeling assumptions; they do not recover the exact breakpoints of the historical Java evaluation or establish calibrated hardware thresholds.

Table S1. Complete membership coordinates for the twelve input and output sets. Coordinates specify variable value and membership degree; output values are seconds.

Table S1		
**Variable**	**Set**	**Coordinates**
soc	Low	(0, 1); (35, 0); (100, 0)

Table S1		
**Variable**	**Set**	**Coordinates**
soc	Medium	(0, 0); (20, 0); (50, 1); (80, 0); (100, 0)
soc	High	(0, 0); (65, 0); (90, 1); (100, 1)
traffic	Low	(0, 1); (0.4, 0); (1, 0)
traffic	Normal	(0, 0); (0.2, 0); (0.5, 1); (0.8, 0); (1, 0)
traffic	High	(0, 0); (0.6, 0); (1, 1)
weather	Poor	(0, 1); (0.4, 0); (1, 0)
weather	Average	(0, 0); (0.25, 0); (0.5, 1); (0.75, 0); (1, 0)
weather	Good	(0, 0); (0.6, 0); (1, 1)
output	Short	(60, 1); (200, 1); (1200, 0); (3600, 0)
output	Medium	(60, 0); (800, 0); (1800, 1); (2800, 0); (3600, 0)
output	Long	(60, 0); (2400, 0); (3600, 1)

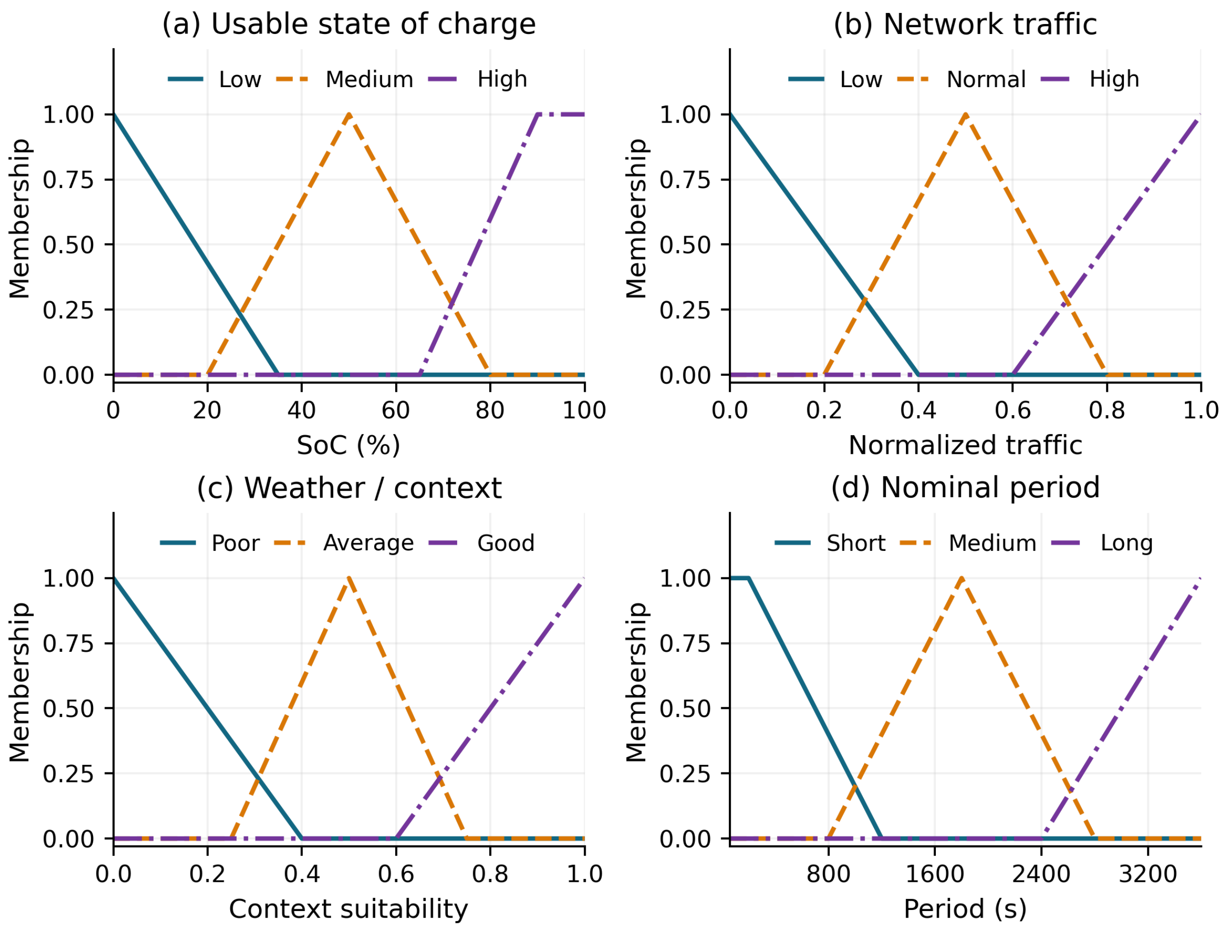

Figure S1. Membership functions for (a) usable SoC, (b) normalized gateway traffic, (c) context suitability, and (d) nominal period. Lines connect the declared coordinates; the four panels retain all linguistic sets.

S1.3 Rule aggregation and bounded command formation

The full controller retains all 27 combinations of the three linguistic input categories in Table S2. Minimum conjunction determines each rule's activation, and minimum implication clips its consequent membership function. Bounded summation then combines every active contribution, including conflicting recommendations. No winning rule is selected. In the displayed expressions, α denotes activation, μ denotes membership, and z is a candidate period in seconds. The continuous aggregate is integrated analytically between 60 and 3600 s, preserving membership knots, clipping intersections, and saturation crossings.

$$\alpha_r = min(\mu_A(s), \mu_B(n), \mu_C(w)) \tag{1}$$

$$\mu_r(z) = min(\alpha_r, \mu_{Dr}(z)), \ \mu(z) = min(1, \Sigma\, \mu_r(z)) \tag{2}$$

$$T_{raw} = \frac{\int_{60}^{3600} z\, \mu(z)\, dz}{\int_{60}^{3600} \mu(z)\, dz} \tag{3}$$

$$T_{cap} = min(3600, T_{service,max}), \ T_0 = clip(T_{raw}, 60, T_{cap}) \tag{4}$$

$$T_{cmd} = T_0 + \mu_{Low}(s)\, (T_{cap} - T_0) \tag{5}$$

The centroid is bounded by the lower limit and the service cap before the Low-SoC envelope is applied. The reference campaign supplies no additional service cap, leaving the upper bound at 3600 s. The displayed Tcmd denotes the envelope output before transmission quantization. Applying the same bounded 1 ms downward quantizer to the base and enveloped outputs preserves the same-input nonshortening property. That property does not establish global SoC monotonicity, energy neutrality, or closed-loop convergence. Nonfinite or out-of-domain inputs, invalid aggregate area, or a nonfinite moment raise FLCError under the main manuscript's inference contract. These equations elaborate its Mamdani inference mechanism; physical service and energy feasibility remain separate node-side decisions.

Table S2. Complete 27-rule mapping from SoC, traffic, and context categories to output consequents. All applicable rows contribute to bounded-sum aggregation.

Table S2				
**Rule**	**SoC**	**Traffic**	**Context**	**Consequent**
1	Low	Low	Poor	Medium
2	Low	Low	Average	Medium
3	Low	Low	Good	Medium
4	Low	Normal	Poor	Long
5	Low	Normal	Average	Long
6	Low	Normal	Good	Medium
7	Low	High	Poor	Long
8	Low	High	Average	Long
9	Low	High	Good	Long
10	Medium	Low	Poor	Medium
11	Medium	Low	Average	Short
12	Medium	Low	Good	Short
13	Medium	Normal	Poor	Medium
14	Medium	Normal	Average	Medium
15	Medium	Normal	Good	Short
16	Medium	High	Poor	Long
17	Medium	High	Average	Medium
18	Medium	High	Good	Medium
19	High	Low	Poor	Medium

Table S2				
**Rule**	**SoC**	**Traffic**	**Context**	**Consequent**
20	High	Low	Average	Short
21	High	Low	Good	Short
22	High	Normal	Poor	Medium
23	High	Normal	Average	Short
24	High	Normal	Good	Short
25	High	High	Poor	Medium
26	High	High	Average	Medium
27	High	High	Good	Medium

S1.4 Finite energy-minimizing catalogue

The current node minimizes estimated energy within a finite catalogue of admissible plans. Two fixed ordering rules, EARLIEST and LATEST, are crossed with twenty homogeneous residence modes, M0–M19, giving forty candidates. Services execute in M0, and every candidate returns to M0 at its terminal boundary. Residence mode therefore changes the intervals between services without changing the required workload used to compare candidates.

The objective includes control expenditure, service energy, mode entry and exit, residence consumption, and the declared estimated loss. Candidates are ordered by total estimated energy, then transition count, then lexical identifier. This deterministic tie handling permits repeatable comparisons. The baseline instead considers feasible EARLIEST plans and selects the mode with minimum residence power. M19 remains an ordinary candidate subject to admission, at catalogue index 19; it is not an unconditional fallback. The locked 7, 15, 30, 189, and 300 ms thresholds are policy admissibility conditions rather than experimentally measured break-even durations.

This definition makes the manuscript's energy-minimizing local decision precise. Its optimum is conditional on the enumerated schedules, fixed workload, terminal equivalence, and estimated cost model. It neither optimizes over unrestricted job orderings and mixed-mode trajectories nor proves that physical energy reserves will remain sufficient. A rejected catalogue can coexist with some feasible schedule outside the supplied search space.

S1.5 Persistent service identities and terminal equivalence

The current service calendar preserves job identity across assessments and execution. A persistent JobBook records immutable releases, deadlines, predecessors, and payload membership for sensing, processing, reception, and report transmission. Reconsidering a plan therefore does not create a fresh version of work that has already completed or missed its opportunity. The report boundary is a completion target, not permission to move earlier services retroactively.

For example, the 300 s receive stream with phase −1 s first occupies 299–299.1 s. A report associated with target d is released at d−0.24 s and must complete exactly at d. At assessment horizon H, required work includes jobs whose deadlines are at most H or whose latest starts precede H, together with their necessary ancestors. Other pending jobs retain their original definitions; deadlines beyond H are not clipped to H. Nonpreemptive service cannot straddle the terminal boundary.

Candidate equivalence requires M0 and the complete service-workload fingerprint: required definitions, actual completed and missed outcomes, pending work, data, and payload state. Energy is excluded from that fingerprint so that genuinely equivalent service can have different costs. These rules substantiate the manuscript's local energy comparison while preventing a cheaper candidate from silently dropping obligations. A projected completion is not an actual completion, and feasibility of the supplied schedule does not establish global scheduling feasibility.

S1.6 Causal sensing, processing and radio payloads

Physical data availability constrains what the current node can transmit. Each deterministic sensing output has an exact processing predecessor. Completion callbacks retain the producing process identifier and actual sensing time, allowing an observation to be traced through sensing, processing, queuing, and reporting. These relationships distinguish service completion from information delivered to the gateway.

At the actual start of a transmission, the node freezes a FIFO batch of ready observations. Only completed processing outputs or explicitly preprocessed items can enter that batch. Observations that become ready later cannot join a transmission already in progress. An empty telemetry report remains admissible, and a mandatory report does not acquire every pending processing job as a predecessor. Thus report obligations and data availability remain related but distinct parts of the workload.

Each report uses two predetermined copies without acknowledgements. Completion removes the frozen local batch irrespective of hidden channel delivery, whereas the gateway records reception at its own causal delivery time. Physically funded nonpreemptive completions are committed together at their actual boundary. A miss is recorded only after the actual latest-start opportunity has passed; OFF operation cannot fabricate completions or backdate them. These mechanisms qualify the manuscript's energy and delivery interpretation: a completed radio transaction need not deliver data, and lower node consumption can accompany fewer observations or lost reports. Comparing energy alone therefore does not establish efficiency for equal successfully delivered information.

S1.7 Continuous storage accounting and first crossing

The physical storage kernel advances energy continuously between discrete events. Within each segment, harvesting and powered loads enter a linear differential balance, while leakage is proportional to stored energy. Its coefficient combines reference leakage current, capacitance, reference voltage, the leakage multiplier, and the declared temperature factor. Exact rational segment durations are converted once to binary floating point for this physical integral; exact scheduling does not imply exact real-number energy arithmetic.

The first continuous crossing of the operating threshold interrupts service even when an endpoint-only calculation would conceal the crossing. Physical evolution continues through the remaining OFF interval, but interrupted work does not gain a completion. A partially elapsed phase is censored as ongoing work rather than being recast as a completed M0 terminal or a restart checkpoint.

The physical ledger separates gross harvested energy, discarded overflow, node load, leakage, and other loss, whose balance equals the stored-energy change. A reserve is an admission condition rather than an additional expenditure, and future harvesting is not credited to a present decision. The planner's 1 mW estimated loss remains an assumption, not a certified upper bound on physical loss. This separation supports the manuscript's accounting interpretation while retaining brownout and missed-service outcomes. It also prevents importing the unresolved 7.63 J discrepancy from the separate thermal diagnostic as a compensating constant in the current kernel.

S1.8 Paid interruption and boundary ordering

An interrupt changes the next permissible action without erasing energy or time already committed. Current control, service, mode entry, and mode exit are nonpreemptive phases. Environmental changes can split physical integration segments, but they do not supply advance information to the planner. Interrupts arriving during a committed phase are latched until the appropriate boundary.

At an M0 boundary, a latched event is handled before a new entry or nominal command application. If entry has already begun, its completion is followed by a full paid exit when an interrupt is pending. An interrupt during residence starts one exit; another event during that exit is latched rather than initiating a duplicate transition. The accepted command retains its original reception timestamp throughout, so deferred handling does not renew its freshness.

The retained boundary fixture makes this distinction concrete. An event at 0.1 s arrives during entry from 0.061 to 0.211 s and is handled at 0.361 s after the exit from 0.211 to 0.361 s. An event exactly at 0.061 s is processed before entry begins. These timings support the manuscript's implementation claim that transitions carry actual residence and expenditure. They do not measure processor interrupt latency or guarantee an immediate service response. Counting the interrupt as a free escape would change both the energy comparison and the physically available service interval.

S1.9 Controlled near-service recovery

Controlled recovery follows rejection of the full service horizon; it does not redefine that rejected plan as feasible. The node first assesses the complete horizon, then forms a common near horizon from the full target, the current time plus 60 s, and the first viable deadline plus a 1 s tail. When the near horizon reaches the full target, an idle prefix can precede the next release. Otherwise the candidate horizon must lie beyond the two-search control interval and before the original full horizon.

Near candidates retain the common job definitions, M0 terminal, and requested selector. An attempted full search followed by a near search, including an unsuccessful near search, incurs 2 ms of control time, two 10 μJ incremental fees, M0 power, and estimated loss. If admission fails before the second search, only the original full-search cost is charged.

A selected recovery prefix retains the original full-horizon rejection and service accounting. Missed work remains missed; a valid idle prefix or bounded M0/OFF wait does not manufacture completion. Restart behavior is separate, and the next nominal report target remains d plus the applied period. These rules explain the manuscript's response to distant scheduling conflicts without claiming that every full horizon becomes feasible. Retained rejection summaries support that conclusion, but they are not a complete export of every candidate phase from every campaign run.

S1.10 Model validation and controlled failure outcomes

A controlled failure distinguishes a malformed model from an unsupported state or an infeasible workload. Current validation checks finite values, types, domains, and ordering before admitting a configuration or calendar update. Boolean values are rejected where physical numeric quantities are required. An unknown energy value remains unknown and prevents selection rather than being silently replaced with zero or an optimistic estimate.

The configuration is copied and frozen; a change requires explicit replacement rather than mutation during execution. InvalidModel identifies malformed inputs. UnsupportedState identifies an otherwise valid state outside the implemented initial-mode contract. NO_FEASIBLE_PLAN identifies valid work for which the supplied candidate catalogue has no admissible plan. These outcomes preserve the reason for failure and do not imply the same physical event.

Registration and assessment must fail without partial mutation. A missing or missed ancestor cannot be converted into a successful predecessor to make a descendant admissible. Valid iterable inputs remain supported, while malformed public inputs produce a controlled error. These protections make the manuscript's local algorithm reproducible within its stated domain. They do not establish that every numeric input is physically calibrated, that every deployment can be scheduled, or that all rejected workloads are globally impossible. In particular, the 60 s near horizon and 1 s deadline tail are declared recovery assumptions; input validation does not transform those choices into measured optimal settings.

S2 Static Controller Diagnostics

S2.1 Directional reversals and central controller slices

The intended period is nonincreasing with SoC, nondecreasing with traffic, and nonincreasing with improving context. These directions fail on the deterministic 21^3 grid: 9261 triples and 8820 adjacent pairs per axis. At 10^{-8} s tolerance, Table S3 reports 407/585/686 quantized reversals for SoC/traffic/context, versus 281/508/611 unquantized reversals. Numerical floor boundaries therefore explain only some violations.

Three counterexamples use coordinates (SoC%, traffic, context). Changing (65,0.65,0.8) to (70,0.65,0.8) increases the period from 959.423 to 1142.265 s. Changing (80,0.35,0.35) to (80,0.40,0.35) decreases it from 1150.070 to 1022.030 s. Changing (80,0.65,0.60) to (80,0.65,0.65) increases it from 959.423 to 1150.070 s. Figure S2 retains central slices and the SoC example; Table S4 adds axis spans, including zero minima for traffic and context. These findings bound the manuscript's directional interpretation. Same-input envelope nonshortening does not imply ordering across inputs. Deterministic grid points provide neither sampling confidence intervals nor closed-loop stability evidence.

Table S3. Directional violations before and after quantization, largest reversal, and largest absolute adjacent step; 8820 neighboring pairs are evaluated per input.

Table S3				
**Input**	**Quantized reversals**	**Unquantized reversals**	**Largest reversal s**	**Largest absolute step s**
soc	407	281	182.842	1014.532

Table S3				
**Input**	**Quantized reversals**	**Unquantized reversals**	**Largest reversal s**	**Largest absolute step s**
traffic	585	508	128.040	623.064
weather	686	611	190.647	767.702

Table S4. Full-controller reversals and minimum, median, and maximum axis spans, in seconds, over the deterministic grid.

Table S4			
**Input**	**Reversals**	**Largest reversal s**	**Span min median max s**
soc	407	182.842	1800.000 / 2192.606 / 3154.896
traffic	585	128.040	0.000 / 1224.737 / 1400.000
weather	686	190.647	0.000 / 1072.452 / 1400.001

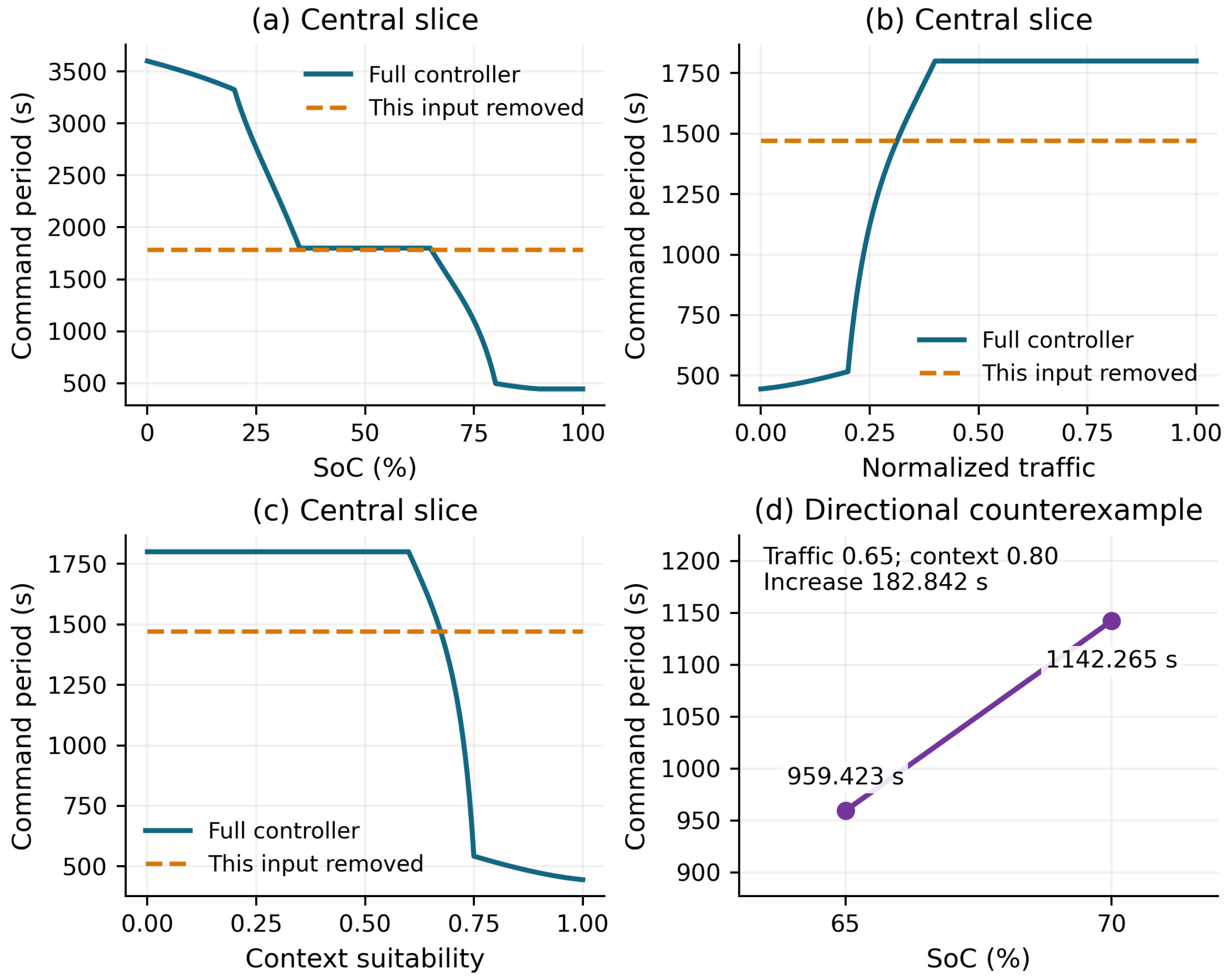

Figure S2. Central command-period slices for (a) SoC, (b) traffic, and (c) context, with other inputs fixed at 50%, 0.5, and 0.5 as applicable. Dashed curves remove the plotted input. Panel (d) retains the SoC-direction counterexample.

S2.2 Operating-point commands and finite differences

Representative operating points reveal local behavior that a single central slice cannot characterize. Table S5 separates the raw centroid, Low-SoC envelope, and quantized command at five input triples. At SoC 20%, traffic 0.2, and context 0.2, the envelope raises the raw 1800 s period to 2571.428571 s before downward quantization. At the central point, all three reported stages equal 1800 s. Equal commands at different operating points therefore need not imply equal sensitivity to neighboring inputs.

Table S6 evaluates quantized-command differences using fixed perturbations. All three central-point sensitivities are zero, whereas traffic sensitivity is positive at the other listed points. Around the SoC counterexample, the SoC response changes from +40.475 to −4.765 s per percentage point between the 65% and 70% operating points. Context sensitivity is −705 s per normalized unit at the former point and zero at the latter. These signed and zero responses qualify the main manuscript's qualitative decision-surface interpretation. They describe the stated perturbation scale, not analytical derivatives or universal directional ordering. Quantization and local plateaus must therefore remain part of their interpretation.

Table S5. Raw, enveloped, and quantized periods in seconds at five representative SoC, traffic, and context combinations.

Table S5					
**SoC percent**	**Traffic**	**Context**	**Raw s**	**Envelope s**	**Command s**
20	0.2	0.2	1800.000000	2571.428571	2571.428
50	0.5	0.5	1800.000000	1800.000000	1800.000
80	0.8	0.8	1800.000000	1800.000000	1800.000
65	0.65	0.8	959.423490	959.423490	959.423
70	0.65	0.8	1142.265134	1142.265134	1142.265

Table S6. Step-dependent command sensitivities using ±1 SoC percentage point and ±0.01 normalized traffic or context. Signed values retain the stated input-specific units.

Table S6			
**SoC traffic context**	**SoC s per percentage point**	**Traffic s per unit**	**Context s per unit**
20 / 0.2 / 0.2	-51.428500	1907.600000	0.000000
50 / 0.5 / 0.5	0.000000	0.000000	0.000000
80 / 0.8 / 0.8	0.000500	2895.750000	0.000000
65 / 0.65 / 0.8	40.475000	6416.050000	-705.000000
70 / 0.65 / 0.8	-4.765000	5807.150000	0.000000

S2.3 Quantization and exact-input comparisons

Nominal 1 ms downward quantization does not remove every distinction between floating-point evaluation and exact input identity. The adjacent-grid diagnostic separately identifies 126 additional SoC, 77 traffic, and 75 context reversals of 1 ms. They arise when sub-tolerance differences on numerical plateaus fall on opposite sides of a floor boundary. The preregistered comparison tolerance remains 10^{-8} s. These boundary effects accompany larger directional counterexamples and do not justify changing the reported counts or claiming monotonicity.

The grid and dense-slice records agree for 228 commands evaluated at exactly matching stored input coordinates. Six additional nominally matching locations yield 24 comparisons across four controllers: 23 agree and one differs by 1 ms. At SoC 50% and traffic 0.5, context stored as 0.35 gives 1800 s, whereas 0.35000000000000003 gives 1799.999 s. Identical printed coordinate labels therefore do not guarantee identical numerical inputs.

At common inputs, the envelope produces zero shortenings among 9261 points and 3087 strict lengthenings, with a maximum lengthening of 1800.001 s. One-quantum effects occur near exact millisecond boundaries. Separately, campaign command-lattice residuals reach 3×10^{-13} s. Rational timestamps preserve recorded periods without recovering precision lost in binary64 arithmetic. Their outcome effect remains unestablished. These distinct checks delimit the manuscript's quantization specification without inferring outcome equivalence from small numerical discrepancies.

S3 Ablation Study

S3.1 Structural input removal and static effect magnitudes

Structural removal tests how each input changes the declared controller, using a predetermined rule transformation rather than a fixed replacement value. Its antecedent is deleted from every rule. For each retained two-input combination, every distinct consequent remains active with weight $c_D/3$, where c_D counts its occurrences across the three removed linguistic categories. Removing SoC also disables the Low-SoC envelope. The shared delivered-telemetry readiness and 7200 s age gate nevertheless remain, so this intervention does not remove every protocol dependence on SoC.

Table S7 reports reduced-minus-full command differences. Mean absolute changes are 685.810064 s for SoC removal, 341.244532 s for traffic removal, and 327.398243 s for context removal. Signed means distinguish overall direction from cancellation across operating conditions: SoC removal decreases the grid mean, whereas the other removals increase it. The changed-point counts are 8871, 8245, and 7660, respectively. Each reduced controller has 441 unique retained-input contexts, repeated across the deleted coordinate only to align comparisons with the full 9261-point grid. These are deterministic command effects, not independent replications or energy measurements. They make the intervention in the main manuscript's ablation method explicit while limiting attribution to this particular structural projection; they do not establish universal input necessity.

Table S7. Structural-removal differences from the full controller in seconds: signed mean, mean absolute change, maximum absolute change, and changed aligned points. No sampling intervals are assigned.

Table S7				
**Removed input**	**Mean signed s**	**Mean absolute s**	**Maximum absolute s**	**Different points**
soc	-186.430020	685.810064	2560.644	8871
traffic	38.010774	341.244532	1076.924	8245
weather	39.915057	327.398243	1076.924	7660

S3.2 Reduced-controller command ranges

The structural reductions described in Section **Hata! Başvuru kaynağı bulunamadı.** alter the range of available commands as well as their pointwise differences. Table S8 compares minima, maxima, and arithmetic grid means at the same 9261 aligned locations. The full controller reaches 445.104 s, whereas all three reductions have a minimum of 1039.356 s. The SoC-removed controller also has a lower

maximum, 2563.636 s, while the full, traffic-removed, and context-removed controllers each reach 3600 s. Thus, removing SoC narrows the sampled command range at both ends.

The full grid mean is 1933.390099 s. The SoC-removed mean is 1746.960079 s, despite its higher minimum; the traffic-removed and context-removed means are 1971.400873 and 1973.305156 s. A higher minimum therefore does not determine the direction of the overall mean change. This distributional distinction complements the main manuscript's input-removal rationale without repeating the pointwise attribution analysis. Each reduced controller contains 441 distinct retained-input contexts, with equal repetition along the absent coordinate for alignment. These grid means summarize that deterministic domain weighting; they are not expected reporting periods under a deployment's state distribution. No sampling intervals, energy benefit, delivered-service improvement, or lifetime extension follow from these range summaries alone.

Table S8. Minimum, maximum, and mean commanded periods in seconds for the full controller and three structural reductions. All evaluated variants retain the same aligned grid weighting.

Table S8			
**Variant**	**Minimum s**	**Maximum s**	**Mean s**
full	445.104	3600.000	1933.390099
minus_soc	1039.356	2563.636	1746.960079
minus_traffic	1039.356	3600.000	1971.400873
minus_weather	1039.356	3600.000	1973.305156

S3.3 Mixed-workload ablation energy and delivery

Input removal does not produce a common energy–service improvement across the two resource profiles. Table S9 compares each reduction with full arm D within eight matched exogenous seeds under the 72 h mixed workload. All six descriptive node-load intervals include zero. Traffic removal increases delivery by 2.475 percentage points at 1× and 7.416 points at 8×; context removal decreases it by 3.632 and 9.965 points. SoC removal increases delivery at 1× but decreases it at 8×.

Figure S3 places these paired effects alongside absolute energy and delivered-event outcomes. The directions of the command-period changes also depend on the removed input and profile. All variants retain minimum estimated total-energy selection and the common delivered-SoC gate. Their queues, energy states, information, and completed work evolve separately despite matched external inputs. Consequently, a lower load total cannot establish greater efficiency at equal service. These results extend the structural-ablation method in the main manuscript and restrict claims that every input necessarily improves both outcomes.

Table S9. Within-seed ablation-minus-D contrasts: node load in joules over 72 h, delivery in percentage points, and mean command period in seconds. Brackets are descriptive, unadjusted 95% t intervals with seven degrees of freedom.

Table S9			
**Profile / removed input**	**Node-load change (J)**	**Delivery change (points)**	**Mean-command change (s)**
1× / soc	0.127 [-0.049, 0.303]	2.333 [0.408, 4.257]	-440.216 [-469.031, -411.401]
1× / traffic	0.116 [-0.057, 0.290]	2.475 [0.375, 4.574]	-147.732 [-225.969, -69.494]
1× / weather	0.386 [-0.018, 0.790]	-3.632 [-6.074, -1.189]	210.232 [119.391, 301.073]
8× / soc	-71.577 [-293.289, 150.135]	-11.154 [-12.527, -9.782]	340.267 [330.651, 349.882]
8× / traffic	-70.169 [-225.993, 85.655]	7.416 [5.579, 9.252]	-203.474 [-218.743, -188.204]
8× / weather	29.614 [-125.744, 184.973]	-9.965 [-11.675, -8.256]	246.943 [234.716, 259.170]

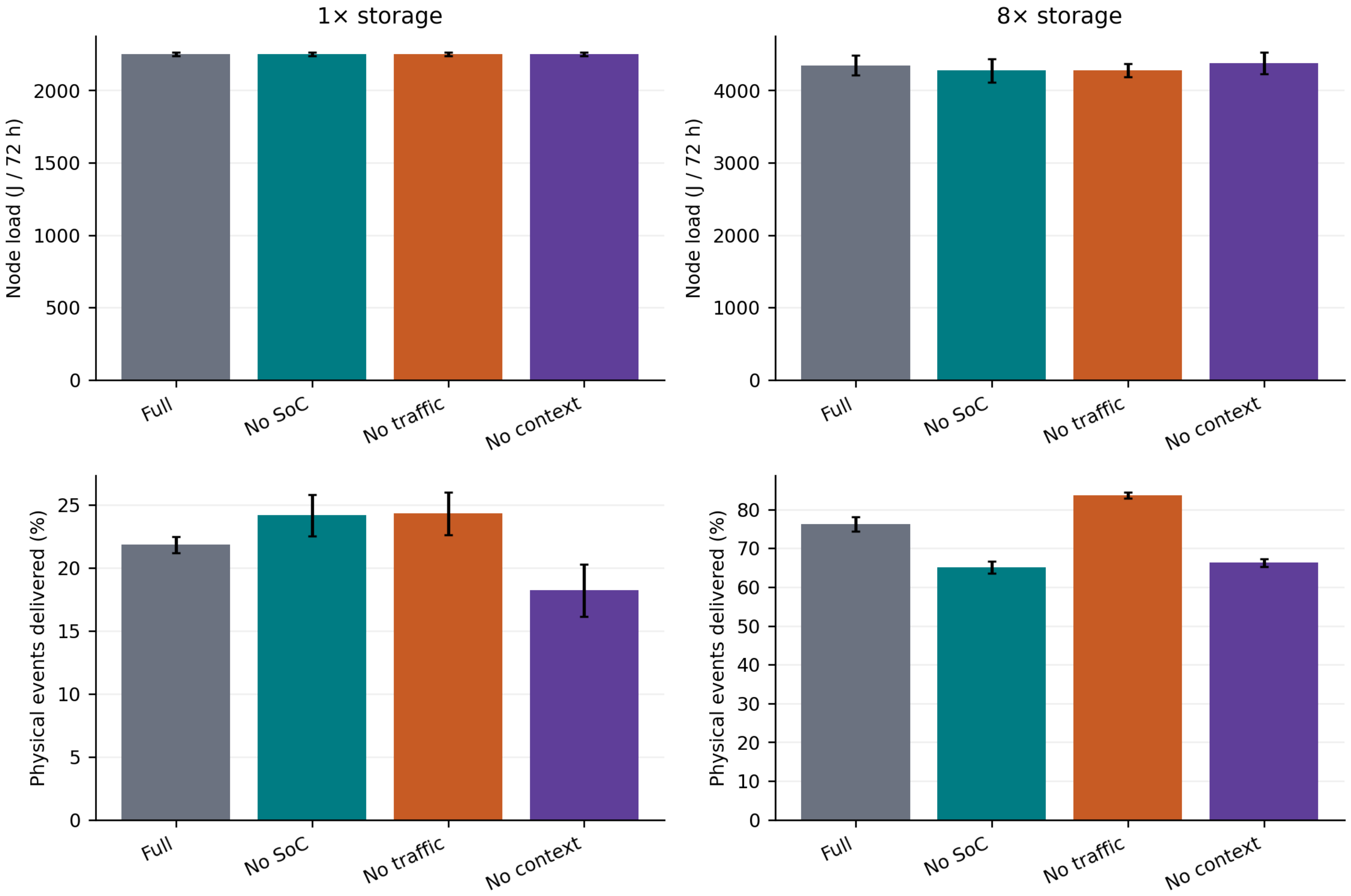

Figure S3. Mixed-workload absolute node load (top) and physical-event delivery (bottom), for 1× (left) and 8× (right). Bars show means and 95% t intervals across eight seeds. “No context” denotes D_no_weather.

S3.4 Absolute outcomes under input removal

Absolute outcomes place the paired contrasts in Section **Hata! Başvuru kaynağı bulunamadı.** on their original measurement scales. Table S10 retains full arm D and each structural input removal at both resource profiles. At 1×, full D consumes 2249.86 J and delivers 21.83% of physical events; removing traffic gives 2249.98 J and 24.31% delivery. Removing context instead gives 2250.25 J and 18.20% delivery. These closely spaced energy means accompany distinguishable delivery patterns rather than a common benefit of retaining every input.

At 8×, full D consumes 4346.44 J and delivers 76.25% of events. The SoC-removed controller has a lower mean load, 4274.87 J, but also lower delivery, 65.09%. Traffic removal combines 4276.27 J with 83.66% delivery, whereas context removal combines 4376.06 J with 66.28%. The command-period column adds a different outcome: it averages defined per-run means of generated commands, including commands that may remain unapplied. It therefore describes gateway proposals rather than average sleep residence or guaranteed node reporting behavior. These absolute summaries extend the main manuscript's input-removal evaluation while preserving the service dependence of energy interpretation. Within-seed contrasts remain necessary for paired effects; subtracting the displayed interval endpoints would not produce a valid paired interval.

Table S10. Absolute mixed-workload outcomes for full D and three structural removals at both profiles over 72 h. Energy and delivery show eight-seed means with descriptive 95% t intervals; command values average defined per-run mean generated periods.

Table S10			
**Profile / variant**	**Node load (J)**	**Mean command (s)**	**Physical events delivered (%)**
1× / D	2249.86 [2236.83, 2262.89]	2382.10	21.83 [21.19, 22.48]
1× / no soc	2249.99 [2236.94, 2263.04]	1941.88	24.17 [22.51, 25.82]
1× / no traffic	2249.98 [2236.83, 2263.13]	2234.36	24.31 [22.62, 26.00]
1× / no weather	2250.25 [2237.42, 2263.08]	2592.33	18.20 [16.14, 20.27]
8× / D	4346.44 [4210.34, 4482.54]	1501.51	76.25 [74.40, 78.10]
8× / no soc	4274.87 [4114.33, 4435.40]	1841.77	65.09 [63.56, 66.63]
8× / no traffic	4276.27 [4181.92, 4370.63]	1298.03	83.66 [82.85, 84.48]
8× / no weather	4376.06 [4226.64, 4525.48]	1748.45	66.28 [65.31, 67.26]

S3.5 Historical structural input removal

The historical 216-run campaign tested structural input removal during the mixed workload, independently of the current 432-run evaluation. Removing an input deletes its antecedent and

resolves the remaining consequent conflicts using predeclared relative-frequency weights. Removing SoC also removes its envelope dependence. Every variant nevertheless retains the common delivered-telemetry readiness gate: missing telemetry or an age exceeding 7200 s suppresses command creation.

Table S11 shows closely spaced node-load means but different command and delivery outcomes. Full control consumes 2249.87 J, generates a mean command of 2380.41 s, and delivers 21.69% of physical events. Traffic removal gives 2249.96 J, 2228.24 s, and 24.44%; context removal gives 2250.03 J, 2537.06 s, and 19.03%. SoC removal shortens the mean command to 1949.26 s with 23.94% delivery. Figure S4 places energy and delivery together. These mixed outcomes support scenario-specific contributions rather than universal necessity of every input. They preserve the historical basis of the manuscript's ablation discussion without substituting for successor results. The eight matched seeds support descriptive uncertainty; cross-policy energy differences also reflect different realized work.

Table S11. Historical mixed-workload structural removals: node load, generated command period, and physical delivery; means and descriptive 95% t intervals across eight seeds.

Table S11			
**Variant**	**Node load J**	**Mean command s**	**Events delivered %**
Full	2249.87 [2236.82, 2262.92]	2380.41 [2341.29, 2419.54]	21.69 [21.08, 22.30]
No SoC	2250.04 [2236.99, 2263.10]	1949.26 [1919.49, 1979.04]	23.94 [21.46, 26.41]
No traffic	2249.96 [2236.87, 2263.04]	2228.24 [2139.83, 2316.66]	24.44 [22.30, 26.58]
No weather	2250.03 [2237.07, 2262.98]	2537.06 [2459.49, 2614.63]	19.03 [16.47, 21.58]

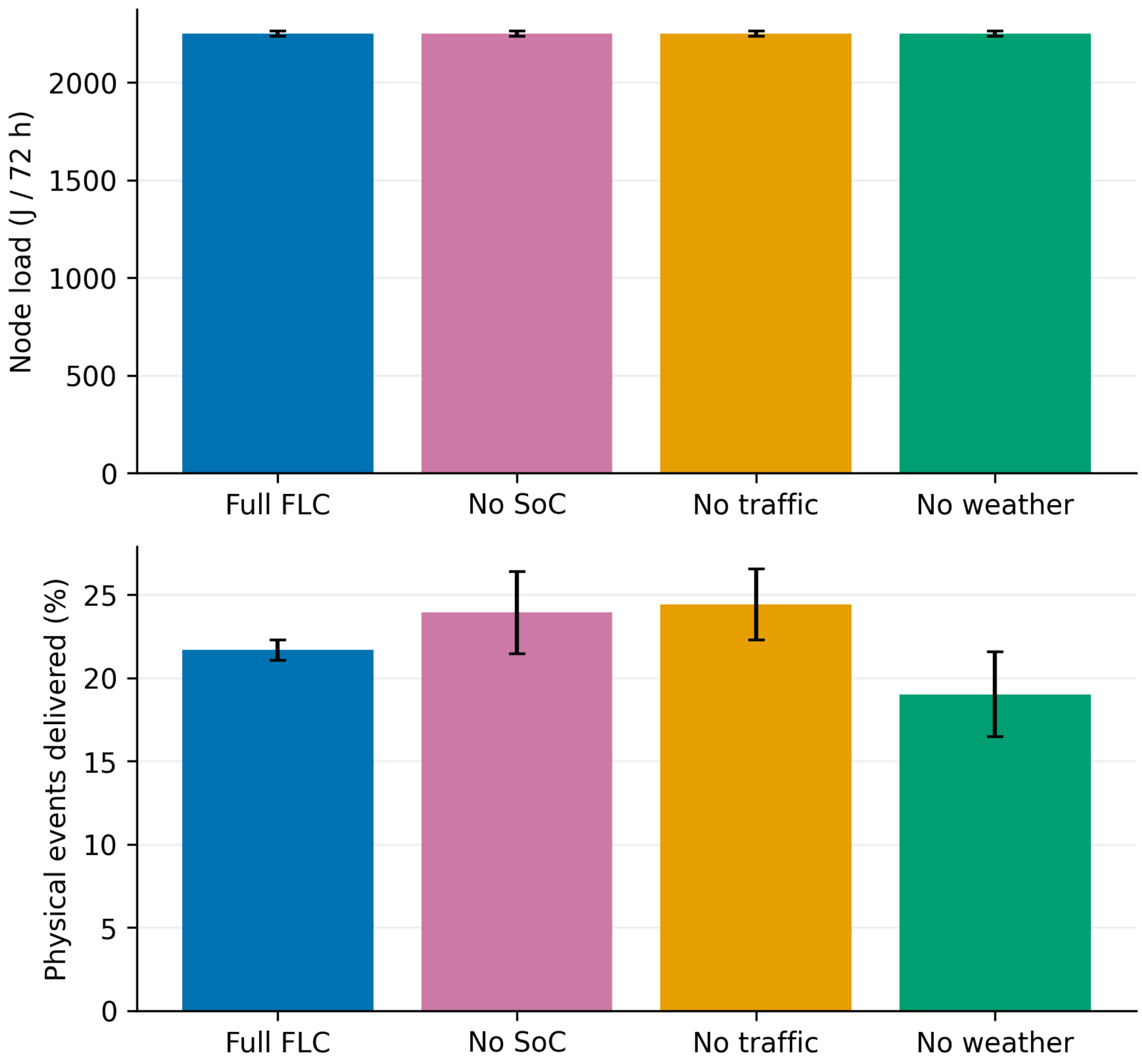

Figure S4. Historical input-removal energy and delivery, shown jointly in two panels with eight-seed means and descriptive 95% t intervals.

S3.6 Midpoint input clamps in the 24 h model

The separate 24 h network model tests constant-input clamps rather than structural rule deletion. SoC is fixed at 50%, received-load input at 0.5, or weather context at 0.5, one channel at a time. Physical harvesting remains enabled when weather information is clamped. Each comparison uses 30 independent traces of 100 nodes with the same exogenous conditions within a pair.

In Figure S5, the respective clamp configurations consume 1082.032, 1077.661, and 1006.040 J/node while delivering 38.415%, 38.409%, and 37.806% of physical events. Full control consumes 1040.718 J/node with 37.700% delivery. Retaining each varying input therefore does not improve both measured outcomes. Figure S6 separates effective intervals weighted by node time from means conditional on actual application events. Initial defaults and cache fallback contribute to the former; the latter has a different observation denominator. Neither is an average over an invented continuous gateway-output history. These historical comparisons qualify the manuscript's input-contribution interpretation, while

preserving the distinction between controller information, physical environmental forcing, and realized service.

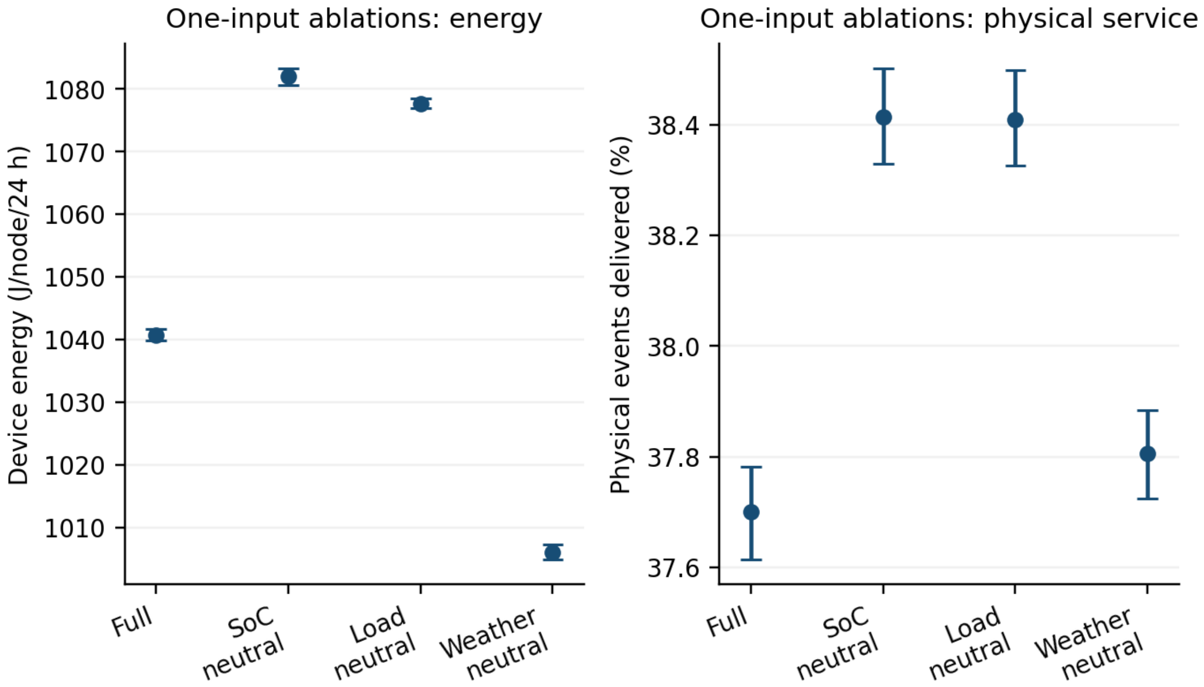

Figure S5. Energy and physical delivery under the three midpoint clamps; pointwise exploratory 95% intervals over 30 complete network traces.

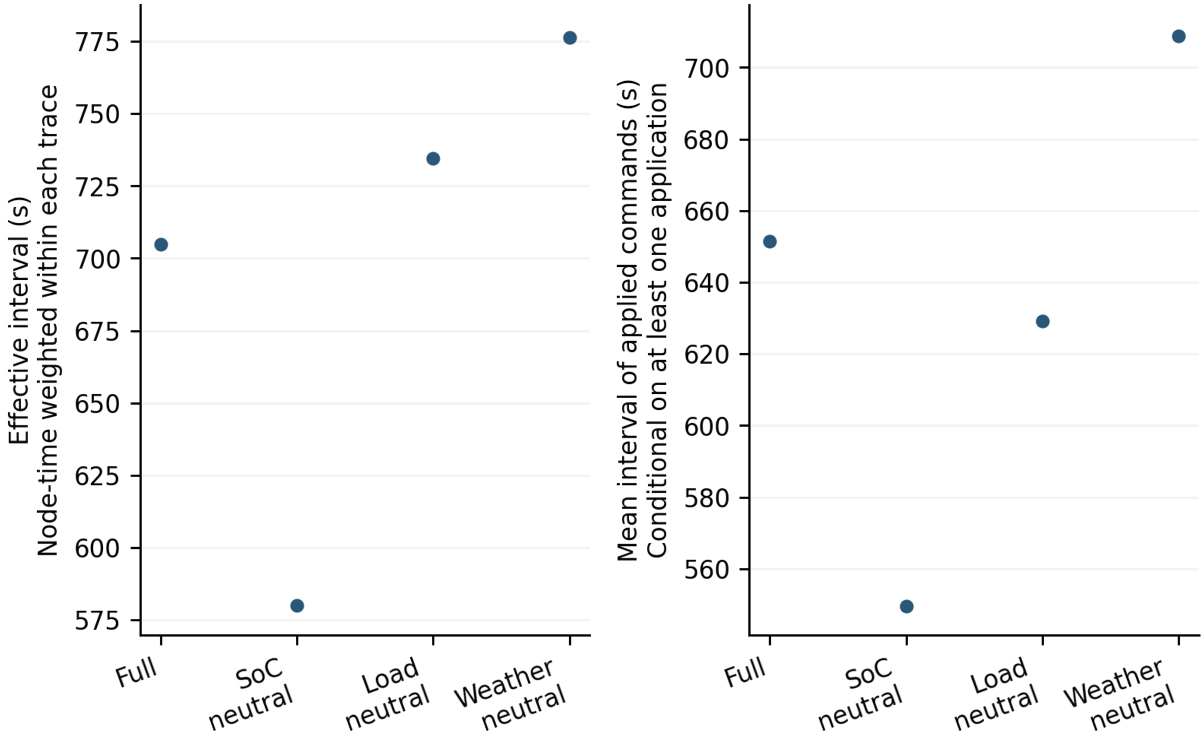

Figure S6. Effective node-time-weighted periods and conditional application-event means for the full controller and midpoint clamps in the 24 h model.

S3.7 Central-reference information neutralization

Constant-reference neutralization measures how much of the frozen policy surface changes when one information channel ceases to vary. The deterministic design contains 41 values per input, giving 68921 points. Each full and neutralized evaluation shares the other two coordinates. The central references are SoC 50%, traffic 0.50, and context 0.50; differences are neutralized minus full. This intervention preserves the inference structure and is distinct from the structural removals in Section **Hata! Başvuru kaynağı bulunamadı.**.

Table S12 retains signed and absolute effects on synchronization period, conditional daily energy, and equivalent synchronization frequency. SoC neutralization has the largest period mean absolute effect, 499.9 s, compared with 273.3 s for traffic and 269.7 s for context. The corresponding absolute energy effects are 72.2, 64.6, and 67.7 J/day. Every evaluated idle-mode change fraction is zero. SoC attribution includes its complete envelope pathway rather than the rule base alone. Although central-reference mean periods decrease and conditional energy increases, these signed directions reverse across alternative references. This historical analysis supports preserving the manuscript's specified policy surface; it does not establish that every channel improves a service objective or that a redesigned two-input controller would fail.

Table S12. Central-reference neutralization effects on periods, conditional energy, synchronization frequency, and mode selection over the deterministic 41^3 domain; signed differences use neutralized minus full.

Table S12							
**Neutralized input**	**Mean Delta T_sync (s)**	**MAE T_sync (s)**	**Mean Delta E (J/day)**	**MAE E (J/day)**	**Mean Delta events/day**	**MAE events/day**	**Idle-mode change**
Supercapacitor SoC	-331.2	499.9	+40.6	72.2	+11.7	20.9	0.0%
Traffic load	-109.7	273.3	+32.0	64.6	+9.3	18.7	0.0%
Weather/control context	-99.4	269.7	+27.3	67.7	+7.9	19.6	0.0%

S3.8 Reference robustness and conditional input influence

Input influence varies across reference choices and operating contexts. Table S13 compares SoC references of 25%, 50%, and 75%, and traffic/context references of 0.25, 0.50, and 0.75. Period mean absolute effects range from 499.9 to 831.5 s for SoC, 273.3 to 400.5 s for traffic, and 269.7 to 452.4 s for context. Figure S7 combines these reference ranges with first- and total-order indices.

The reference-independent spans in Figure S8 exceed 1 s in 100.0%, 91.7%, and 88.5% of the 1681 fixed two-input contexts, respectively. Interval total-order indices are 0.708, 0.219, and 0.224 under equal discrete-uniform grid weighting. Thus, SoC has the largest design-weighted interval influence, while traffic and context retain substantial but spatially uneven effects. Zero-span regions indicate local insensitivity, not universal redundancy. This historical evidence refines the manuscript's input

interpretation without treating reference ranges as confidence intervals or grid weights as an empirical deployment distribution.

Table S13. Reference choices, absolute-effect ranges, conditional spans, and total-order indices for the deterministic neutralization domain.

Table S13

Input	Neutral references	MAE T_sync range (s)	MAE E range (J/day)	Mean T_sync span (s)	Contexts with span > 1 s	T total-order	E total-order
Supercapacitor SoC	25/50/75%	499.9-831.5	72.2-118.6	1983.6	100.0%	0.708	0.537
Traffic load	0.25/0.50/0.75	273.3-400.5	64.6-89.9	899.2	91.7%	0.219	0.439
Weather/control context	0.25/0.50/0.75	269.7-452.4	67.7-95.4	830.8	88.5%	0.224	0.465

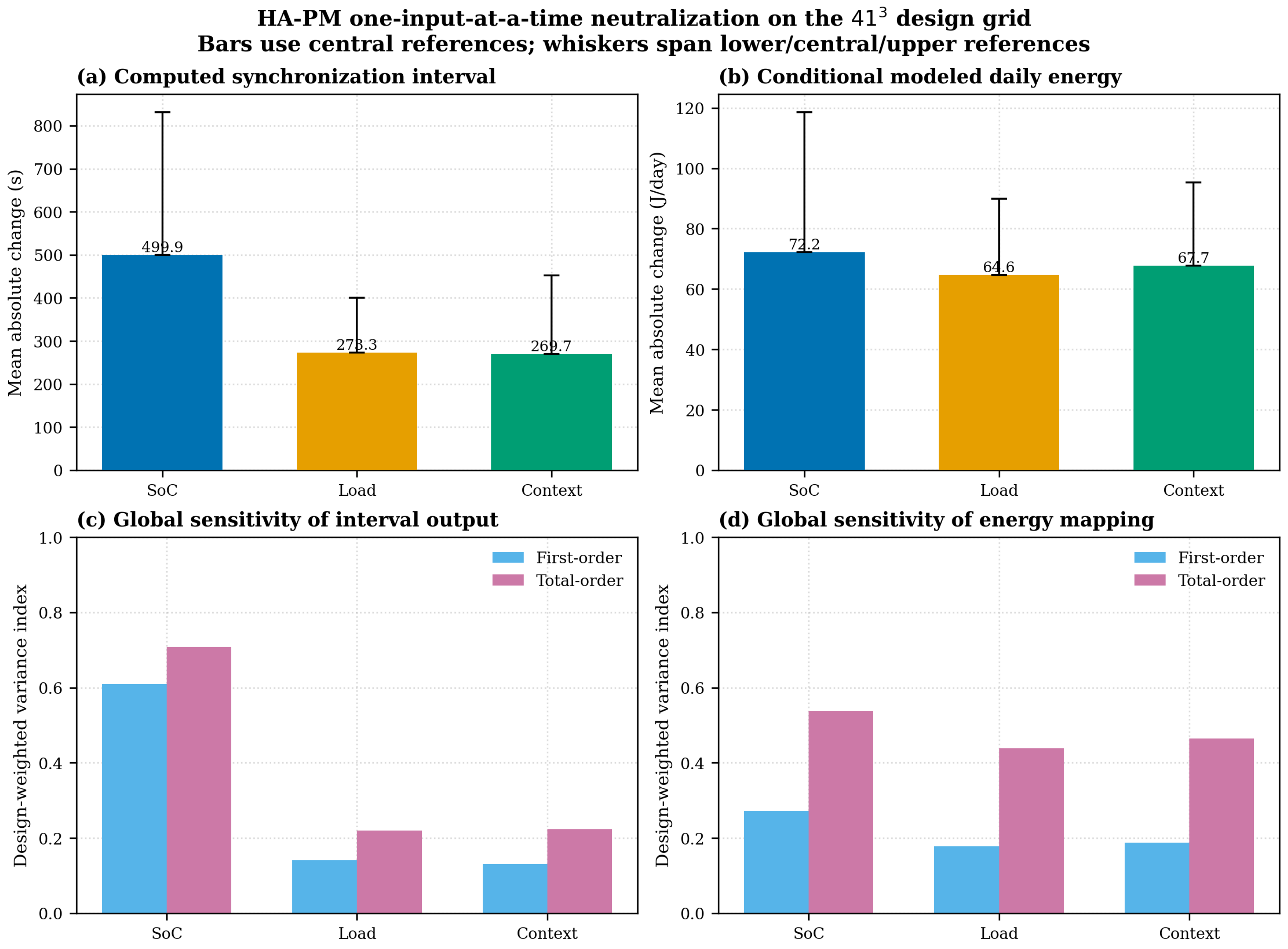

Figure S7. Four panels compare interval and conditional-energy absolute effects and their first- and total-order sensitivity indices.

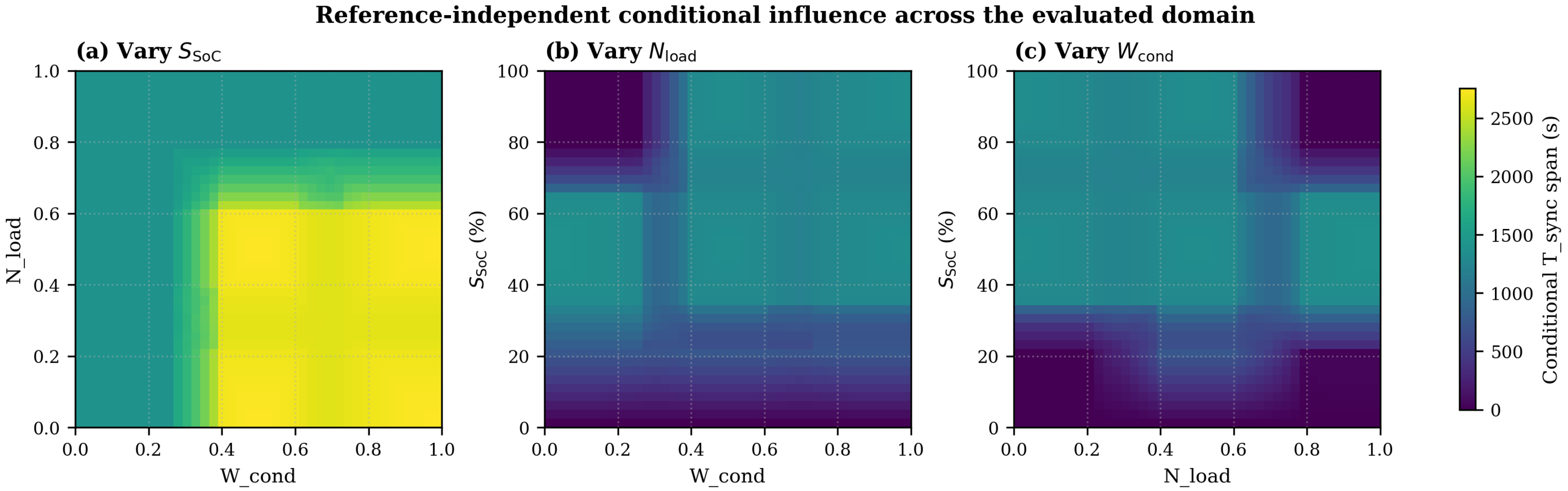

Figure S8. Conditional period-span maps when varying SoC, traffic, or context while fixing the other two inputs.

S3.9 Conditional energy under invariant M19 selection

The historical neutralization study separates changes in synchronization cadence from changes in selected idle mode. For each computed period, the selector subtracts a fixed 2 s active residence and applies the thresholds in Table S14. These thresholds are 7, 15, 30, 189, and 300 seconds in this model. Their units differ from the millisecond policy of the event-service evaluations and cannot be interchanged.

Across the full and neutralized mappings, periods range from 445.104 to 3200.000 s. The minimum inactive duration is consequently 443.104 s, exceeding the 300 s threshold; every evaluated configuration selects M19. This is a consequence of the sampled output range, rather than an unconditional instruction to use M19. Under fixed M0 residence and the declared M19 idle power, the displayed conditional daily-energy equation applies. Its constant term represents uninterrupted M19 power over a day, while its reciprocal-period term represents the additional active-residence contribution. Lower conditional energy therefore accompanies fewer synchronization cycles. This mechanism clarifies the historical energy interpretation associated with the manuscript, but it does not establish service-equivalent efficiency, realized event delivery, or energy consumption in the current 432-run model.

Table S14. Historical selector thresholds in seconds, applied to the inactive duration after subtracting the fixed 2 s active residence.

Table S14	
**Inactive duration**	**Selected mode**
< 7 s	M0
7 to < 15 s	M15
15 to < 30 s	M16
30 to < 189 s	M17
189 to < 300 s	M18
>= 300 s	M19

$$E\_day(T) = 60.48 + 298823.04 / T \quad J/day$$

S4 Current Evaluation Protocol

S4.1 Policy factors and matched exogenous traces

The current evaluation crosses two gateway policies with two node selectors. Table S15 defines the four factorial arms and the three mixed-workload input removals. The selector comparison includes a change from an EARLIEST-only catalogue to EARLIEST/LATEST candidates; it is therefore a comparison of selector packages.

Within each scenario and seed, event arrivals, context, harvesting, temperature, and keyed channel inputs are shared. Energy, queues, sensing success, delivery, and gateway knowledge evolve

independently in each arm. Matched external inputs consequently support within-seed comparisons without imposing identical completed work. This design makes the main manuscript's simulation factors explicit and distinguishes gateway-policy effects on node energy from the gateway's own consumption. The additional removal variants retain the declared selector and common telemetry-readiness gate.

Table S15. Internal policy definitions. A–D cross reporting policy and selector; the three additional variants remove one FLC input under mixed traffic. All rows retain their stated intervention scope.

Table S15	
**Arm**	**Definition**
A	Fixed 900 s nominal period; lowest-residence-power feasible EARLIEST baseline.
B	Fixed 900 s nominal period; minimum estimated total energy selector.
C	Full FLC nominal-period controller; lowest-residence-power feasible EARLIEST baseline.
D	Full FLC nominal-period controller; minimum estimated total energy selector.
D_no_soc	Remove the SoC antecedent and low-energy envelope; minimum estimated total energy selector.
D_no_traffic	Remove the traffic antecedent; minimum estimated total energy selector.
D_no_weather	Remove the weather/context antecedent; minimum estimated total energy selector.

S4.2 Scenario design and the completed cohort

The current cohort comprises 54 cells with eight seeds each, giving 432 simulations of 72 h. At each of two resource profiles, six scenarios cross four arms, and three input removals are added under mixed traffic. Table S16 preserves the scenario definitions supporting the main manuscript's evaluation.

The 8× profile scales capacitance, stored-energy thresholds, and reference leakage together; the absolute reserve remains 5 J. It is a declared resource configuration, not measured hardware. The 216 runs within either profile are distinct from the historical 216-run campaign. Store provenance comprises 55 preserved and 377 successor records, whereas code lineage comprises 41 original-optimized and 391 memory-execution records. These are separate classifications, not additional runs or interchangeable populations.

Table S16. The six scenario definitions applied to the current 72 h campaign. Each profile includes eight matched seeds per four-arm scenario cell and three additional mixed-workload removal cells.

Table S16	
**Scenario**	**Definition**
nominal	72 h with nominal events and harvest; temperature 15–35 °C.

Table S16	
**Scenario**	**Definition**
mixed	Alternate low and high Poisson event rates every 12 h; simultaneous three-event bursts at 6 + 12k hours.
outage	Gateway unavailable from hour 12 through hour 48. Sensing and radio listening remain scheduled; actual radio activity and leakage are accounted, and recovery is measured.
hot	Nominal conditions with temperature increased by 20 °C, giving 35–55 °C.
high_leak	Nominal conditions with a tenfold leakage-current multiplier.
no_harvest	Zero realized harvest; temperature 15–35 °C.

S5 Energy and Service Outcomes

S5.1 Energy and service across the four arms

Energy and service vary together across the four policy arms. Figure S9 separates node load, completed sensing, and deadline-based service completion for each profile. The limited 1× resource configuration exhibits substantial service loss, so its lower energy totals cannot be read as efficient execution of the larger profile's workload. Sensing completion, due-service completion, and physical-event delivery have different denominators and remain separate outcomes.

Table S17 details the mixed workload. At 1×, D delivers 21.83% of physical events compared with A's 59.30%, despite similar node-load means. At 8×, D consumes 4346.44 J and delivers 76.25%, whereas A consumes 4128.17 J and delivers 91.79%. Full adaptation therefore does not improve both measures in this comparison. Due-service percentages near 100% also require the separate all-due-success counts. These results extend the main manuscript's 72 h evaluation with scenario-specific evidence rather than an unconditional energy advantage. Uncertainty remains seed-based and descriptive; intervals are retained without clipping their percentage endpoints.

Table S17. Mixed-workload A–D means at both profiles: node load, physical-event delivery, and due-service completion. Brackets are descriptive 95% t intervals over eight seeds.

Table S17			
**Profile / arm**	**Node load (J / 72 h)**	**Physical events delivered (%)**	**Due services completed (%)**
1× / A	2249.78 [2236.75, 2262.82]	59.30 [58.31, 60.29]	53.83 [53.49, 54.16]
1× / B	2249.78 [2236.75, 2262.82]	59.30 [58.31, 60.29]	53.87 [53.53, 54.20]
1× / C	2249.87 [2236.85, 2262.90]	22.10 [21.59, 22.62]	52.45 [51.73, 53.17]
1× / D	2249.86 [2236.83, 2262.89]	21.83 [21.19, 22.48]	52.21 [51.47, 52.95]

Table S17			
**Profile / arm**	**Node load (J / 72 h)**	**Physical events delivered (%)**	**Due services completed (%)**
8× / A	4128.17 [4128.15, 4128.20]	91.79 [90.86, 92.72]	100.00 [100.00, 100.00]
8× / B	4128.17 [4128.15, 4128.20]	91.79 [90.86, 92.72]	100.00 [100.00, 100.00]
8× / C	4565.27 [3918.63, 5211.92]	74.86 [72.36, 77.36]	99.97 [99.94, 100.00]
8× / D	4346.44 [4210.34, 4482.54]	76.25 [74.40, 78.10]	99.99 [99.98, 100.00]

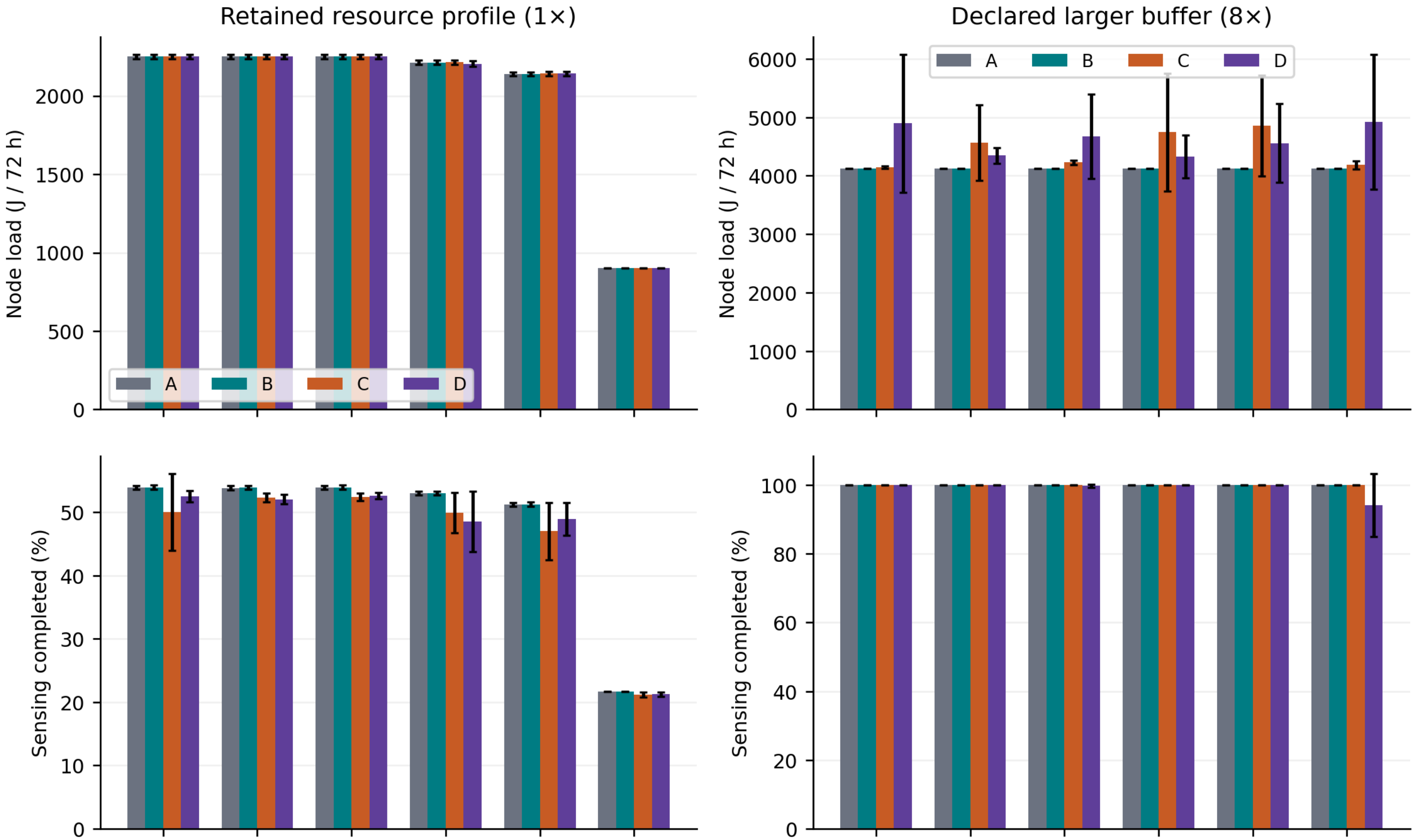
Retained resource profile (1×)
Declared larger buffer (8×)
Node load (J / 72 h)
Sensing completed (%)
A
B
C
D
0
500
1000
1500
2000
0
1000
2000
3000
4000
5000
6000
0
10
20
30
40
50
0
20
40
60
80
100

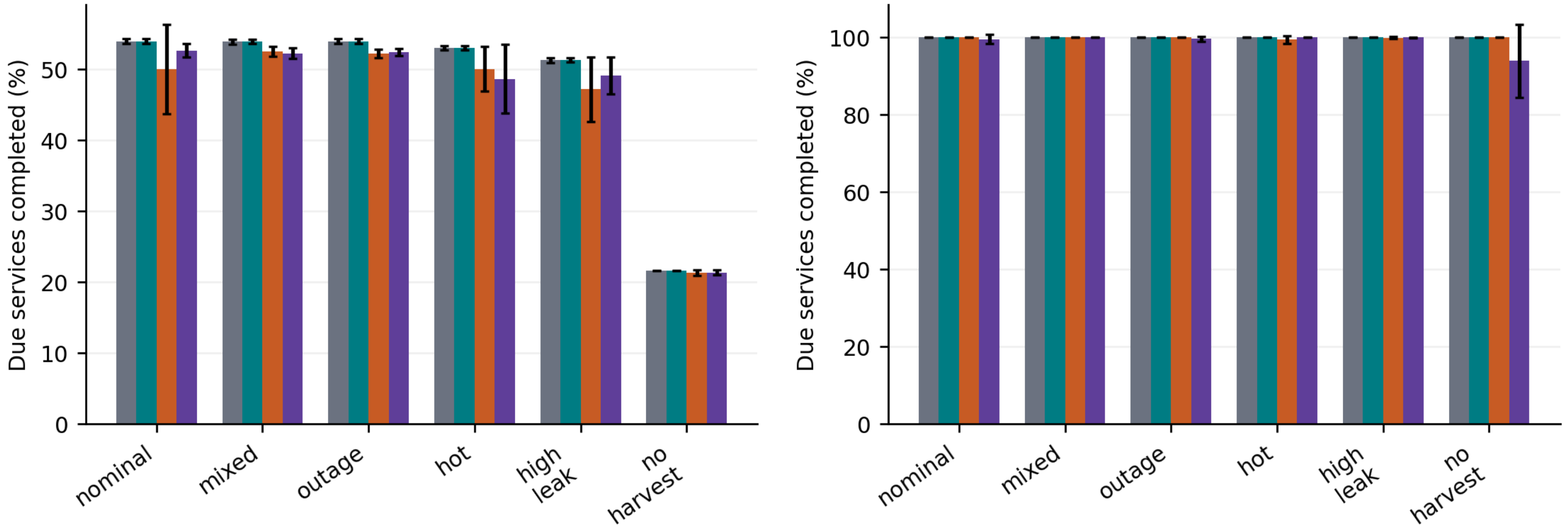

Figure S9. Four-arm means and 95% t intervals: node load (top), sensing completion (middle), and due-service completion (bottom); 1× left and 8× right. All six scenarios and unclipped percentage intervals are retained.

S5.2 Complete retained-profile outcome census

The complete 1× census in Table S18 shows a resource constraint shared across the evaluated policy combinations. All 216 runs experience a first brownout, and none completes every due service on time. These totals span 24 four-arm scenario cells and three mixed-workload removal cells. They establish what occurred in this design, rather than a single population failure probability.

The detailed rows also show why brownout and service percentages cannot replace each other. Nominal arm A completes 53.88% of due services while delivering 52.69% of physical events; under outage, its due-service completion remains 53.88% while event delivery falls to 25.64%. Under no harvesting, arm D's mean node load is 903.27 J, with 21.32% due-service completion and 19.69% event delivery. The low expenditure therefore accompanies extensive lost service. The mixed-workload removal rows remain part of the same census, including adverse context-removal outcomes. These data delimit the main manuscript's operational-continuity interpretation over 72 h. Each row summarizes eight seeds with its own condition and uncertainty; deterministic or rounded interval endpoints do not establish physical-parameter certainty, and the table does not support lifetime extrapolation beyond the observation horizon.

Table S18. Complete 1× census: 27 cells, eight seeds per cell, and 72 h per run. Brackets are descriptive 95% t intervals; brownout and all-due-success columns retain observed counts out of eight.

Table S18					
**Scenario / arm**	**Node load (J)**	**Events delivered (%)**	**Due services (%)**	**Brownout runs**	**All due success**
nominal / A	2249.78 [2236.75, 2262.81]	52.69 [51.70, 53.69]	53.88 [53.54, 54.22]	8/8	0/8
nominal / B	2249.78 [2236.75, 2262.81]	52.72 [51.73, 53.72]	53.89 [53.54, 54.23]	8/8	0/8
nominal / C	2250.00 [2237.01, 2263.00]	47.33 [37.52, 57.13]	50.00 [43.72, 56.28]	8/8	0/8
nominal / D	2249.82 [2236.79, 2262.85]	51.27 [49.70, 52.83]	52.60 [51.67, 53.52]	8/8	0/8
mixed / A	2249.78 [2236.75, 2262.82]	59.30 [58.31, 60.29]	53.83 [53.49, 54.16]	8/8	0/8
mixed / B	2249.78 [2236.75, 2262.82]	59.30 [58.31, 60.29]	53.87 [53.53, 54.20]	8/8	0/8
mixed / C	2249.87 [2236.85, 2262.90]	22.10 [21.59, 22.62]	52.45 [51.73, 53.17]	8/8	0/8
mixed / D	2249.86 [2236.83, 2262.89]	21.83 [21.19, 22.48]	52.21 [51.47, 52.95]	8/8	0/8

Table S18					
**Scenario / arm**	**Node load (J)**	**Events delivered (%)**	**Due services (%)**	**Brownout runs**	**All due success**
mixed / D−soc	2249.99 [2236.94, 2263.04]	24.17 [22.51, 25.82]	50.71 [48.40, 53.01]	8/8	0/8
mixed / D−traffic	2249.98 [2236.83, 2263.13]	24.31 [22.62, 26.00]	51.33 [49.85, 52.82]	8/8	0/8
mixed / D−weather	2250.25 [2237.42, 2263.08]	18.20 [16.14, 20.27]	48.97 [45.22, 52.73]	8/8	0/8
outage / A	2249.78 [2236.75, 2262.81]	25.64 [23.99, 27.29]	53.88 [53.54, 54.22]	8/8	0/8
outage / B	2249.78 [2236.75, 2262.81]	25.64 [23.99, 27.29]	53.89 [53.54, 54.23]	8/8	0/8
outage / C	2249.87 [2236.86, 2262.89]	24.72 [23.16, 26.28]	52.20 [51.59, 52.80]	8/8	0/8
outage / D	2249.85 [2236.81, 2262.89]	24.82 [22.96, 26.69]	52.38 [51.91, 52.86]	8/8	0/8
hot / A	2212.78 [2199.78, 2225.78]	51.80 [50.86, 52.75]	52.97 [52.65, 53.30]	8/8	0/8
hot / B	2212.78 [2199.78, 2225.78]	51.80 [50.86, 52.75]	52.99 [52.68, 53.30]	8/8	0/8
hot / C	2213.26 [2200.30, 2226.21]	48.31 [45.55, 51.08]	50.00 [46.83, 53.18]	8/8	0/8
hot / D	2204.90 [2185.03, 2224.76]	46.55 [41.17, 51.93]	48.57 [43.72, 53.42]	8/8	0/8
high leak / A	2139.57 [2126.65, 2152.49]	50.07 [49.00, 51.15]	51.22 [50.90, 51.54]	8/8	0/8
high leak / B	2139.57 [2126.64, 2152.49]	50.07 [49.00, 51.15]	51.23 [50.91, 51.55]	8/8	0/8
high leak / C	2141.41 [2128.25, 2154.56]	45.77 [41.49, 50.04]	47.12 [42.59, 51.64]	8/8	0/8
high leak / D	2140.72 [2127.17, 2154.28]	47.48 [43.95, 51.02]	49.05 [46.45, 51.65]	8/8	0/8
no harvest / A	903.18 [903.18, 903.18]	19.99 [19.10, 20.88]	21.63 [21.63, 21.63]	8/8	0/8

Table S18					
**Scenario / arm**	**Node load (J)**	**Events delivered (%)**	**Due services (%)**	**Brownout runs**	**All due success**
no harvest / B	903.18 [903.18, 903.18]	19.99 [19.10, 20.88]	21.63 [21.63, 21.63]	8/8	0/8
no harvest / C	903.27 [903.19, 903.36]	19.54 [18.17, 20.90]	21.29 [20.89, 21.68]	8/8	0/8
no harvest / D	903.27 [903.19, 903.35]	19.69 [18.59, 20.79]	21.32 [20.98, 21.66]	8/8	0/8

S5.3 Complete larger-buffer outcome census

The larger-resource census in Table S19 changes the observed continuity pattern without guaranteeing complete service. Of 216 runs, 214 have no first brownout within 72 h; the two observed crossings occur in no-harvest arm D. Only 172 runs nevertheless satisfy every due deadline. The combined capacitance, threshold, and leakage scaling must remain part of this comparison with the 1× profile.

A rounded service percentage does not identify that stricter success event. For example, nominal arm C displays 100.00% due-service completion but only seven successful runs out of eight. Mixed arm D displays 99.99% with five successes out of eight. The no-harvest D cell retains its two crossings and four all-due successes alongside the complete energy and delivery intervals. Its first-brownout mean conditions on two observed events and receives no confidence interval under the declared rule. These distinctions qualify the main manuscript's continuity claims: right-censoring at 72 h indicates that no crossing was observed within that window, not a measured lifetime of 72 h or infinite survival. All 27 cells remain visible, including imprecise percentage intervals whose untruncated upper endpoints exceed 100% as a statistical approximation.

Table S19. Complete 8× census over 72 h, with eight seeds per cell. Energy and service show means and descriptive 95% t intervals; separate count columns distinguish threshold crossing from complete due-service success.

Table S19					
**Scenario / arm**	**Node load (J)**	**Events delivered (%)**	**Due services (%)**	**Brownout runs**	**All due success**
nominal / A	4125.50 [4125.46, 4125.54]	98.80 [98.23, 99.37]	100.00 [100.00, 100.00]	0/8	8/8
nominal / B	4125.50 [4125.46, 4125.54]	98.80 [98.23, 99.37]	100.00 [100.00, 100.00]	0/8	8/8
nominal / C	4145.84 [4125.97, 4165.72]	98.37 [97.67, 99.07]	100.00 [99.99, 100.00]	0/8	7/8

Table S19					
**Scenario / arm**	**Node load (J)**	**Events delivered (%)**	**Due services (%)**	**Brownout runs**	**All due success**
nominal / D	4895.21 [3716.54, 6073.88]	89.19 [67.58, 110.81]	99.48 [98.28, 100.69]	0/8	6/8
mixed / A	4128.17 [4128.15, 4128.20]	91.79 [90.86, 92.72]	100.00 [100.00, 100.00]	0/8	8/8
mixed / B	4128.17 [4128.15, 4128.20]	91.79 [90.86, 92.72]	100.00 [100.00, 100.00]	0/8	8/8
mixed / C	4565.27 [3918.63, 5211.92]	74.86 [72.36, 77.36]	99.97 [99.94, 100.00]	0/8	2/8
mixed / D	4346.44 [4210.34, 4482.54]	76.25 [74.40, 78.10]	99.99 [99.98, 100.00]	0/8	5/8
mixed / D−soc	4274.87 [4114.33, 4435.40]	65.09 [63.56, 66.63]	99.98 [99.96, 100.00]	0/8	4/8
mixed / D−traffic	4276.27 [4181.92, 4370.63]	83.66 [82.85, 84.48]	99.97 [99.95, 100.00]	0/8	3/8
mixed / D−weather	4376.06 [4226.64, 4525.48]	66.28 [65.31, 67.26]	99.99 [99.99, 100.00]	0/8	3/8
outage / A	4125.50 [4125.46, 4125.54]	47.32 [45.00, 49.65]	100.00 [100.00, 100.00]	0/8	8/8
outage / B	4125.50 [4125.46, 4125.54]	47.32 [45.00, 49.65]	100.00 [100.00, 100.00]	0/8	8/8
outage / C	4229.07 [4192.58, 4265.56]	46.74 [44.44, 49.04]	100.00 [99.99, 100.00]	0/8	7/8
outage / D	4671.87 [3947.65, 5396.10]	39.96 [29.27, 50.65]	99.62 [98.94, 100.29]	0/8	6/8
hot / A	4125.50 [4125.46, 4125.54]	98.80 [98.23, 99.37]	100.00 [100.00, 100.00]	0/8	8/8
hot / B	4125.50 [4125.46, 4125.54]	98.80 [98.23, 99.37]	100.00 [100.00, 100.00]	0/8	8/8
hot / C	4744.76 [3736.02, 5753.50]	88.18 [70.19, 106.17]	99.41 [98.38, 100.44]	0/8	5/8
hot / D	4330.71 [3961.71, 4699.71]	98.70 [98.16, 99.24]	100.00 [99.99, 100.00]	0/8	7/8

Table S19					
**Scenario / arm**	**Node load (J)**	**Events delivered (%)**	**Due services (%)**	**Brownout runs**	**All due success**
high leak / A	4125.50 [4125.46, 4125.54]	98.80 [98.23, 99.37]	100.00 [100.00, 100.00]	0/8	8/8
high leak / B	4125.50 [4125.46, 4125.54]	98.80 [98.23, 99.37]	100.00 [100.00, 100.00]	0/8	8/8
high leak / C	4857.79 [3997.15, 5718.42]	94.52 [85.05, 103.99]	99.87 [99.59, 100.15]	0/8	5/8
high leak / D	4559.65 [3890.21, 5229.10]	96.52 [94.16, 98.88]	99.97 [99.90, 100.04]	0/8	6/8
no harvest / A	4125.50 [4125.46, 4125.54]	98.80 [98.23, 99.37]	100.00 [100.00, 100.00]	0/8	8/8
no harvest / B	4125.50 [4125.46, 4125.54]	98.80 [98.23, 99.37]	100.00 [100.00, 100.00]	0/8	8/8
no harvest / C	4183.42 [4109.85, 4256.98]	97.29 [95.90, 98.68]	99.99 [99.97, 100.01]	0/8	6/8
no harvest / D	4918.90 [3767.77, 6070.03]	89.71 [77.15, 102.27]	93.89 [84.41, 103.37]	2/8	4/8

S5.4 Paired selector effects and interaction

Total-energy selection does not consistently reduce realized full-loop load across scenarios. Table S20 and Table S21 retain the within-seed contrasts at 1× and 8×. Positive A−B or C−D denotes lower load for the total-energy-selector arm; A−C and B−D describe gateway-policy changes. The interaction is the adaptive selector effect minus the fixed-period selector effect.

Figure S10 preserves the scenario-dependent signs and uncertainty. At 8×, C−D is negative under nominal, outage, and no-harvest conditions, but positive under mixed, hot, and high-leakage conditions. Every corresponding descriptive interval includes zero. The mixed-workload interaction intervals also include zero at both profiles. These findings restrict the main manuscript's selector interpretation to the declared finite catalogue and evaluation conditions. Because the baseline admits only EARLIEST candidates, the comparison includes scheduling-catalogue changes. Full-loop policies also complete different work; these joule contrasts do not establish equal-service savings. A gateway-policy effect on node load remains distinct from energy consumed by the gateway itself.

Table S20. Within-seed node-load contrasts in joules over 72 h, at 1×. Brackets show descriptive, unadjusted 95% t intervals across eight seeds.

Table S20					
**Scenario**	**A–B**	**C–D**	**A–C**	**B–D**	**Interaction**
nominal	0.000 [-0.000, 0.001]	0.182 [-0.229, 0.594]	-0.223 [-0.625, 0.178]	-0.041 [-0.074, -0.008]	0.182 [-0.229, 0.593]
mixed	0.001 [0.000, 0.002]	0.012 [-0.016, 0.039]	-0.090 [-0.125, -0.055]	-0.079 [-0.096, -0.063]	0.011 [-0.017, 0.039]
outage	0.000 [-0.000, 0.001]	0.024 [-0.017, 0.065]	-0.091 [-0.142, -0.041]	-0.067 [-0.100, -0.035]	0.024 [-0.018, 0.065]
hot	0.001 [-0.001, 0.003]	8.359 [-11.088, 27.806]	-0.477 [-1.069, 0.114]	7.881 [-11.688, 27.450]	8.358 [-11.089, 27.805]
high leak	0.004 [-0.002, 0.010]	0.685 [-2.016, 3.387]	-1.836 [-3.645, -0.026]	-1.155 [-2.777, 0.468]	0.681 [-2.021, 3.383]
no harvest	0.000 [0.000, 0.000]	0.004 [-0.060, 0.068]	-0.090 [-0.174, -0.007]	-0.087 [-0.162, -0.011]	0.004 [-0.060, 0.068]

Table S21. Corresponding 8× contrasts, preserving all six scenarios, five contrast definitions, and reported interval precision.

Table S21					
**Scenario**	**A–B**	**C–D**	**A–C**	**B–D**	**Interaction**
nominal	0.000 [0.000, 0.000]	-749.366 [-1912.385, 413.652]	-20.345 [-40.235, -0.454]	-769.711 [-1948.401, 408.980]	-749.366 [-1912.385, 413.652]
mixed	0.000 [0.000, 0.000]	218.830 [-479.585, 917.244]	-437.099 [-1083.755, 209.557]	-218.270 [-354.364, -82.175]	218.830 [-479.585, 917.244]
outage	0.000 [0.000, 0.000]	-442.805 [-1132.530, 246.921]	-103.571 [-140.077, -67.065]	-546.376 [-1270.613, 177.862]	-442.805 [-1132.530, 246.921]
hot	0.000 [0.000, 0.000]	414.051 [-657.966, 1486.069]	-619.261 [-1627.989, 389.468]	-205.209 [-574.220, 163.802]	414.051 [-657.966, 1486.069]

Table S21					
**Scenario**	**A–B**	**C–D**	**A–C**	**B–D**	**Interaction**
high leak	0.000 [0.000, 0.000]	298.132 [-415.527, 1011.791]	-732.288 [-1592.915, 128.340]	-434.156 [-1103.582, 235.271]	298.132 [-415.527, 1011.791]
no harvest	0.000 [0.000, 0.000]	-735.484 [-1910.447, 439.480]	-57.916 [-131.477, 15.644]	-793.400 [-1944.531, 357.731]	-735.484 [-1910.447, 439.480]

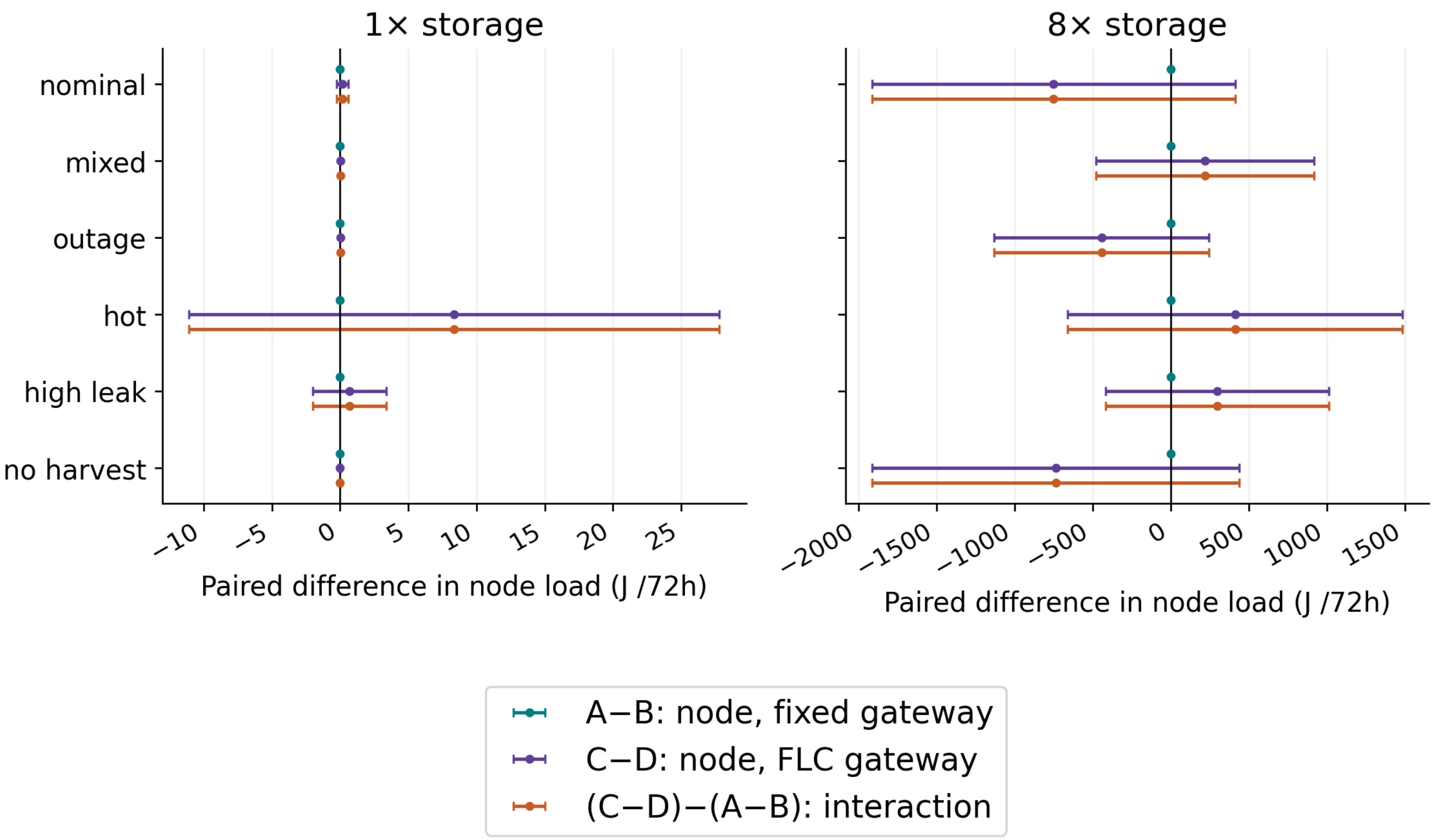

Figure S10. Selector effects and interaction at 1× (left) and 8× (right), using different horizontal scales. Points and intervals summarize eight paired seeds; the zero line is a reference.

S6 Leakage and Outage Behavior

S6.1 Leakage and first-brownout observations

Arm D's storage outcomes in Table S22 distinguish leakage expenditure from the occurrence and timing of a first brownout. At 1×, all eight runs cross the threshold in every scenario. Mean leakage rises from 12.50 J nominally to 49.46 J under heat and 123.64 J under increased leakage. The observed first-brownout means remain condition-specific, including 15.27 h without harvesting and 22.91 h nominally.

At 8×, the nominal, mixed, outage, hot, and high-leakage cells have no observed crossing during 72 h. The no-harvest D cell has two crossings, with a conditional mean of 55.02 h; no interval is supplied for that two-event subset. Leakage reaches 1322.98 J in the high-leakage cell despite its eight censored first-crossing times. These arm-D observations support a source-bounded interpretation of the main manuscript's storage stresses, not unconditional lifetime estimates. The separate full-cohort census contains 218 observed and 214 censored first-brownout times; it must not be mistaken for the twelve rows shown here. The adopted voltage–temperature law and jointly scaled resource profile describe model sensitivity rather than measured leakage calibration or a guarantee against depletion.

Table S22. Arm-D leakage and first-brownout outcomes over 72 h. Time means condition on observed crossings; n/8 identifies that subset. A dash denotes eight censored runs. Eight-observation intervals are descriptive 95% t intervals.

Table S22			
**Profile / scenario**	**Node leakage (J)**	**Brownout runs**	**First-brownout time**
1× / nominal	12.50 [12.48, 12.53]	8/8	22.91 [22.49, 23.34] h (8/8)
1× / mixed	12.47 [12.44, 12.49]	8/8	22.67 [22.21, 23.14] h (8/8)
1× / outage	12.47 [12.46, 12.49]	8/8	22.97 [22.82, 23.11] h (8/8)
1× / hot	49.46 [48.86, 50.06]	8/8	22.74 [22.36, 23.12] h (8/8)
1× / high leak	123.64 [123.11, 124.17]	8/8	22.11 [21.32, 22.90] h (8/8)
1× / no harvest	10.28 [10.26, 10.30]	8/8	15.27 [15.03, 15.51] h (8/8)
8× / nominal	138.89 [134.09, 143.70]	0/8	— (0/8)
8× / mixed	140.46 [139.79, 141.13]	0/8	— (0/8)
8× / outage	140.52 [139.04, 141.99]	0/8	— (0/8)
8× / hot	551.37 [539.47, 563.27]	0/8	— (0/8)
8× / high leak	1322.98 [1273.42, 1372.53]	0/8	— (0/8)
8× / no harvest	130.32 [125.52, 135.12]	2/8	55.02 h (2/8)

S6.2 Outage voltage, period and queue trajectories

The predesignated outage trajectory shows that gateway return, command changes, and queue evolution occur on different schedules. Figure S11 retains seed 1101 across all four arms and both resource profiles over 72 h. Gateway reception and transmission are unavailable from hour 12 until hour 48, while node states continue to evolve under their local service and energy rules. The plotted nominal period is a calendar quantity, not a direct measurement of sleep duration.

At 1×, the voltage traces reach the modeled brownout threshold and later exhibit recharge and interruption cycles. At 8×, the illustrated traces remain above that threshold, but full arm D develops substantial queue growth after gateway return. Thus, the absence of a threshold crossing does not imply satisfactory reporting or sustained service. A first restored communication event likewise does not establish that pending records have been cleared. This example expands the main manuscript's outage evaluation while preserving an adverse trajectory selected before outcome inspection. It is illustrative evidence from one seed, not a substitute for the eight-seed recovery summaries. Exact transition times and instantaneous states require the timestamped records; approximate values should not be inferred from raster coordinates.

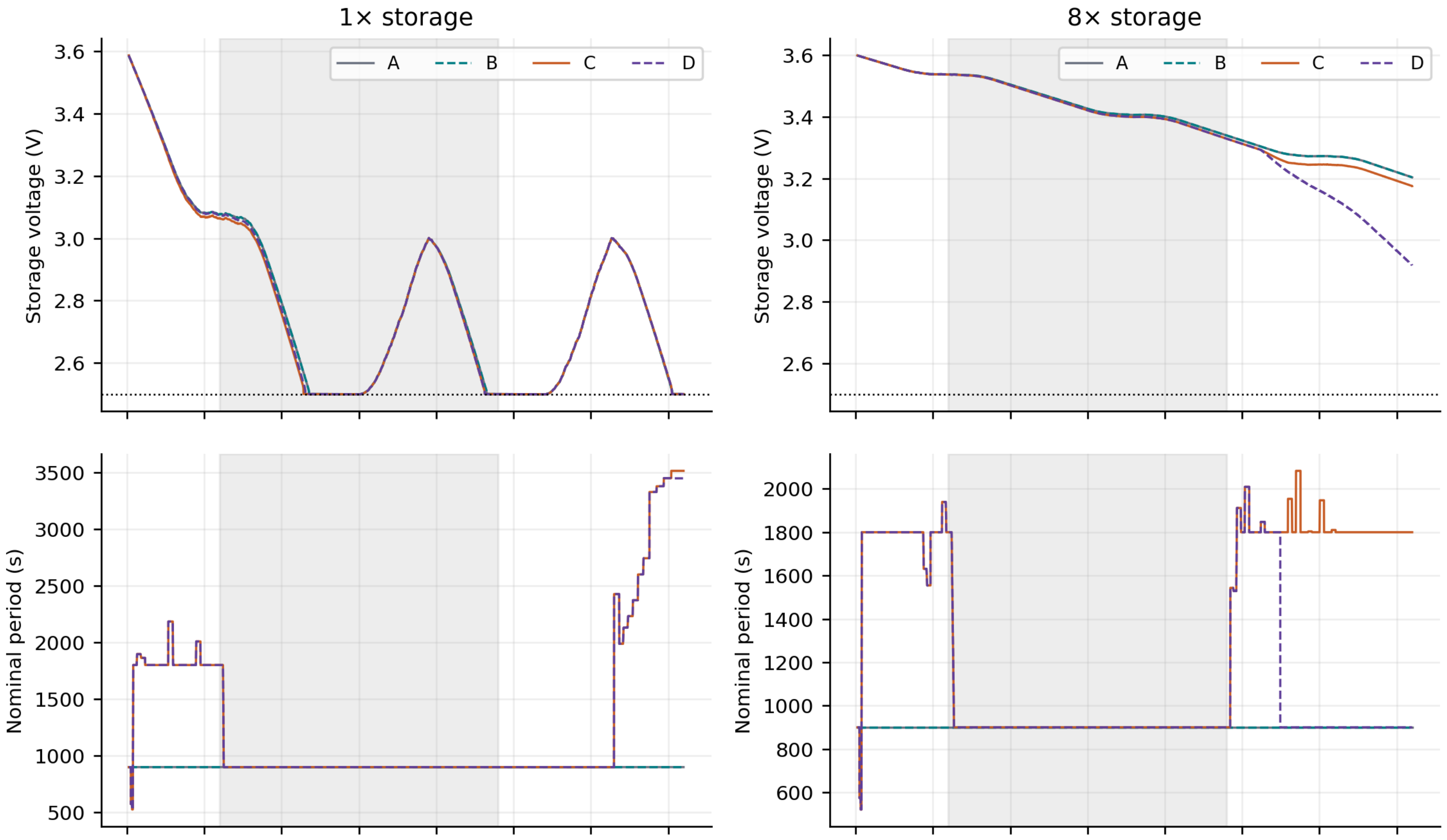
1× storage
8× storage
A
B
C
D
Storage voltage (V)
Nominal period (s)
3.6
3.4
3.2
3.0
2.8
2.6
3500
3000
2500
2000
1500
1000
500
1800
1600
1400
1200
800
600

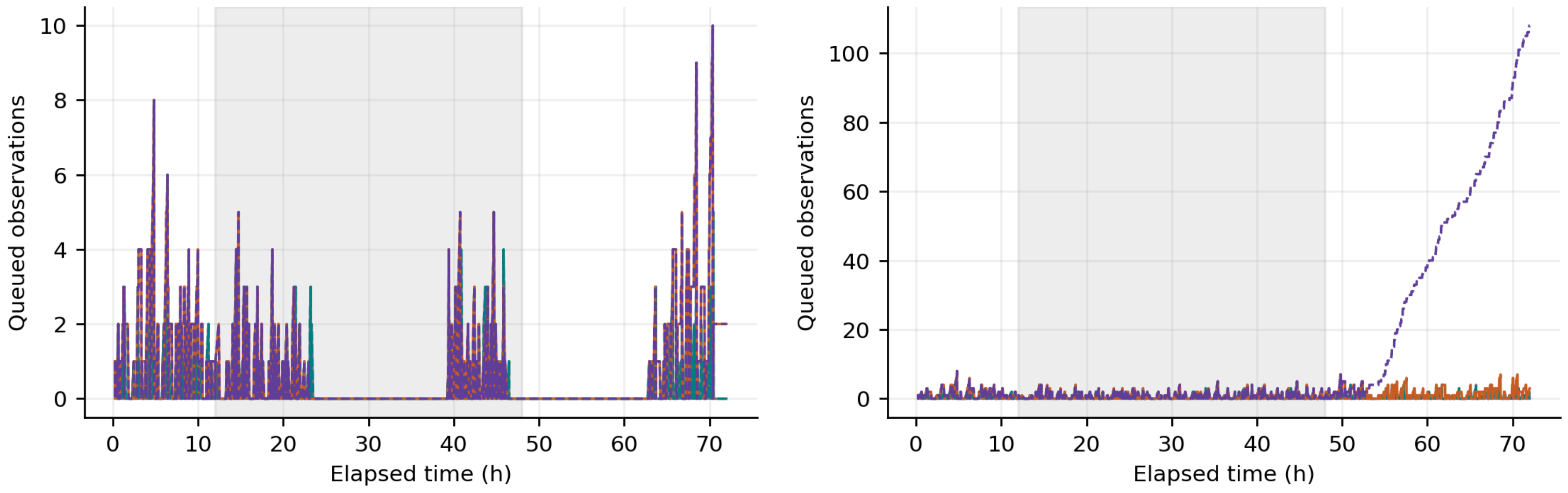

Figure S11. Predesignated outage seed 1101: voltage (top), nominal period (middle), and queue size (bottom); 1× left and 8× right. Shading marks gateway unavailability during 12–48 h; the dotted voltage line marks 2.5 V.

S6.3 Outage recovery endpoints

Recovery depends on the event being measured. Table S23 reports delays from gateway return at 48 h to first successful report delivery, accepted command reception, command application, and confirmation of the first complete predeclared service window. These times are elapsed seconds after return rather than absolute simulation timestamps. The service criterion uses a one-hour window on the 300 s grid and does not assert permanent recovery.

For arm D at 1×, the mean first-delivery delay is 53373.43 s, followed by accepted-command recovery at 53511.60 s and application at 54273.55 s. At 8×, the corresponding values are 395.50, 599.10, and 1295.62 s. All eligible printed rows have eight defined observations. Fixed arms A and B have no adaptive command or application endpoint, so their entries are structurally not applicable rather than zero or right-censored times. These distinctions clarify the main manuscript's dissemination interface and prevent gateway availability from being equated with operational recovery. Means condition on observed qualifying events; an eligible event absent by the horizon would remain censored. The separate gateway-processing diagnostics are unmodeled construction fields and cannot supply a measured zero latency.

Table S23. Outage recovery delays in seconds since gateway return at 48 h. Eligible cells have eight defined observations and descriptive 95% t intervals; fixed-policy command and application entries remain not applicable.

Table S23

Profile / arm	First delivery	Accepted command	Applied command	Service confirmed
1× / A	53212.40 [52466.73, 53958.06]	Not applicable	Not applicable	56587.50 [55750.96, 57424.04]
1× / B	53212.40 [52466.73, 53958.06]	Not applicable	Not applicable	56587.50 [55750.96, 57424.04]
1× / C	53386.91 [52517.29, 54256.52]	53511.60 [52675.06, 54348.14]	54287.01 [53417.39, 55156.63]	57037.50 [55307.96, 58767.04]
1× / D	53373.43 [52504.99, 54241.88]	53511.60 [52675.06, 54348.14]	54273.55 [53405.11, 55142.00]	56587.50 [55750.96, 57424.04]
8× / A	899.89 [899.86, 899.93]	Not applicable	Not applicable	3600.00 [3600.00, 3600.00]
8× / B	899.89 [899.86, 899.93]	Not applicable	Not applicable	3600.00 [3600.00, 3600.00]
8× / C	398.37 [124.29, 672.45]	599.10 [366.90, 831.30]	1298.49 [1024.41, 1572.57]	3600.00 [3600.00, 3600.00]
8× / D	395.50 [121.28, 669.72]	599.10 [366.90, 831.30]	1295.62 [1021.40, 1569.84]	3600.00 [3600.00, 3600.00]

S6.4 Node, loss and gateway energy boundaries

Energy conclusions depend on the accounting boundary. Table S24 and Table S25 report arm D separately at 1× and 8×. Node load integrates service, mode residence, transitions, and controller execution. Node draw additionally includes storage leakage and other configured physical losses; the latter are zero in the declared profile. Stored energy and the reserve constraint are not additional expenditure terms.

At 1×, mixed-workload node load is 2249.86 J and node draw is 2262.33 J. At 8× under high leakage, the respective means are 4559.65 and 5882.63 J, making the loss boundary consequential. Gateway incremental energy separately combines available-gateway computation attempts with modeled transmission and reception. Its declared 1 W baseline contributes 259200 J over the complete 72 h horizon, including the communication outage, and is excluded from the incremental columns. The studied node-plus-gateway total therefore includes node draw, baseline, and increment; it excludes background-device consumption. These definitions elaborate the main manuscript's energy metrics while preventing a node-load reduction from being presented automatically as a system-energy reduction. Energy associated with already-transmitted background packets that subsequently fail is attributed work, not a second energy charge.

Table S24. Arm-D energy boundaries at 1×, in joules over 72 h. Each row gives eight-seed means and descriptive 95% t intervals.

Table S24				
**Scenario**	**Node load**	**Leakage**	**Node draw**	**Gateway increment**
nominal	2249.82 [2236.79, 2262.85]	12.50 [12.48, 12.53]	2262.32 [2249.28, 2275.37]	21.51 [21.12, 21.90]
mixed	2249.86 [2236.83, 2262.89]	12.47 [12.44, 12.49]	2262.33 [2249.28, 2275.37]	21.70 [21.32, 22.09]
outage	2249.85 [2236.81, 2262.89]	12.47 [12.46, 12.49]	2262.32 [2249.28, 2275.37]	10.90 [10.78, 11.02]
hot	2204.90 [2185.03, 2224.76]	49.46 [48.86, 50.06]	2254.36 [2234.00, 2274.72]	19.92 [17.65, 22.19]
high leak	2140.72 [2127.17, 2154.28]	123.64 [123.11, 124.17]	2264.36 [2250.92, 2277.80]	20.38 [19.48, 21.29]
no harvest	903.27 [903.19, 903.35]	10.28 [10.26, 10.30]	913.55 [913.49, 913.61]	9.28 [9.15, 9.41]

Table S25. Corresponding arm-D boundaries at 8×. Gateway increments exclude the separately declared baseline; node draw includes physical storage losses.

Table S25				
**Scenario**	**Node load**	**Leakage**	**Node draw**	**Gateway increment**
nominal	4895.21 [3716.54, 6073.88]	138.89 [134.09, 143.70]	5034.10 [3860.19, 6208.02]	33.93 [26.65, 41.21]
mixed	4346.44 [4210.34, 4482.54]	140.46 [139.79, 141.13]	4486.90 [4351.32, 4622.48]	37.59 [37.55, 37.63]
outage	4671.87 [3947.65, 5396.10]	140.52 [139.04, 141.99]	4812.39 [4089.64, 5535.15]	16.14 [12.54, 19.73]
hot	4330.71 [3961.71, 4699.71]	551.37 [539.47, 563.27]	4882.08 [4524.97, 5239.19]	37.03 [37.02, 37.04]
high leak	4559.65 [3890.21, 5229.10]	1322.98 [1273.42, 1372.53]	5882.63 [5261.11, 6504.15]	36.66 [35.90, 37.42]
no harvest	4918.90 [3767.77, 6070.03]	130.32 [125.52, 135.12]	5049.22 [3902.88, 6195.55]	34.00 [29.32, 38.68]

S7 Radio and Communication Costs

S7.1 Downlink and receiver costs

Adaptive communication entails distinct downlink, node-listening, and gateway costs. Table S26 reports arm D across all six scenarios at each profile, including the node RX increment that is absent from some broader energy summaries. At 1×, nominal downlink airtime is 39.75 s with a 1.492 J RX increment and 21.51 J gateway increment. Under outage, airtime is 20.05 s, whereas the RX increment remains 1.493 J. Gateway unavailability therefore does not remove the separately scheduled listening obligation.

The 8× mixed cell has 68.88 s downlink airtime, 2.850 J RX increment, and 37.59 J gateway increment. These quantities describe different energy boundaries and cannot be added indiscriminately to node load. The RX increment is the configured above-M0 contribution allocated to actual funded execution, including partial phases; the full phase load already enters node-load accounting. Periodic listening is shared by all arms and is not itself an adaptive-minus-fixed expense. The prescribed copies also occur without success-dependent ACK retries. These results extend the main manuscript's communication-overhead evaluation while retaining direct background losses as a separate outcome and avoiding claims about the entire network's energy response.

Table S26. Arm-D communication costs over 72 h at both profiles. Downlink airtime, above-M0 RX energy, gateway increment, and new background-copy losses are separate eight-seed means with descriptive 95% t intervals.

Table S26				
**Profile / scenario**	**Downlink airtime (s)**	**Node RX increment (J)**	**Gateway increment (J)**	**Newly lost background copies**
1× / nominal	39.75 [39.00, 40.50]	1.492 [1.465, 1.518]	21.51 [21.12, 21.90]	2.12 [0.55, 3.70]
1× / mixed	39.81 [39.03, 40.59]	1.478 [1.457, 1.498]	21.70 [21.32, 22.09]	7.12 [3.98, 10.27]
1× / outage	20.05 [19.82, 20.28]	1.493 [1.480, 1.506]	10.90 [10.78, 11.02]	0.88 [-0.34, 2.09]
1× / hot	36.67 [32.26, 41.08]	1.377 [1.237, 1.516]	19.92 [17.65, 22.19]	1.88 [0.74, 3.01]
1× / high leak	37.59 [35.83, 39.35]	1.390 [1.314, 1.467]	20.38 [19.48, 21.29]	2.00 [0.66, 3.34]
1× / no harvest	16.16 [15.91, 16.41]	0.602 [0.592, 0.612]	9.28 [9.15, 9.41]	1.00 [-0.18, 2.18]
8× / nominal	63.02 [49.16, 76.88]	2.768 [2.574, 2.962]	33.93 [26.65, 41.21]	2.88 [1.12, 4.63]
8× / mixed	68.88 [68.88, 68.88]	2.850 [2.849, 2.852]	37.59 [37.55, 37.63]	9.12 [5.51, 12.74]
8× / outage	29.86 [23.05, 36.67]	2.851 [2.850, 2.852]	16.14 [12.54, 19.73]	1.25 [0.09, 2.41]
8× / hot	68.88 [68.88, 68.88]	2.851 [2.850, 2.852]	37.03 [37.02, 37.04]	3.00 [1.33, 4.67]
8× / high leak	68.27 [66.83, 69.71]	2.851 [2.850, 2.852]	36.66 [35.90, 37.42]	3.00 [1.33, 4.67]
8× / no harvest	63.14 [54.25, 72.03]	2.670 [2.388, 2.952]	34.00 [29.32, 38.68]	2.75 [1.15, 4.35]

S7.2 Direct radio effects within the current campaign

The current radio counterfactual isolates direct overlap effects within arm D's realized activity. Table S27 and Table S28 retain attempted downlinks, airtime, newly lost background copies, and energy already spent transmitting those copies at 1× and 8×. Removing downlinks holds offered background traffic and actual target occupation fixed; it does not rerun the adaptive policy or allow peers to change their behavior.

In mixed traffic, mean new background losses are 7.12 copies at 1× and 9.12 at 8×, associated with already-spent peer transmission energy of 0.0427 and 0.0547 J. These are attributed failed transmissions, not additional consumption to add to the node-plus-gateway ledger. No extra offered peer transmissions or reactive retries are generated by this counterfactual. Other peer reception and processing remain outside its boundary. Loss causes can overlap, so cause-specific counts are not disjoint totals. The retained untruncated intervals may have negative lower endpoints for nonnegative

counts; those endpoints do not represent observed negative losses. This controlled comparison clarifies the direct communication effect relevant to the main manuscript while leaving the full network's endogenous energy response unestimated.

Table S27. Current arm-D direct radio outcomes at 1× over 72 h: eight-seed means and descriptive 95% t intervals, including already-spent peer transmission energy.

Table S27				
**Scenario**	**Downlink attempts**	**Downlink airtime (s)**	**New background losses**	**Already-spent peer TX (J)**
nominal	993.75 [975.08, 1012.42]	39.75 [39.00, 40.50]	2.12 [0.55, 3.70]	0.0128 [0.0033, 0.0222]
mixed	995.25 [975.79, 1014.71]	39.81 [39.03, 40.59]	7.12 [3.98, 10.27]	0.0427 [0.0239, 0.0616]
outage	501.25 [495.46, 507.04]	20.05 [19.82, 20.28]	0.88 [-0.34, 2.09]	0.0052 [-0.0021, 0.0126]
hot	916.75 [806.47, 1027.03]	36.67 [32.26, 41.08]	1.88 [0.74, 3.01]	0.0112 [0.0044, 0.0181]
high leak	939.75 [895.75, 983.75]	37.59 [35.83, 39.35]	2.00 [0.66, 3.34]	0.0120 [0.0040, 0.0200]
no harvest	404.00 [397.68, 410.32]	16.16 [15.91, 16.41]	1.00 [-0.18, 2.18]	0.0060 [-0.0011, 0.0131]

Table S28. Corresponding 8× outcomes under the same fixed-occupation counterfactual. Attributed peer transmission energy is not an additional energy debit.

Table S28				
**Scenario**	**Downlink attempts**	**Downlink airtime (s)**	**New background losses**	**Already-spent peer TX (J)**
nominal	1575.50 [1229.08, 1921.92]	63.02 [49.16, 76.88]	2.88 [1.12, 4.63]	0.0173 [0.0067, 0.0278]
mixed	1722.00 [1722.00, 1722.00]	68.88 [68.88, 68.88]	9.12 [5.51, 12.74]	0.0547 [0.0331, 0.0764]
outage	746.50 [576.34, 916.66]	29.86 [23.05, 36.67]	1.25 [0.09, 2.41]	0.0075 [0.0005, 0.0145]
hot	1722.00 [1722.00, 1722.00]	68.88 [68.88, 68.88]	3.00 [1.33, 4.67]	0.0180 [0.0080, 0.0280]

Table S28				
**Scenario**	**Downlink attempts**	**Downlink airtime (s)**	**New background losses**	**Already-spent peer TX (J)**
high leak	1706.75 [1670.69, 1742.81]	68.27 [66.83, 69.71]	3.00 [1.33, 4.67]	0.0180 [0.0080, 0.0280]
no harvest	1578.50 [1356.14, 1800.86]	63.14 [54.25, 72.03]	2.75 [1.15, 4.35]	0.0165 [0.0069, 0.0261]

S7.3 Fixed-trace downlink collision replay

A separate fixed-trace replay asks how predetermined downlink copies change geometric collisions in one historical mixed-workload realization. Table S29 preserves start-to-start copy spacings of 0.04, 0.06, and 0.10 s. From the same baseline of 244 other-device losses, inserting downlinks produces 13, 14, and 16 additional losses, respectively. The target experiences no newly lost attempt in this trace. Endpoint contact alone is excluded from collision.

Each copy lasts 0.04 s, making the shortest spacing adjacent transmission and the longer settings include silence. The 1002 downlink attempts occupy 40.08 s and consume 20.040 J at the declared gateway transmission power of 0.5 W. Peer transmission energies associated with newly lost packets were already expended and do not constitute added consumption. The three settings and 39654 recorded packet rows are not independent seeds or additional members of the current 432-run campaign. This replay supports the direct-collision question raised by the main manuscript's communication interface, while retaining its narrower historical scope. It excludes capture, distinct-channel or spreading-factor decoding, success-dependent retransmission, receiver-listening energy, and network-wide adaptation; consequently, it cannot establish an aggregate adaptive-network energy benefit.

Table S29. Historical seed-1101 packet replay for three start-to-start downlink-copy spacings. Counts distinguish baseline other-device losses, losses after insertion, newly lost attempts, and downlink failures; no sampling intervals are inferred.

Table S29				
**Copy spacing s**	**Other losses before**	**Other losses with DL**	**New other losses**	**DL losses**
0.04	244	257	13	21
0.06	244	258	14	22
0.10	244	260	16	21

S8 Implementation and Verification

S8.1 Common-service selector replay

The common-service replay evaluates selector energy under obligations that remain genuinely comparable. It comprises 63 deterministic cases crossing three workloads, seven horizons, and three available-energy levels. Required service, comparison horizon, and terminal conditions are held fixed, with transition and controller costs included. Both selectors have a feasible plan in 54 cases. Within those pairs, the total-energy selector has lower estimated cost in 46 and equal cost in eight; no higher-cost selection is reported.

The remaining nine cases lack a feasible pair and are not assigned zero saving. Their absence from the paired comparison is a feasibility outcome, not evidence of equality or an omitted unfavorable energy measurement. The recorded verification checks 2520 catalogue entries and the selected minima. Those entry checks are arithmetic assessments of the supplied cases, not additional experiments or independent statistical replications. The comparison therefore supports minimum estimated cost within the declared feasible catalogue under common obligations.

This result clarifies the node-selection mechanism in the main manuscript and complements the full-loop contrasts in Section **Hata! Başvuru kaynağı bulunamadı.**, where realized service can differ between policies. A finite-catalogue replay does not establish a globally optimal physical schedule, measured hardware savings, or improved delivery in an evolving network. The distinction permits a favorable selector result to be retained without extending it beyond the conditions that make its energy comparison interpretable.

S8.2 Bounded implementation resource requirements

The implementation evidence concerns a bounded C subsystem that assesses caller-supplied phases, selects plans, and implements command validation and the nominal calendar. Table S30 compares 20-, 5-, and 3-mode builds while holding capacities at 64 jobs, 320 phases, and 40 plans. Flash-section sums are 3626, 3262, and 3214 bytes; the corresponding padding-inclusive spans are 3632, 3264, and 3216 bytes. Static RAM remains 24576 bytes in every build.

The sum of seven named compiler frames is 484 bytes, which is neither a whole-program stack bound nor a measured high-water mark. The declared ARM Cortex-M4 Thumb EABI target also differs from the FiPy or ESP32 reference hardware. Candidate construction, the persistent service book, carryover, recovery execution, event handling, physical accounting, the FLC, drivers, and RTOS lie outside this port. Its adapter rounds supplied load and loss fields upward and energy budgets downward within a bounded interface; this does not extend exact rational scheduling to arbitrary C inputs. Native fixtures compare subsystem behavior with Python without executing the target ELF on an MCU. The result supplies partial implementation context for the main manuscript's local planner, not full-controller resource, timing, or energy validation.

Table S30. Supplied-phase C subsystem resources in bytes for three mode-catalogue sizes. Section sums, padding-inclusive flash spans, and static BSS allocation retain their separate meanings and source precision.

Table S30							
**Modes**	**.text**	**.rodata**	**exidx**	**.data**	**.bss**	**Flash sum**	**Flash span**

Table S30							
**Modes**	**.text**	**.rodata**	**exidx**	**.data**	**.bss**	**Flash sum**	**Flash span**
20	3082	480	64	0	24576	3626	3632
5	3078	120	64	0	24576	3262	3264
3	3078	72	64	0	24576	3214	3216

S8.3 Exact scheduling representation and recovery additions

Exact scheduling representation preserves decimal times without introducing an admission tolerance. Table S31 separates this representation from the declared recovery additions and their costs. Decimal inputs are represented as rational seconds; adapting an already rounded binary float cannot recover precision lost before conversion. A 2.5 ms transition therefore remains distinguishable from a blanket 1 ms timing grid.

Boundary checks cover equality and offsets of ±1 ns around the five mode thresholds at six translated origins: 0, 1, 60, 300, 1000, and 259199 s. These checks address comparison semantics rather than processor timing accuracy. The FLC's binary-floating-point flooring operation is separate and retains its reported small command-lattice residuals. This distinction supports the manuscript's executable method description: exact calendar admission, numerical controller output, and physical integration have different arithmetic contracts. The recovery constants remain declared assumptions, and the repeated search expenditure is modeled control cost rather than measured MCU execution energy.

Table S31. Exact-time representation and declared recovery additions, with their cost and terminal-equivalence scope.

Table S31		
**Field**	**Value and unit**	**Basis and scope**
Scheduling time	Exact rational seconds; zero admission tolerance	Derived representation; source decimal precision is preserved
recovery_max_horizon	60 s	Declared recovery assumption based on sensing cadence and distant-conflict diagnosis
recovery_tail	1 s	Declared deadline tail for the common near horizon
Attempted full plus near control	0.002 s; two 10 μJ incremental fees plus M0 power	Two copies of the retained per-search cost; no MCU runtime claim
Equivalent terminal	M0 plus complete service-workload fingerprint	Pending definitions and actual outcomes preserved; energy excluded

S8.4 Recovery sensitivity fixtures

The bounded recovery sensitivity check varies a declared horizon and deadline tail, rather than estimating a population response. Near-horizon limits of 30, 60, and 120 s are crossed with tails of 0.5, 1, and 2 s for each of three fixtures. This yields 27 diagnostic cases. Table S32 contains three summary rows, each covering nine settings; it is not a table of 27 individual observations.

For the motivating fixture, every setting completes the near sensing/processing pair while retaining the original target. Actual node load spans 1.035788–1.056788 J. The two application fixtures retain no sensing miss and one true transmission miss per setting, with load ranges of 16.168966–17.541403 J and 72.442696–116.357607 J. These descriptive ranges compare the tested settings within each fixture; they are not independently estimated confidence intervals.

These diagnostics support a limited interpretation of the manuscript's recovery mechanism: the tested parameter changes preserve the listed service outcomes while changing cost. They do not erase the transmission failures or identify a universal recovery optimum. The retained 60 s and 1 s defaults are declared choices, not values selected retrospectively from the most favorable case. Fixture counts are not campaign replicates or lifetime observations.

Table S32. Three fixture summaries, each covering nine horizon/tail combinations; ranges describe actual node load across settings.

Table S32			
**Case**	**Rows**	**Actual node load range J**	**Service and scope**
M1	9	1.035788 to 1.056788	Near pair complete; target retained
A1_60.05	9	16.168966 to 17.541403	No sensing miss; one true TX miss per row
A1_2520.155	9	72.442696 to 116.357607	No sensing miss; one true TX miss per row

S8.5 Controller fixtures and independent energy oracle

The current controller was checked against bounded fixtures using an independent configuration-based energy oracle. The source reports 72 core checks, 35 runtime checks, four principal fixtures spanning 160 full candidates, and an additional forty-candidate near search. Expected costs are not obtained by asking the production selector to certify itself.

The application fixtures use commands of 60.05 and 2520.155 s, retain true misses, and distinguish reception from application. The interruption fixture charges the full exit and 0.2595 J. Equality tests cover all five thresholds at six translated origins, while malformed cases exercise controlled failure. A separate brownout fixture crosses at 0.07713693845109414 s within an RX phase ending at 0.101 s; reception does not complete during OFF operation.

In the motivating recovery case, the full forty-candidate catalogue is rejected. A near prefix ending at 73921.1 s selects EARLIEST:M18, with estimated cost 1.056832 J and actual prefix load 1.042788 J. Sensing and processing complete at 73920.02 and 73920.06 s, respectively. Two actual searches cost 0.00348 J over 0.002 s. The scoped predecessor completed no service and consumed 24.29614 J; the successor inventory contains 80 rather than 79 jobs by retaining a processing obligation. These fixtures

explain the manuscript's correction of a stalled service path. They establish the listed bounded behaviors, not equivalence of complete historical trajectories or a guarantee that arbitrary workloads avoid rejection, missed service, or brownout.

S8.6 Command lattice residuals and exact recurrences

Exact calendar recurrence and nominal command quantization answer different numerical questions. The source reports small deviations from the intended millisecond lattice in 10533 of 143080 generated commands and 1630 of 23446 application records, with maximum absolute residual 3×10^{-13} s in each set. These residuals arise from the retained binary-floating-point flooring path. Cached reapplications are separate events rather than fresh gateway computations.

All 89676 checked nominal recurrences satisfy their exact rational relation. Preserving the decimal value produced by the controller therefore makes subsequent scheduling exact without retroactively moving that value onto an ideal lattice. Replacing the quantizer with a different exact construction might change boundary decisions; equivalence of that alternative is not established by the small observed residual alone.

The source also records 400, 400, and 368 normalizations of optional exact companion fields in execution wrappers. These apply where the underlying value is already undefined. They do not impute physical energy, successful recovery, or a missing command outcome. Together, the checks refine the manuscript's numerical reproducibility claim: recurrence identities can hold while controller outputs retain floating-point residue. Reported source identities and bounded execution checks remain distinct from a proof about every runtime callable or every possible input. None of these counts supplies a statistical confidence interval for controller performance.

S8.7 Retained centroid-reference checks and unresolved integration step

The retained controller verification separates agreement of saved values from reproducibility of the numerical reference. Forty-eight raw-centroid/reference pairs have a reported maximum absolute difference below 2.70×10^{-7} s. Their saved differences were recalculated in the source work. A separate set of 48 boundary records contains test names and PASS flags, without detailed actual and expected values or exception traces.

The trapezoidal reference verifier specifies a 0.025 s output-integration step and calls it without an override, whereas the preregistration specifies 0.01 s. The saved result does not record the step or a corresponding invocation, so execution with the registered resolution is not established. This discrepancy concerns integration over the output period. It is distinct from ±0.01 increments in normalized inputs used for finite-difference sensitivity and from the controller's analytical deployed centroid. Treating these three quantities as one numerical setting would conceal the unresolved reference configuration.

Two source checkers independently reduced 37,044 aligned full and reduced-controller grid rows. That component evidence complements the manuscript's static numerical characterization, while the boundary flags provide only their retained level of detail. Neither the reference integral nor the boundary tests was newly executed for this supplement. Small saved pair differences consequently support scoped numerical agreement, without demonstrating execution of the preregistered reference procedure, completeness of missing boundary diagnostics, or global closed-loop behavior.

S8.8 Historical eight-job compiled subsystem

An earlier ARM Cortex-M4 Thumb reference target implements a bounded C mode and job selector with a command/calendar subsystem. Its three builds use 20, 5, and 3 modes. Reported flash section sums are respectively 1910, 1670, and 1638 bytes, while padding-inclusive spans are 1916, 1676, and 1644 bytes. All three report 68 bytes of static RAM section payload and 72 bytes after alignment. Distinguishing section sums from address spans prevents counting padding as additional implemented functionality.

The subsystem accepts at most eight caller-supplied jobs. It omits controller computation, leakage estimation, several service constraints present in the Python implementation, the FLC, device drivers, and complete physical execution. The mode-count comparison therefore measures the static footprint of this bounded target, not the complete HA-PM controller. The supplied build context and arithmetic fixtures do not establish measured processor latency or operating energy, and no fresh compilation is reported here.

These results remain separate from the later reference port with capacities of 64 jobs, 320 phases, and 40 plans, which reports 24576 bytes of static RAM and flash sums of 3626, 3262, and 3214 bytes. The different implementation boundaries explain why equal mode counts do not identify equivalent targets. Together, the scoped records qualify the manuscript's implementation discussion without supporting whole-firmware equivalence, prototype performance, or a measured energy advantage from reducing the catalogue.

S8.9 Reported numerical consistency and execution provenance

Numerical consistency and execution provenance address different layers of the reported simulation evidence. Across the current 432 runs, the source gives a maximum absolute cumulative ledger residual at phase checkpoints of $1.547050487715751 \times 10^{-9}$ J and an end-of-run balance residual of $1.546140993013978 \times 10^{-9}$ J. These distinct metrics accompany independent readdition of exported energy terms. The historical 216-run campaign instead reports 4.14×10^{-10} J; its preserved failed first-main attempt contributes no completed-campaign result.

The retained integrated-core notebook reports eight code cells, eight saved output bundles, and 40 captured kernel messages from in-process execution. Its earlier refusal of a stale C binding occurred before execution and remains preserved. These deterministic examples do not execute the complete campaign. The source also declares 5724 outputs, eight outage-state traces, and detailed phase/service pairs for 54 seed-1101 runs, covering 27 cells at two resource scales; the other 378 runs lack those detailed exports.

The present identity review establishes the 432-entry roster and 41 loaded configurations, without claiming to have reread all declared streams or state traces. Conservation closure supports internal accounting but cannot validate a shared incorrect physical input model. These separate evidence layers delimit the manuscript's computational claims: provenance binds reported artifacts, numerical checks test defined calculations, and neither substitutes for component calibration, physical validation, or complete independent campaign reproduction.

S9 Parameter and Evidence Registers

S9.1 Mode powers and their evidence boundary

The mode-power register distinguishes document-supported endpoints from constructed intermediate values. Table S33 preserves all twenty full-precision powers, including the M1–M14 interpolation across the retained range. Those individual intermediate powers are declared reference values rather than recovered device measurements.

Hata! Başvuru kaynağı bulunamadı. records the associated evidence and interpretation, while **Hata! Başvuru kaynağı bulunamadı.** retains the exact shared provenance fields and their applicable identifiers. Sharing an identical note does not extend it to unrelated parameter domains. These distinctions qualify the manuscript's mode-energy model: agreement with a source number establishes traceability, whereas physical calibration would require independent measurement evidence. The retained register also preserves its version history; later exact-clock and recovery rules are stated separately rather than silently replacing historical entries.

Table S33. Full-precision powers for all twenty declared modes.

Table S33		
**Parameter**	**Value**	**Unit**
M0.power_w	1.73	W
M1.power_w	1.64	W
M2.power_w	1.543076923076923	W
M3.power_w	1.4461538461538461	W
M4.power_w	1.3492307692307692	W
M5.power_w	1.2523076923076923	W
M6.power_w	1.1553846153846155	W
M7.power_w	1.0584615384615383	W
M8.power_w	0.9615384615384616	W
M9.power_w	0.8646153846153847	W
M10.power_w	0.7676923076923078	W
M11.power_w	0.6707692307692308	W
M12.power_w	0.5738461538461539	W
M13.power_w	0.476923076923077	W
M14.power_w	0.38000000000000034	W
M15.power_w	0.3	W

Table S33		
**Parameter**	**Value**	**Unit**
M16.power_w	0.26	W
M17.power_w	0.11	W
M18.power_w	0.014	W
M19.power_w	0.0007	W

S9.2 Inherited transition conventions and admission units

Table S34 preserves the entry/exit quantities associated with the declared mode catalogue. The retained total latency is split equally between entry and exit, and each transition receives full M0 power multiplied by its duration. Adding another baseline charge would double count that expenditure.

The admission values expressed in seconds correspond to 7–300 ms; they are distinct from the historical seconds-threshold auxiliary model. These records support the manuscript's implementation boundary without turning an equal transition split into measured component timing. The author-fixed admission rule remains separate from an experimentally established break-even calculation.

Table S34. Retained entry/exit durations and energy debits for every mode.

Table S34		
**Parameter**	**Value**	**Unit**
M0.enter_s	0.0	s
M0.exit_s	0.0	s
M0.enter_j	0.0	J
M0.exit_j	0.0	J
M1.enter_s	0.0	s
M1.exit_s	0.0	s
M1.enter_j	0.0	J
M1.exit_j	0.0	J
M2.enter_s	0.0	s
M2.exit_s	0.0	s
M2.enter_j	0.0	J
M2.exit_j	0.0	J

Table S34		
**Parameter**	**Value**	**Unit**
M3.enter_s	0.0	s
M3.exit_s	0.0	s
M3.enter_j	0.0	J
M3.exit_j	0.0	J
M4.enter_s	0.0	s
M4.exit_s	0.0	s
M4.enter_j	0.0	J
M4.exit_j	0.0	J
M5.enter_s	0.0	s
M5.exit_s	0.0	s
M5.enter_j	0.0	J
M5.exit_j	0.0	J
M6.enter_s	0.0	s
M6.exit_s	0.0	s
M6.enter_j	0.0	J
M6.exit_j	0.0	J
M7.enter_s	0.0	s
M7.exit_s	0.0	s
M7.enter_j	0.0	J
M7.exit_j	0.0	J
M8.enter_s	0.0	s
M8.exit_s	0.0	s
M8.enter_j	0.0	J
M8.exit_j	0.0	J
M9.enter_s	0.0	s
M9.exit_s	0.0	s

Table S34		
**Parameter**	**Value**	**Unit**
M9.enter_j	0.0	J
M9.exit_j	0.0	J
M10.enter_s	0.0	s
M10.exit_s	0.0	s
M10.enter_j	0.0	J
M10.exit_j	0.0	J
M11.enter_s	0.0	s
M11.exit_s	0.0	s
M11.enter_j	0.0	J
M11.exit_j	0.0	J
M12.enter_s	0.0	s
M12.exit_s	0.0	s
M12.enter_j	0.0	J
M12.exit_j	0.0	J
M13.enter_s	0.0	s
M13.exit_s	0.0	s
M13.enter_j	0.0	J
M13.exit_j	0.0	J
M14.enter_s	0.0	s
M14.exit_s	0.0	s
M14.enter_j	0.0	J
M14.exit_j	0.0	J
M15.enter_s	0.0025	s
M15.exit_s	0.0025	s
M15.enter_j	0.004325	J
M15.exit_j	0.004325	J

Table S34		
**Parameter**	**Value**	**Unit**
M16.enter_s	0.005	s
M16.exit_s	0.005	s
M16.enter_j	0.00865	J
M16.exit_j	0.00865	J
M17.enter_s	0.01	s
M17.exit_s	0.01	s
M17.enter_j	0.0173	J
M17.exit_j	0.0173	J
M18.enter_s	0.09	s
M18.exit_s	0.09	s
M18.enter_j	0.1557	J
M18.exit_j	0.1557	J
M19.enter_s	0.15	s
M19.exit_s	0.15	s
M19.enter_j	0.2595	J
M19.exit_j	0.2595	J

S9.3 Sensing, processing and radio service settings

The service settings specify the workload against which the current node makes local energy decisions. Table S35 retains the sensing cadence, phase durations, deadlines, reception schedule, report duration, queue capacity, and record/packet sizes. These values define timed obligations and payload capacity rather than a measured workload distribution on a deployed device.

Sensing and processing costs are added above the common M0 baseline; interpreting them as complete phase energies and charging M0 again would change the accounting. Radio increments likewise depend on the adopted voltage and duration. Their full decimal representations remain unchanged so that displayed rounding does not become a new executable configuration.

The manuscript's service and delivery interpretation depends on these distinctions. Requested sensing is independent of the adaptive report period, processing retains its sensing predecessor, and available payload depends on completed work. A longer reporting interval therefore changes neither the requested sensing cadence nor the physical meaning of an observation. The registered sensitivity

ranges describe declared parameter uncertainty choices; they are not confidence intervals, device tolerances established by measurement, or evidence that every listed perturbation was executed.

Table S35. Declared sensing, processing, reception, transmission, and payload settings.

Table S35		
**Parameter**	**Value**	**Unit**
service.sensing_period_s	60.0	s
service.sensing_duration_s	0.02	s
service.sensing_incremental_j	0.012	J
service.sensing_deadline_s	0.1	s
service.sensing_start_slack_s	0.01	s
service.processing_duration_s	0.04	s
service.processing_incremental_j	0.11	J
service.processing_deadline_s	0.2	s
service.rx_period_s	300.0	s
service.rx_duration_s	0.1	s
service.rx_incremental_j	0.0033000000000000004	J
service.rx_completion_offset_s	-0.9	s
service.tx_duration_s	0.24	s
service.tx_incremental_j	0.022175999999999998	J
service.event_window_s	3600.0	s
service.queue_capacity_items	1000	items
service.packet_capacity_bytes	250	bytes
service.item_bytes	10	bytes

S9.4 Storage and reserve assumptions

Storage capacity, operational thresholds, and planning reserves play different roles. Table S36 retains capacitance, energy limits, initial energy, leakage settings, observation uncertainty, and estimated loss. For the supercapacitor, the initial 3.6 V and restart 3.0 V values are model choices, distinct from the 2.5 V brownout boundary.

The 5 J reserve constrains admission; it is not an extra energy debit. Similarly, the 1 mW estimated loss is a planning assumption rather than a guaranteed upper bound on the physical temperature-

dependent leakage law. Zero configured observation uncertainty or other loss does not establish that a physical device has no uncertainty or additional consumption.

These distinctions qualify the manuscript's energy-reserve interpretation. Numerical traceability of 1949.4 J capacity and 843.75 J brownout energy follows from declared capacitor values, without validating component behavior under load. Resource scaling and actual physical evolution retain their own contracts, while the preserved sensitivity ranges remain assumptions rather than measured parameter distributions.

Table S36. Storage states, operational thresholds, reserve, and loss parameters.

Table S36		
**Parameter**	**Value**	**Unit**
storage.capacitance_f	270.0	F
storage.capacity_j	1949.4	J
storage.initial_j	1749.6	J
storage.brownout_j	843.75	J
storage.restart_j	1215.0	J
storage.reserve_j	5.0	J
storage.observation_uncertainty_j	0.0	J
storage.leakage_ref_a	2e-05	A
storage.leakage_ref_v	3.8	V
storage.leakage_multiplier	1.0	ss
storage.other_loss_w	0.0	W
storage.estimated_loss_w	0.001	W
controller.max_energy_age_s	1.0	s

S9.5 Inherited storage and incremental service evidence

Table S37 retains the inherited storage, leakage, service-cost, and radio-current quantities. Their evidential qualifications in **Hata! Başvuru kaynağı bulunamadı.** prevent nominal values from being mistaken for calibrated hardware constants. In particular, source sensing and processing ranges become declared midpoint increments above M0, while current-to-energy conversion uses an explicitly assumed 3.3 V radio abstraction.

The voltage- and temperature-dependent leakage law is a separate assumption, and its reference setting is not a fitted physical curve. These records qualify the manuscript's accounting: document-supported currents do not establish packet airtime or complete-board consumption. Retaining the

source/model boundary also avoids charging a total source energy and the common baseline as though they were independent expenditures.

Table S37. Inherited storage, service-energy, and radio-current quantities.

Table S37		
**Parameter**	**Value**	**Unit**
storage	{"capacitance_f":270,"vmax_v":3.8,"brownout_v":2.5,"restart_v":3.0}	F, V
leak_reference_current	2e-05	A
service_incremental_energy	{"sensing_j":0.012,"processing_j":0.11}	J/task
radio_current	{"tx_a":0.028,"rx_a":0.01}	A

S9.6 Synthetic environment and leakage settings

The environmental register defines synthetic forcing rather than reconstructed irradiance or temperature measurements. Table S38 retains the weather bins, daylight window, harvest peak, diurnal temperature, and leakage factors.

The 0.025 W harvest peak is below the source panel's nominal 1 W rating, but that comparison does not calibrate the adopted input. Leakage doubling and amplification are also declared sensitivities. These assumptions qualify the manuscript's environmental interpretation: shared exogenous conditions permit controlled policy comparisons while leaving physical parameter uncertainty and deployment-specific weather outside the demonstrated evidence. The future realization remains unavailable to the controller when it selects a plan.

Table S38. Synthetic weather, harvesting, temperature, and leakage settings.

Table S38		
**Parameter**	**Value**	**Unit**
weather_bin	300	s
weather_uniform_support	[0.3,1.0]	normalized context
harvest_peak	0.025	W
daylight	[6,18]	hour of day
diurnal_period	86400	s
temperature_nominal_mean	25	°C
temperature_amplitude	10	°C
temperature_phase	9	hour of day

Table S38		
**Parameter**	**Value**	**Unit**
hot_temperature_increment	20	°C
leakage_reference_temperature	25	°C
leakage_Q10	2	dimensionless per 10 °C
leakage_temperature_scale	10	°C
high_leak_multiplier	10	dimensionless

S9.7 Physical events and channel realization

Physical-event demand and background radio occupancy are separately generated. Table S39 retains event visibility, sparse/mixed rates, simultaneous bursts, channel occupancy, and random-loss settings. Coincident physical events retain separate identities; background traffic can affect congestion without becoming sensed local demand.

The fixed time-keyed channel construction avoids changing random-number consumption when policy execution differs. Its millisecond key is separate from the exact scheduling clock. These assumptions delimit the manuscript's paired communication experiment, rather than providing a calibrated propagation model or protocol-derived airtime. The same packet-loss parameter is linked to both directions without implying that their attempts occur at identical times.

Table S39. Physical events, background traffic, and channel realization parameters.

Table S39		
**Parameter**	**Value**	**Unit**
physical_event_duration	120	s
nominal_event_rate	0.0016666666666666668	events/s
mixed_event_rates	[0.0005555555555555556,0.03333333333333333]	events/s
mixed_regime_duration	43200	s
simultaneous_burst_size	3	events/burst
first_burst	21600	s
burst_interval	43200	s
background_nominal_rate	0.016666666666666666	packets/s

Table S39		
**Parameter**	**Value**	**Unit**
background_mixed_rates	[0.008333333333333333,0.08333333333333333]	packets/s
background_airtime	0.12	s/packet
uplink_random_loss	0.1	probability/attempt
downlink_random_loss	0.1	probability/attempt
channel_time_quantum	0.001	s
channel_hash_bits	64	bits

S9.8 Gateway observation and command timing

Gateway information is acquired on clocks distinct from node application. Table S40 retains outage timing, the default period, timeout, compute cadence, traffic window, and telemetry-age limit. **Hata! Başvuru kaynağı bulunamadı.** explains why each enters the declared experiment.

Traffic normalization uses successfully observed packet completions over the preceding window. Delivered SoC older than 7200 s, or absent telemetry, suppresses valid command generation. A received command is separately rechecked against its original reception age before later application. These distinctions qualify the manuscript's gateway adaptation: restoration of communication does not guarantee immediately fresh inputs or an immediately applied command. The fixed 900 s comparator and fallback share a number but represent different policy circumstances, and the outage remains a prescribed external interval.

Table S40. Gateway outage, observation, generation, and command-age settings.

Table S40		
**Parameter**	**Value**	**Unit**
gateway_outage	[43200,172800]	s since origin
fixed_and_default_period	900	s
command_timeout	1800	s
gateway_compute_period	300	s
gateway_compute_phase	-1	s relative to the 300 s tick
traffic_observation_window	3600	s
traffic_normalization_rate	0.016666666666666666	packets/s

Table S40		
**Parameter**	**Value**	**Unit**
maximum_delivered_SoC_age	7200	s

S9.9 Requested service and bounded queue

Requested work and stored observations have separate limits. Table S41 retains sensing/processing timing and the bounded queue, record size, uplink capacity, and command-packet size. The 60s service demand does not depend on the FLC report period, and processing still requires successful sensing.

The FIFO queue drops new records when full rather than silently increasing memory or removing the oldest record. Packet capacity limits a report batch, while the command-packet metadata size does not determine airtime through an unstated radio formula. These contracts make the manuscript's service and queue outcomes interpretable. They are timed-job and serialization assumptions, not MCU benchmarks or proof that every generated observation can be delivered within a finite horizon.

Table S41. Requested service, queue, observation, and packet-capacity settings.

Table S41		
**Parameter**	**Value**	**Unit**
sensing_period	60	s
sensing_duration	0.02	s/task
sensing_start_slack	0.01	s
sensing_completion_deadline	0.1	s from release
processing_duration	0.04	s/task
processing_completion_deadline	0.2	s from release
queue_capacity	1000	observations
record_size	10	bytes/observation
uplink_payload_capacity	250	bytes
downlink_packet_size	24	bytes

S9.10 Blind-copy transactions and receive windows

The communication profile schedules two blind copies without acknowledgements. Table S42 retains their durations, spacing, and node receive window. Uplink copies are consecutive, and the nominal

target refers to completion of the whole 0.24 s transaction. Downlink copies use separate 0.04 s airtimes within the receive window.

A second copy is sent irrespective of first-copy success. The node listens for the full configured window even if no valid command is produced or the gateway is unavailable. These assumptions qualify the manuscript's overhead interpretation: hidden delivery cannot alter local dequeue decisions, and two packets within one listening window do not imply two independent full-window energy charges. They also remain declared fixed airtimes rather than outputs of a protocol-specific link calculation.

Table S42. Blind-copy transaction and receive-window timing.

Table S42		
**Parameter**	**Value**	**Unit**
uplink_copies	2	copies/transaction
uplink_attempt_airtime	0.12	s/attempt
uplink_transaction_duration	0.24	s
downlink_copies	2	copies/valid command
downlink_attempt_airtime	0.04	s/attempt
downlink_attempt_spacing	0.05	s start-to-start
node_RX_window	0.1	s

S9.11 Node and gateway energy increments

Table S43 presents node radio increments, gateway baseline and communication costs, and paid controller overhead. The 3.3 V conversion is explicit because voltage is not supplied by the source current table. A receive-window increment is charged once, even though two downlink copies fit inside it.

These assumptions qualify the manuscript's energy interpretation. Increments are additional to the specified M0 boundary, gateway baseline is separate, and background-node energy is excluded. The resulting accounting is reproducible within the model but does not measure whole-network consumption or physical processor execution cost.

Table S43. Node, gateway, and controller energy parameters.

Table S43		
**Parameter**	**Value**	**Unit**
radio_voltage	3.3	V

Table S43		
**Parameter**	**Value**	**Unit**
node_RX_increment	0.0033	J/window
node_TX_increment	0.022176	J/transaction
gateway_baseline	1	W
gateway_inference_increment	0.001	J/inference
gateway_TX_power	0.5	W
gateway_RX_increment_power	0.05	W
controller_duration	0.001	s/decision attempt
controller_increment	1e-05	J/decision attempt
controller_budget	100	J/horizon

S9.12 Numerical criteria and causal execution semantics

Table S44 preserves numerical criteria and execution assumptions. The retained 10^{-8} s runner and 10^{-9} s core tolerance statements do not override the current exact clock's zero admission tolerance. Likewise, the historical transaction-completion knowledge statement is superseded by current first-successful-copy reception semantics.

The ledger criteria assess numerical readdition, while process count affects execution rather than experimental replication. The frozen core configuration is now recovered, but original source labels and ranges remain visible. These distinctions support the manuscript's reproducibility boundary without treating an older register as proof of current runtime semantics or a numerical tolerance as physical model validation.

Table S44. Retained numerical and causal-execution register.

Table S44		
**Parameter**	**Value**	**Unit**
numerical_runner_tolerance	1e-08	s
ledger_absolute_tolerance	1e-07	J
ledger_relative_tolerance	1e-10	fraction of throughput
loop_bound	20000	decisions/ru n

Table S44		
**Parameter**	**Value**	**Unit**
parallel_workers	4	processes
core_configuration_reference	revision/method/METHOD_CONFIG.json	artifact path
radio_observation_semantics	Background counts include only packets for which the gateway is available at both packet start and completion. Own telemetry enters gateway knowledge at transaction completion; actual first-copy arrival ages are reported separately.	causal protocol definition

S10 72 h Evaluation

S10.1 Four-arm energy and sensing

The campaign comprises 216 simulations of 72 h: four arms in six scenarios with eight matched seeds, plus three mixed-workload input removals. Fixed 900 s reporting defines A/B; the FLC defines C/D. A/C use the lowest-residence-power feasible EARLIEST baseline, whereas B/D minimize estimated total energy. Each arm evolves its own storage, queues, service, and received information.

Table S45 and Figure S12 retain all four-arm scenario outcomes. Every historical run reaches modeled brownout. Under mixed traffic, D completes 51.17% of requested sensing and delivers 21.69% of physical events, compared with 53.56% and 59.30% for A/B. The 51.17% sensing endpoint is distinct from due-service completion in the current campaign. Mixed D's node load is 2249.87 J, close to A/B's 2249.79 J despite markedly different delivery. These results preserve the historical evidence relevant to the manuscript's energy and service discussion. They do not establish a general benefit of combined adaptation, equal-work savings, or outcomes of the current protocol. Descriptive seed intervals retain their scenario-specific interpretation without multiplicity adjustment.

Table S45. Node load, sensing completion, and physical delivery for all 24 scenario–arm cells; eight-seed means and descriptive 95% t intervals.

Table S45			
**Scenario / arm**	**Node load J**	**Sensing completed %**	**Events delivered %**
nominal / A	2249.79 [2236.75, 2262.82]	53.61 [53.22, 54.00]	52.39 [51.33, 53.45]
nominal / B	2249.79 [2236.75, 2262.82]	53.61 [53.22, 54.00]	52.39 [51.33, 53.45]
nominal / C	2250.04 [2237.00, 2263.09]	47.67 [40.37, 54.97]	46.97 [39.43, 54.51]
nominal / D	2249.98 [2236.92, 2263.05]	47.71 [41.68, 53.74]	46.59 [40.55, 52.63]
mixed / A	2249.79 [2236.75, 2262.82]	53.56 [53.17, 53.95]	59.30 [58.31, 60.29]

Table S45			
**Scenario / arm**	**Node load J**	**Sensing completed %**	**Events delivered %**
mixed / B	2249.79 [2236.75, 2262.82]	53.56 [53.17, 53.95]	59.30 [58.31, 60.29]
mixed / C	2238.38 [2209.94, 2266.83]	49.96 [46.94, 52.97]	21.12 [19.43, 22.81]
mixed / D	2249.87 [2236.82, 2262.92]	51.17 [50.18, 52.16]	21.69 [21.08, 22.30]
outage / A	2249.79 [2236.75, 2262.82]	53.61 [53.22, 54.00]	25.48 [23.88, 27.08]
outage / B	2249.79 [2236.75, 2262.82]	53.61 [53.22, 54.00]	25.48 [23.88, 27.08]
outage / C	2249.96 [2236.89, 2263.03]	49.60 [43.35, 55.85]	23.43 [18.49, 28.38]
outage / D	2249.97 [2236.90, 2263.03]	49.49 [43.27, 55.71]	23.05 [18.15, 27.95]
hot / A	2212.80 [2199.80, 2225.80]	52.70 [52.37, 53.02]	51.61 [50.61, 52.61]
hot / B	2212.80 [2199.80, 2225.80]	52.70 [52.37, 53.02]	51.61 [50.61, 52.61]
hot / C	2213.32 [2200.19, 2226.45]	48.84 [42.61, 55.07]	47.48 [40.17, 54.80]
hot / D	2213.65 [2200.27, 2227.02]	46.08 [39.25, 52.91]	45.11 [38.11, 52.11]
high_leak / A	2139.63 [2126.70, 2152.56]	50.95 [50.61, 51.30]	49.80 [48.83, 50.78]
high_leak / B	2139.63 [2126.70, 2152.56]	50.95 [50.61, 51.30]	49.80 [48.83, 50.78]
high_leak / C	2141.85 [2128.54, 2155.17]	44.16 [36.08, 52.25]	43.17 [35.30, 51.04]
high_leak / D	2141.20 [2127.65, 2154.75]	45.87 [40.17, 51.57]	45.42 [39.98, 50.85]
no_harvest / A	903.20 [903.20, 903.20]	21.53 [21.53, 21.53]	19.99 [19.10, 20.88]
no_harvest / B	903.20 [903.20, 903.20]	21.53 [21.53, 21.53]	19.99 [19.10, 20.88]
no_harvest / C	903.60 [902.86, 904.33]	18.52 [12.49, 24.56]	17.55 [11.66, 23.44]
no_harvest / D	903.55 [902.80, 904.30]	18.78 [12.67, 24.90]	17.69 [11.79, 23.60]

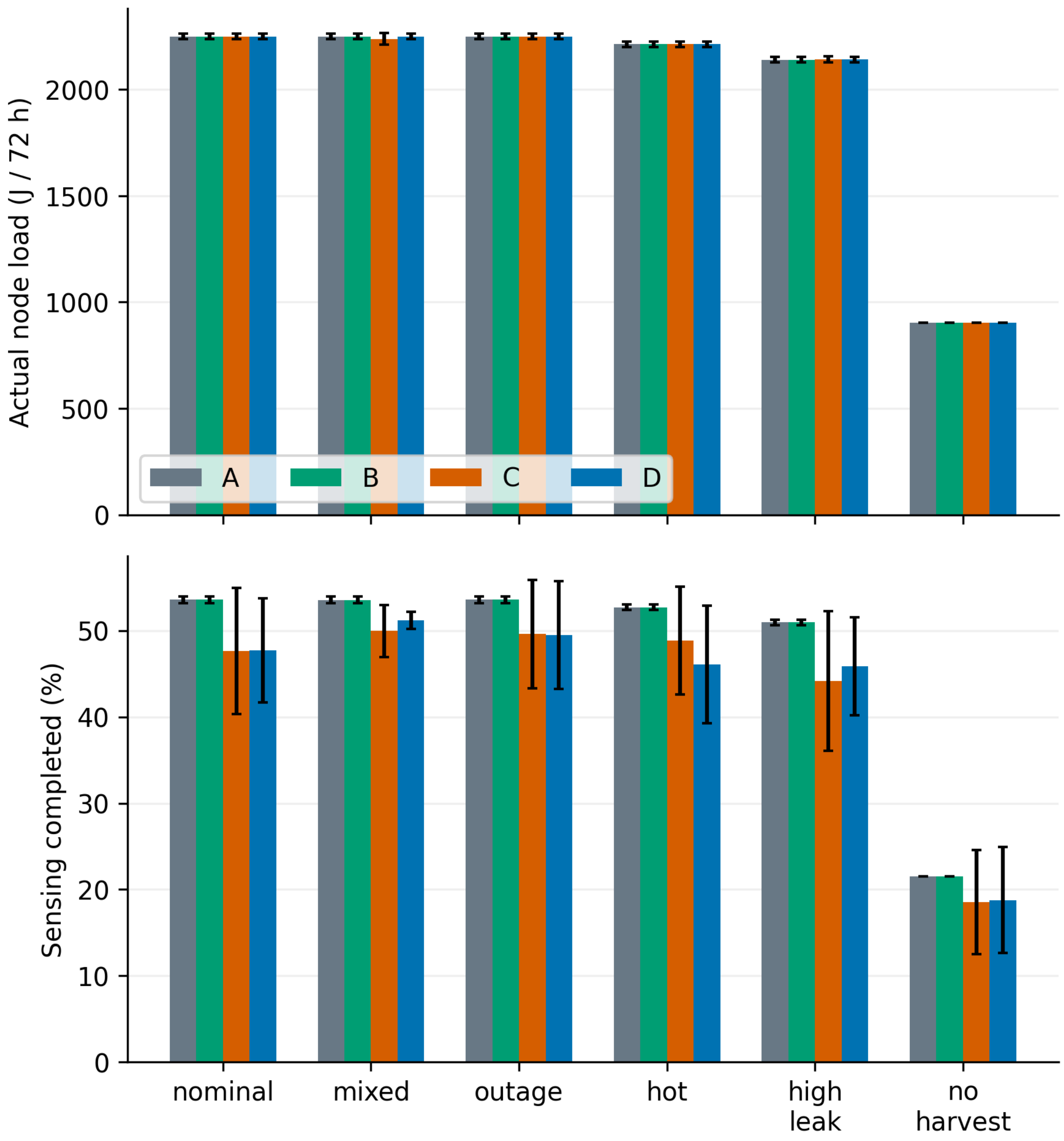

Figure S12. Four-arm load and sensing outcomes across six scenarios, with descriptive 95% t intervals over eight matched seeds.

S10.2 Selector interaction

The historical four-arm design allows selector effects to be compared under fixed and adaptive reporting. Table S46 reports seed-paired A−B, C−D, and the interaction (C−D)−(A−B), in joules. A positive selector contrast denotes lower realized load in the estimated-total-energy arm. A positive interaction denotes a larger such effect under FLC reporting than under the fixed period.

The mixed-workload interaction is −11.4865 J, with a descriptive 95% t interval of [−38.6775, 15.7045] J. Its negative mean and interval spanning zero provide no consistent improvement. A−B is printed as zero in five scenarios; the small hot-scenario mean is 0.0003 J with an interval spanning zero. C−D also

changes sign across scenarios, and every displayed interval includes zero. These neutral and adverse findings are part of the historical evidence behind the manuscript's selector discussion. They concern the replacement selector used in that campaign, not a sensitivity sweep of a removed weighting coefficient. Because reporting policies realize different service, the contrasts are not automatically energy savings for equal completed work. Internal arms also provide no comparison against independently reimplemented external adaptive methods, and their estimates cannot be substituted for the successor cohort.

Table S46. Seed-paired selector effects and their interaction across six scenarios; brackets give descriptive 95% t intervals over eight seeds.

Table S46			
**Scenario**	**EA – EB**	**EC – ED**	**Interaction**
nominal	0.0000 [0.0000, 0.0000]	0.0594 [-0.0697, 0.1885]	0.0594 [-0.0697, 0.1885]
mixed	0.0000 [0.0000, 0.0000]	-11.4865 [-38.6775, 15.7045]	-11.4865 [-38.6775, 15.7045]
outage	0.0000 [0.0000, 0.0000]	-0.0081 [-0.0287, 0.0126]	-0.0081 [-0.0287, 0.0126]
hot	0.0003 [-0.0004, 0.0011]	-0.3264 [-0.7035, 0.0506]	-0.3268 [-0.7041, 0.0506]
high_leak	0.0000 [0.0000, 0.0000]	0.6519 [-1.6678, 2.9716]	0.6519 [-1.6678, 2.9716]
no_harvest	0.0000 [0.0000, 0.0000]	0.0498 [-0.0313, 0.1309]	0.0498 [-0.0313, 0.1309]

S10.3 Outage and restart behavior

The outage disables gateway reception and transmission between hours 12 and 48 while local storage and service continue to evolve. Figure S13 shows the predesignated seed 1101 across voltage, nominal period, and queued observations. Its policy-dependent histories complement the arm-D summaries in Table S47, rather than defining an average trajectory.

For D under outage, mean brownouts and restarts are 3.12 and 2.12, respectively, and all eight first-brownout times are observed. Across the six D scenarios, the no-harvest case records one brownout and no restart, whereas high leakage raises mean leakage expenditure to 122.790 J. These outcomes retain depletion and interrupted operation rather than implying guaranteed survival under fallback. Following gateway return, accepted-command reception occurs after a mean 14.86 h and first successful report delivery after 14.84 h. Reception is distinct from later calendar application, and the recovery report can be empty. This historical evidence qualifies the manuscript's outage interpretation: restored infrastructure does not immediately restore information or service. The adopted storage law is a model assumption, not physical calibration, and one selected trace cannot establish a population lifetime.

Table S47. Arm-D leakage, brownout, restart, and first-brownout censoring by scenario; means and descriptive 95% t intervals over eight seeds.

Table S47				
**Scenario**	**Leakage J**	**Brownouts**	**Restarts**	**Censored first brownout**
nominal	12.372 [12.142, 12.603]	3.12 [2.83, 3.42]	2.12 [1.83, 2.42]	0/8
mixed	12.465 [12.437, 12.492]	3.00 [3.00, 3.00]	2.00 [2.00, 2.00]	0/8
outage	12.382 [12.151, 12.613]	3.12 [2.83, 3.42]	2.12 [1.83, 2.42]	0/8
hot	49.288 [48.353, 50.224]	3.12 [2.83, 3.42]	2.12 [1.83, 2.42]	0/8
high_leak	122.790 [120.675, 124.906]	3.12 [2.83, 3.42]	2.12 [1.83, 2.42]	0/8
no_harvest	10.205 [9.991, 10.420]	1.00 [1.00, 1.00]	0.00 [0.00, 0.00]	0/8

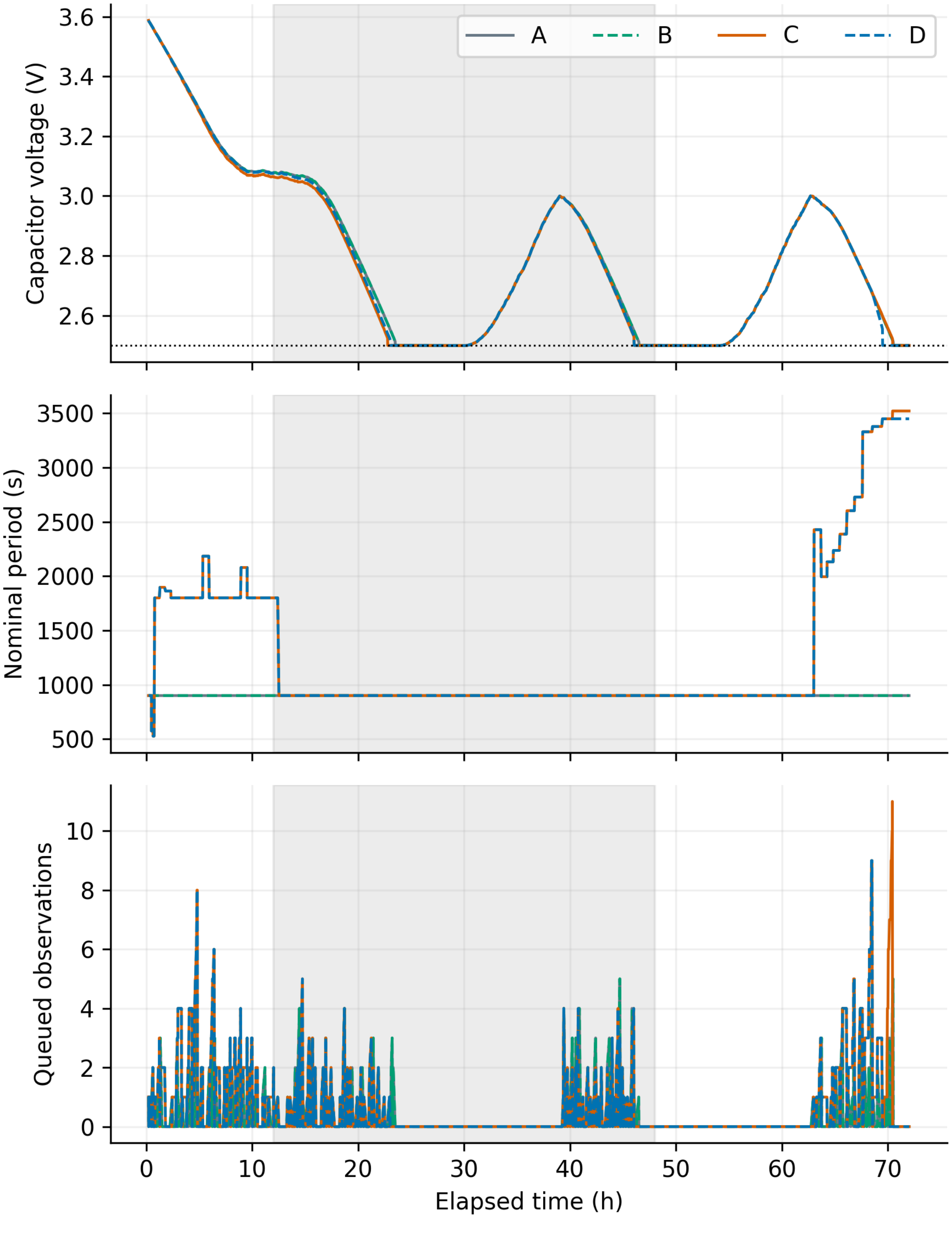

Figure S13. Seed 1101 voltage, nominal period, and queue histories; shading marks the 12–48 h outage and the dotted line marks 2.5 V.

S10.4 Communication boundary

Historical communication costs depend on whether the accounting boundary includes node listening, downlink transmission, or gateway activity. Table S48 reports arm D alone over 72 h. Nominal downlink airtime is 37.360 s, the node RX increment is 1.357 J, and the gateway increment is 20.261 J. Under outage, the respective means are 18.840 s, 1.406 J, and 10.258 J. Reduced downlink airtime therefore does not eliminate the node's separately scheduled receive obligation.

The no-ACK profile sends two predetermined copies of each report and valid command. A completed report removes its local batch irrespective of hidden channel success. Background occupancy and random packet errors are exogenous; gateway load information includes actually observed packets. Periodic listening is shared by all four arms, so its gross cost is not an adaptive-minus-fixed decoding expense. Gateway incremental energy includes inference and radio activity, while the declared 1 W baseline is separate. These distinctions support the manuscript's historical communication discussion without identifying packet counts with energy or successful application. Copy loss, reception, and command application remain different records. The six-row D table neither aggregates all 216 runs nor calibrates a multi-node radio network, and it remains separate from current communication results.

Table S48. Arm-D downlink airtime, node RX increment, and gateway incremental energy by scenario; means and descriptive 95% t intervals over eight seeds.

Table S48			
**Scenario**	**Downlink airtime s**	**Node RX increment J**	**Gateway increment J**
nominal	37.360 [33.537, 41.183]	1.357 [1.186, 1.528]	20.261 [18.244, 22.278]
mixed	39.590 [38.682, 40.498]	1.455 [1.427, 1.482]	21.596 [21.140, 22.053]
outage	18.840 [16.220, 21.460]	1.406 [1.229, 1.583]	10.258 [8.868, 11.649]
hot	36.170 [31.993, 40.347]	1.313 [1.119, 1.508]	19.643 [17.452, 21.835]
high_leak	36.230 [33.010, 39.450]	1.306 [1.141, 1.470]	19.663 [17.960, 21.366]
no_harvest	14.620 [10.409, 18.831]	0.534 [0.360, 0.707]	8.477 [6.277, 10.677]

S11 Independent 24 h Network Evaluation

S11.1 Finite static diagnostics in the 24 h model

The static diagnostic associated with the 24 h model evaluates 343 deterministic input triples, with 294 adjacent edges per axis and output stage. Table S49 distinguishes nominal centroid and Low-SoC-protected output before damping, quantization, or transport. Both sampled ranges are 445.104–3200 s within the configured 60–3600 s universe; these sampled extrema are not continuous-domain bounds.

The intended period directions are nonincreasing with SoC, nondecreasing with load, and nonincreasing with improving weather context. Protected output violates them on eight, twelve, and 28 edges, respectively, using a 10^{-9} s reporting tolerance. At the central triple, both stages give 1800 s; at zero inputs, protection raises the nominal 1800 s output to 3200 s. Figure S14 illustrates three central slices. These historical diagnostics qualify the manuscript's controller-direction interpretation without

supporting global monotonicity or dynamic stability. They are deterministic evaluations, so neither 30-trace replication nor bootstrap uncertainty applies. The image's original complete grid-generation identity is not independently established by the retained source.

Table S49. All adjacent-edge directions for nominal and protected outputs on the 343-point static grid; the zero category uses a 10^{-9} s tolerance.

Table S49				
**Increasing axis**	**Stage**	**Increase**	**Decrease**	**Zero (1e–9 s)**
SoC	Nominal	10	99	185
SoC	Protected	8	134	152
Load	Nominal	68	18	208
Load	Protected	54	12	228
Weather	Nominal	39	91	164
Weather	Protected	28	77	189

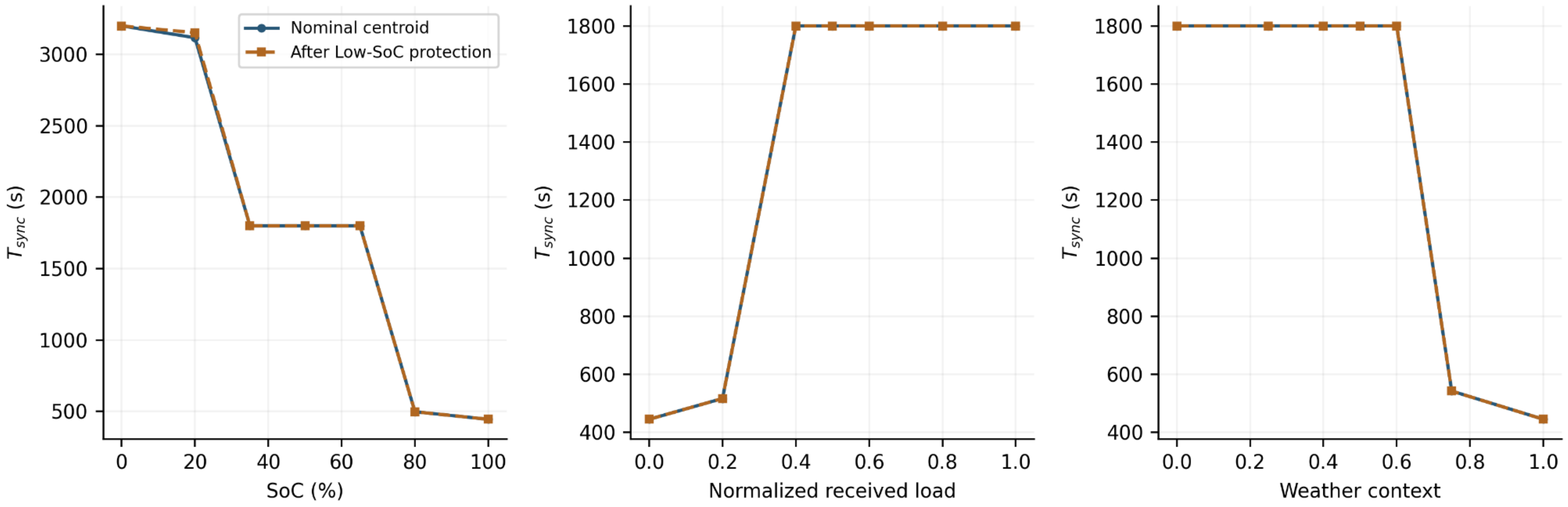

Static grid slices: remaining inputs fixed at SoC=50%, load=0.5, weather=0.5. No damping, dissemination or node-mode command is implied.

Figure S14. Central SoC, load, and weather slices compare nominal centroid and Low-SoC protection.

S11.2 Nominal energy and physical-event delivery

The separate 24 h evaluation compares HA-PM with fixed periods of 300, 600, 900, 1800, and 3600 s using matched exogenous traces. Figure S15 reports gross device energy together with physical-event delivery. HA-PM consumes 1040.718 J/node and delivers 37.700% of eligible events. Device energy includes realized transitions, active processing and uplink service, receive windows, and steady-state residence.

All five paired contrasts appear in Table S50. Relative to fixed 900 s, HA-PM uses 79.995 J/node more and delivers 1.361 percentage points less; the fixed policy has favorable means on both primary outcomes. Relative to fixed 1800 s, HA-PM instead combines 338.470 J/node more energy with 7.270 percentage points more delivery. Against fixed 300 s, lower energy accompanies a small delivery disadvantage. These comparisons retain the favorable, neutral, and unfavorable service context needed to assess the manuscript's historical energy claims. Different completed work prevents interpreting a lower energy total as service-equivalent savings. The independent unit is the entire network trace: 30 paired traces support pointwise exploratory bootstrap intervals, without multiplicity adjustment or an inference of 3000 independent node experiments.

Table S50. All five nominal HA-PM-minus-fixed contrasts in energy and delivery; 30 defined trace pairs with pointwise exploratory 95% bootstrap intervals.

Table S50			
**HA-PM minus fixed**	**Δ energy (J/node)**	**Δ delivery (pp)**	**Defined pairs**
300s	-262.468 [-263.392, -261.560]	-0.351 [-0.453, -0.249]	30
600s	6.052 [4.997, 7.195]	0.393 [0.328, 0.454]	30
900s	79.995 [79.111, 80.881]	-1.361 [-1.434, -1.283]	30
1800s	338.470 [337.258, 339.753]	7.270 [7.200, 7.333]	30
3600s	457.678 [456.522, 458.735]	10.866 [10.788, 10.949]	30

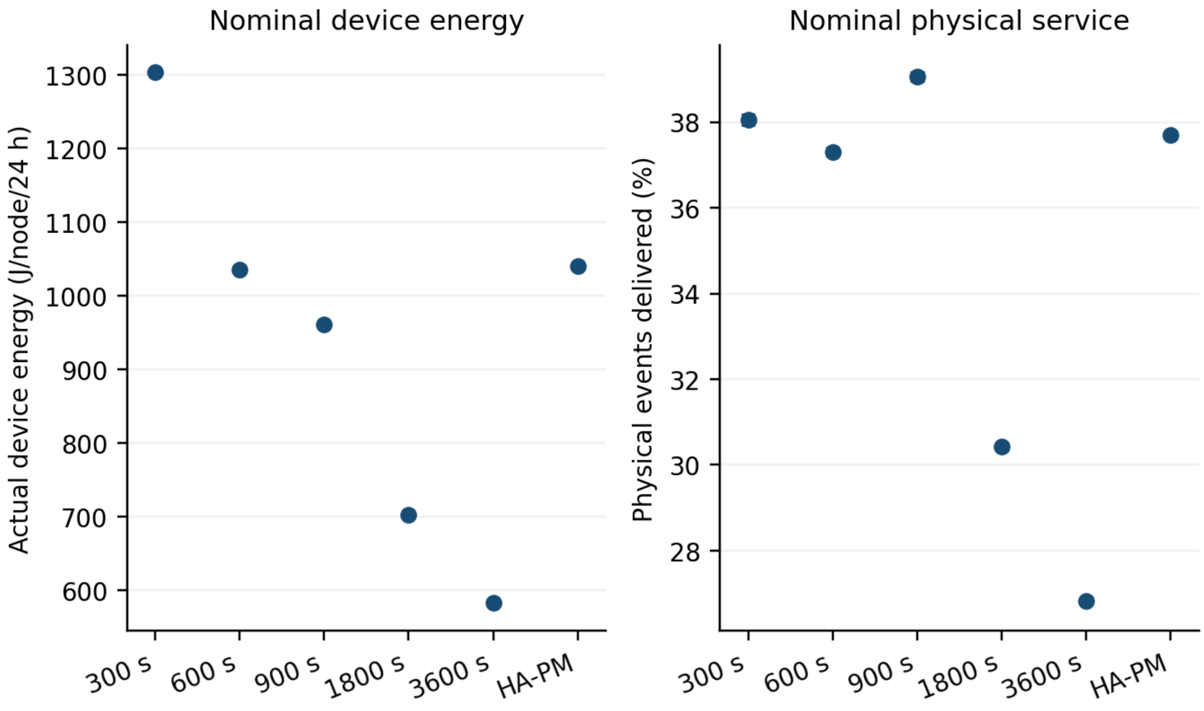

Figure S15. Nominal energy and physical delivery for six policies in the 24 h model; error bars show pointwise exploratory 95% trace-bootstrap intervals.

S11.3 Complete physical-event outcome partition

A delivery percentage is interpretable only with its complete physical-event denominator. Figure S16 partitions every eligible node–event pair in the historical 24 h model into delivered, missed, lost, or horizon-pending outcomes. The displayed categories distinguish physical sensing misses, queue overflow, energy-related misses and record loss, radio and gateway-outage loss, mixed misses, and pending events or records. Events after a node's first cutoff remain eligible rather than disappearing from the evaluation.

A physical event produces an observation only when the sensing state and available energy permit detection. Detected observations then enter the FIFO service path; an event during M19 residence does not automatically wake the node. Consequently, failure to observe a physical event and failure to deliver an existing record identify different stages. The nominal plot shows substantial sensing misses under every policy and an additional energy-miss contribution for fixed 300 s. This partition explains why energy expenditure or a reporting schedule alone cannot establish the physical service claimed in the manuscript. Fractions are averaged with equal weight across 30 network traces. They describe event outcomes, not time occupancy, independent node success probabilities, or measured radio reliability; the denominator remains the same eligible physical population within each matched comparison.

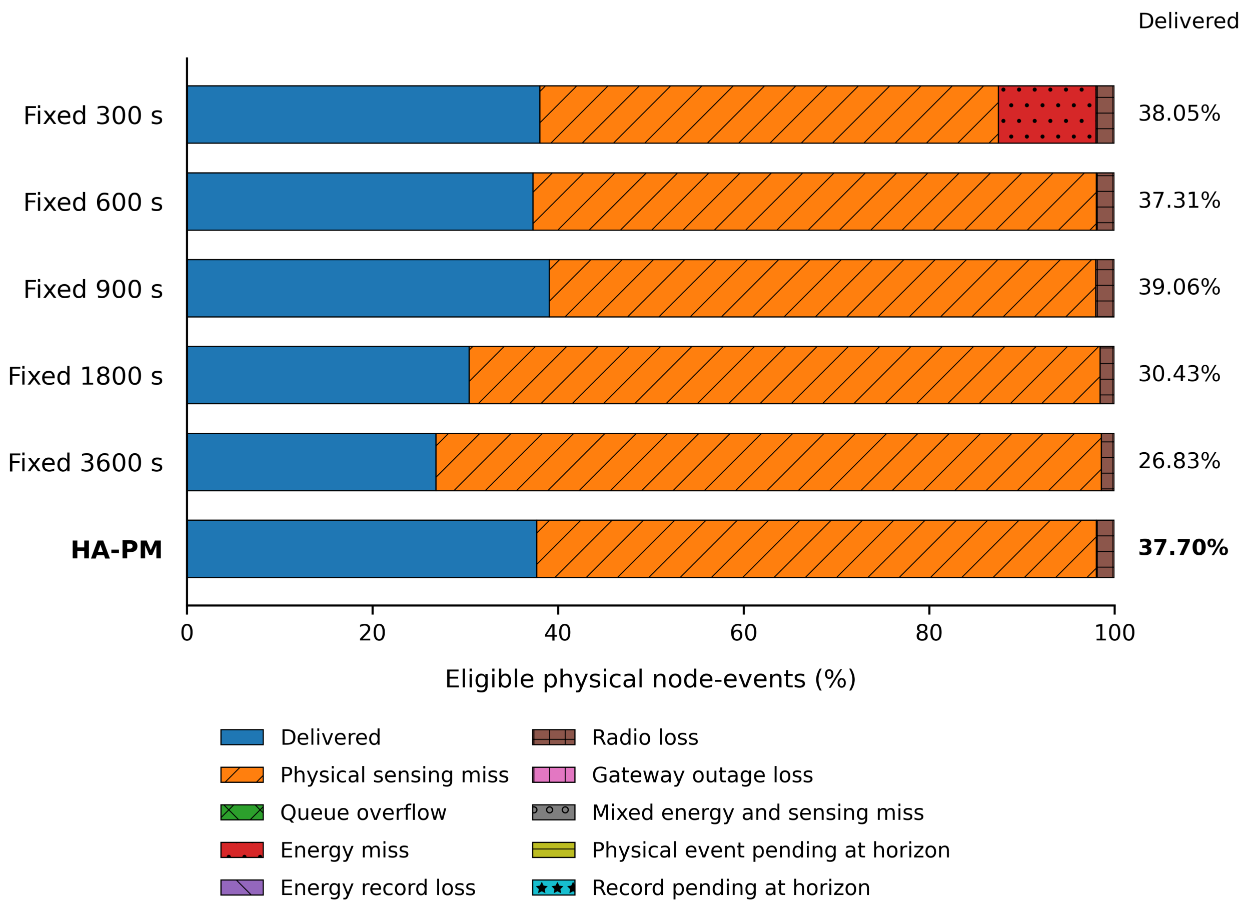

Figure S16. Complete nominal physical-event outcome partition for six policies; equal-weight means of 30 trace fractions retain post-cutoff and horizon-pending events.

S11.4 Complete node-time occupancy

Whole-horizon state occupancy describes where node time is spent, including time after operation stops. In the historical 24 h model, the denominator is 100 nodes multiplied by 86400 s for every policy. The displayed occupancy equation divides accumulated state duration by this common horizon. Figure S17 retains active residence, each candidate steady mode, entry/exit transitions, and absorbing time after the first cutoff.

$$R_i = \left(\frac{T_i}{NH}\right) \times 100\% \quad (6)$$

Fixed 300 s has a visible post-cutoff contribution, whereas the other nominal policies have no observed cutoff within the horizon. Excluding stopped-node time would renormalize the remaining states and obscure this difference. Transition residence also remains separate from steady sleep, because the model assigns distinct transition power and sensing availability. The large M18 and M19 contributions consequently describe actual modeled residence rather than the synchronization period acting as a unique mode command. Event fractions and node-time fractions answer different questions and cannot be added or substituted. Values are equal-weight means of complete trace fractions, not pooled independent mode observations. Occupancy alone neither establishes energy savings under equal service nor extrapolates operational lifetime beyond 24 h.

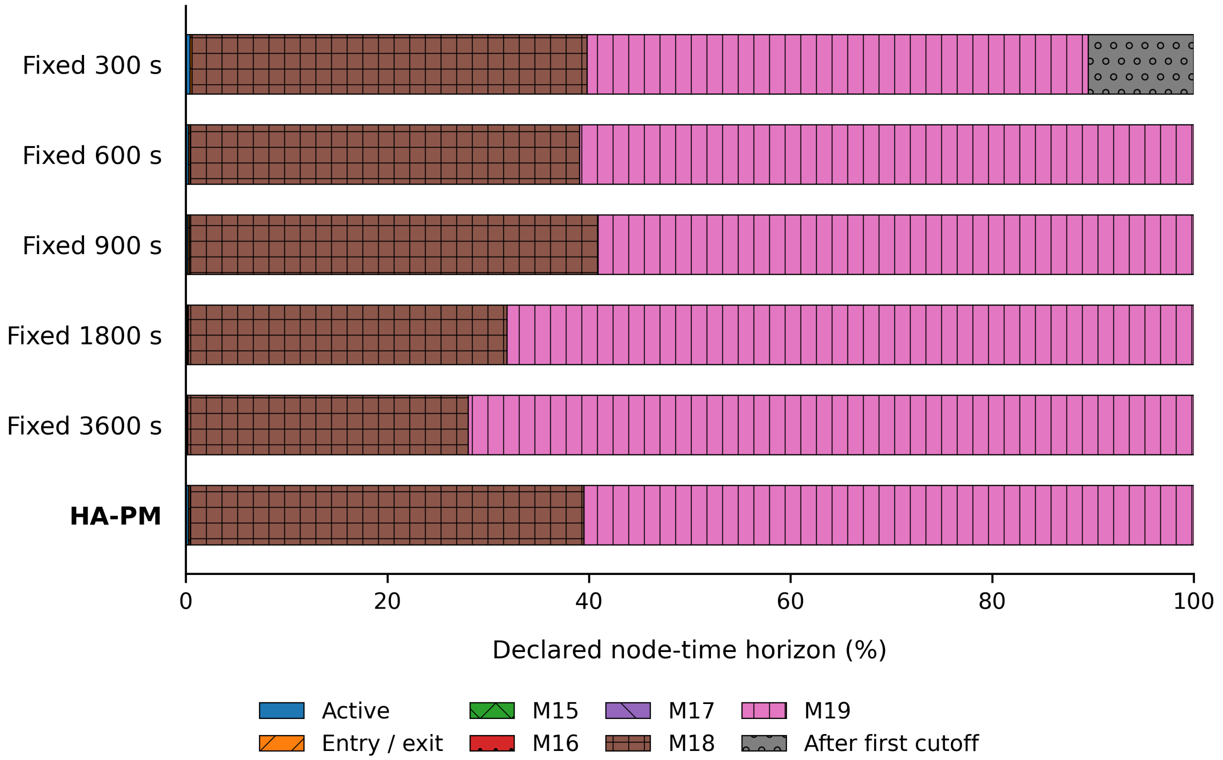

Figure S17. Complete nominal node-time partition across active residence, steady modes, transitions, and stopped-node time; means of 30 whole-horizon trace fractions.

S11.5 Stress-family outcomes

The historical network evaluation applies four declared stress families: weather degradation, gateway outage, burst traffic, and their combination. Weather falls to 0.2 during hours 6–12 and scales physical net harvesting. Gateway reception and downlink service are unavailable during hours 8–14, while the physical event rate increases during hours 10–12. Received network load remains an observed gateway input rather than being replaced by that physical rate.

Figure S18 retains energy and physical delivery for HA-PM and fixed 1800 s under every family. In the combined case, HA-PM consumes 907.935 J/node with 21.030% delivery; fixed 1800 s consumes 672.975 J/node with 17.165% delivery. Higher HA-PM delivery therefore entails higher modeled expenditure. The pointwise cell intervals summarize 30 independent traces and are not intervals for a paired difference. All node histories in these four stress families are right-censored at 24 h for both policies, so their uncensored lifetimes cannot be ranked from this horizon. These historical outcomes bound the manuscript's disturbance interpretation: continued operation does not imply complete event delivery, and an energy comparison must retain its accompanying service outcome.

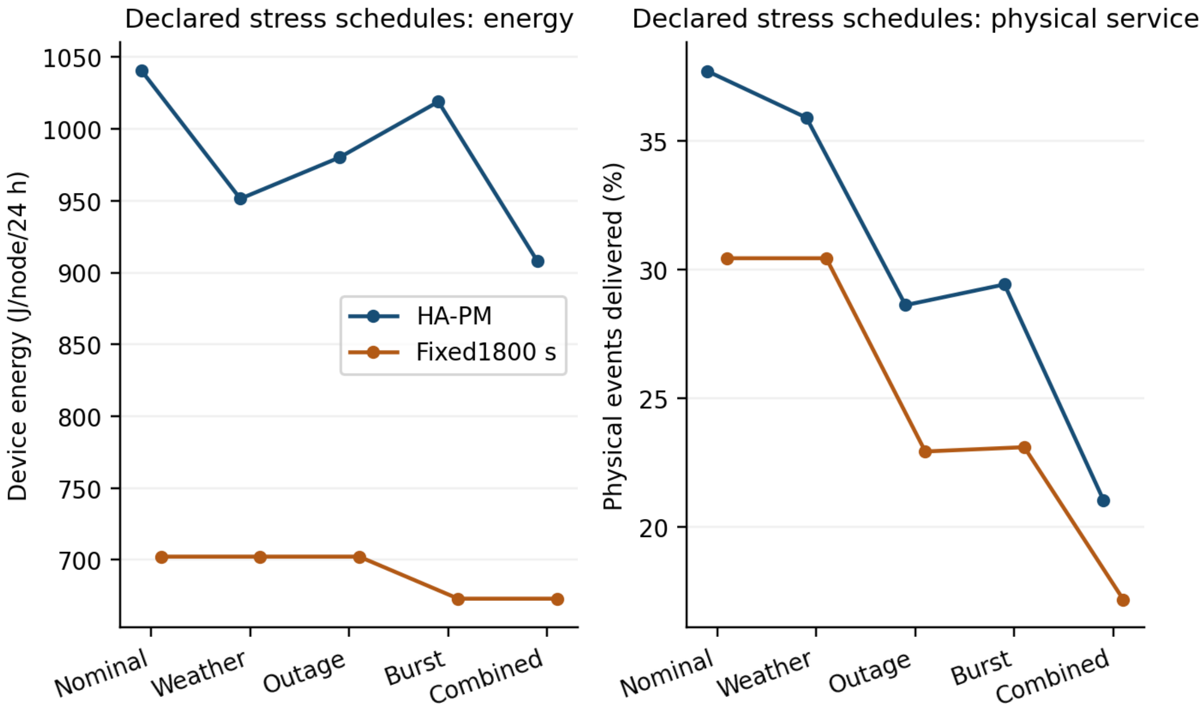

Figure S18. Joint energy and physical-delivery outcomes for weather, outage, burst, and combined stresses; pointwise exploratory 95% intervals across 30 traces.

S11.6 Command transport and receive/fallback diagnostics

The displayed command lifecycle separates a gateway calculation from a node's applied interval. Figure S19 reports generated, transmitted, received, validated, and applied counts in the historical 24 h evaluation. Generated commands greatly outnumber transmitted commands across the nominal policies; successful dissemination therefore cannot be assumed from controller evaluation alone. Equal-weight trace means retain each stage without treating their differences as a collision-rate estimate.

Figure S20 complements these counts with actual node receive-window energy, effective interval, and cache-timeout fallback. A completed heartbeat attempt opens local reception even when that heartbeat is lost; a gateway downlink additionally requires successful heartbeat receipt. Receive cost therefore need not vanish when no new command arrives. Effective intervals reflect actual node residence and may include initial default and timeout fallback, whose causes remain distinct. The figure's fallback percentage uses observed alive node time, unlike whole-horizon occupancy. These diagnostics clarify the historical dissemination mechanism relevant to the manuscript. They do not measure gateway transmission energy, end-to-end delivery guarantees, or hardware latency. Nor do application counts reconstruct an unrecorded continuous FLC trajectory. The independent sampling unit remains the complete network trace, with 30 traces underlying the reported nominal summaries.

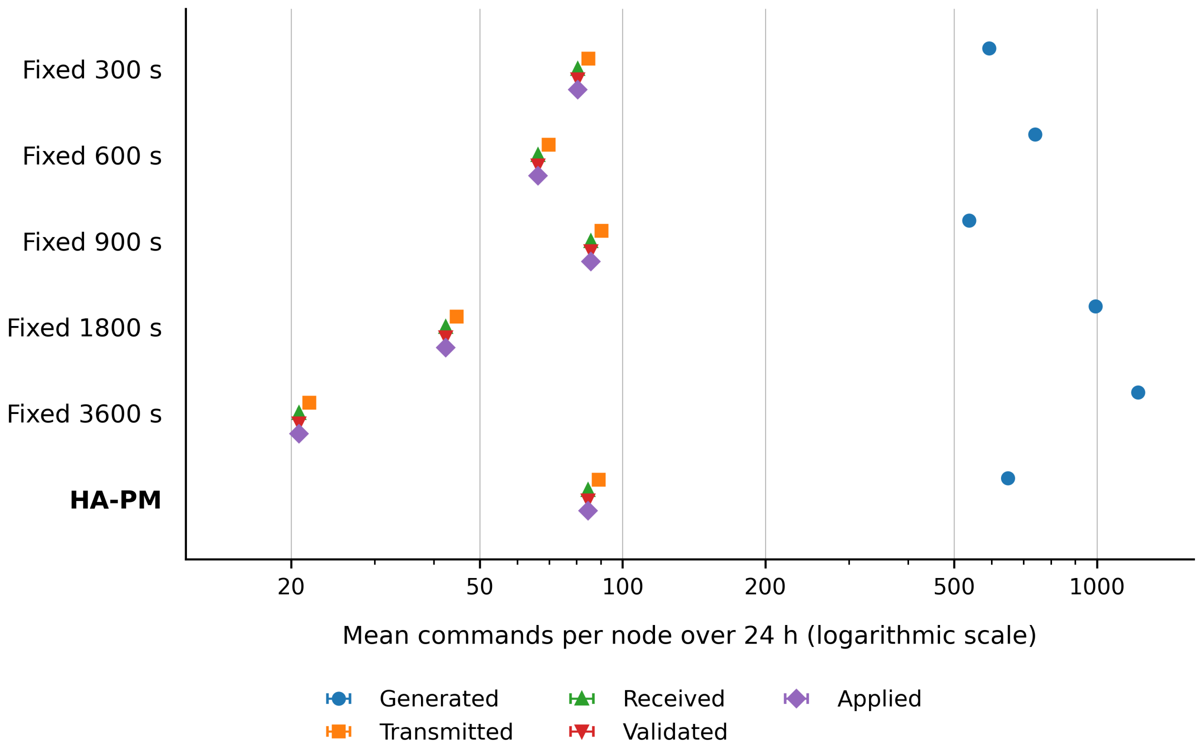

Figure S19. Different stages of the downlink command lifecycle. Nominal command counts per node for all six policies; means over 30 complete network traces.

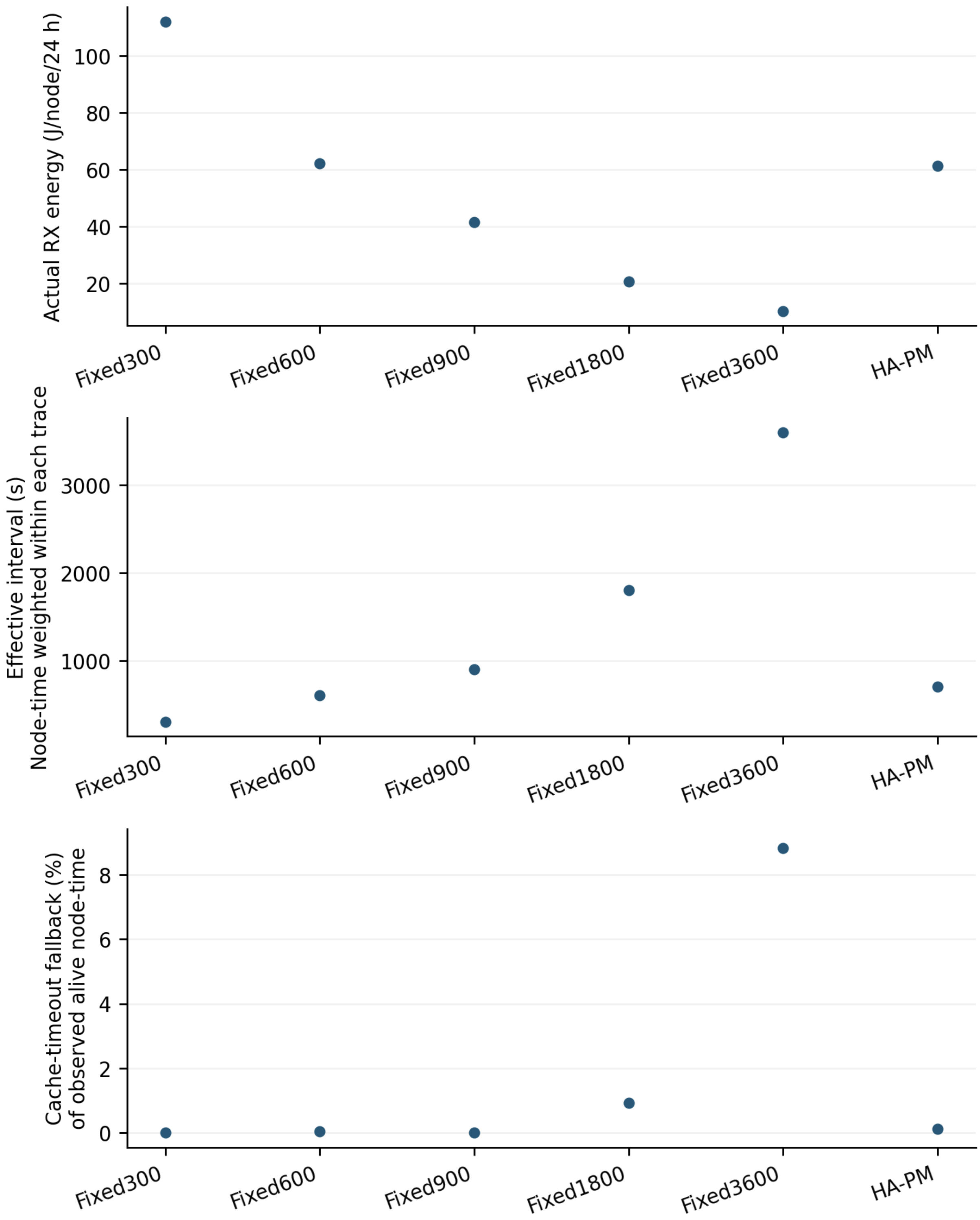

Figure S20. Nominal node RX energy, effective interval, and cache-timeout fallback across six policies; pointwise exploratory 95% trace intervals.

$$(S_{SoC,k}{}^{gw}, N_{load}{}^{gw}, W_{cond}{}^{gw}) \rightarrow T_{sync,k}{}^{generated} \rightarrow T_{sync,k}{}^{applied}$$

S11.7 Predesignated combined-stress node histories

Individual histories show how a declared disturbance interacts with a node's realized service and information state. Figure S21 retains the prospectively selected seed 1, nodes 0 and 99, under combined stress in the historical 24 h model. Voltage is shown at recorded energy-episode boundaries, together with HA-PM and fixed-1800 s interval histories. The shaded interval identifies the gateway outage from hour 8 to hour 14.

Both illustrated HA-PM voltage histories end below their fixed-period counterparts while remaining above the 2.5 V cutoff. The adaptive intervals also depart from the fixed schedule and display timeout-fallback spans. These observations provide concrete examples of the higher-energy, higher-delivery trade-off in the aggregate combined-stress comparison, but do not quantify an average network response. Voltage markers are recorded endpoints rather than a continuously measured physical waveform, and interval histories terminate at first cutoff rather than extending a disabled node's operation. This display supports the manuscript's historical explanation of command and storage dynamics. The two preselected nodes do not constitute additional independent traces, an aging experiment, or a global stability test. Comparative conclusions instead depend on the complete 30-trace summaries and their explicitly defined service and energy boundaries.

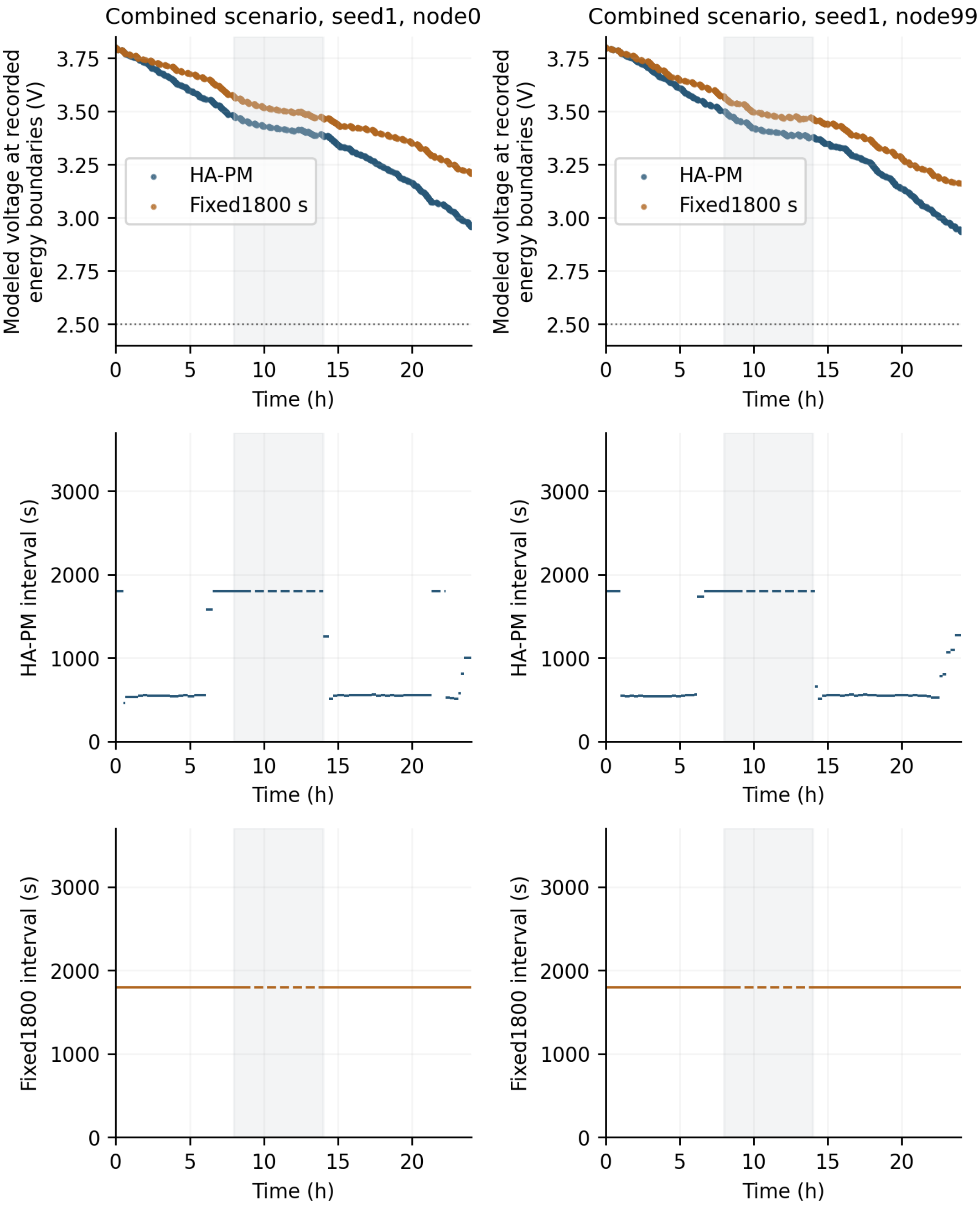

Figure S21. Six panels show recorded voltage and HA-PM/fixed-period interval histories for seed 1, nodes 0 and 99; dashed spans indicate cache-timeout fallback.

S11.8 Control and transition sensitivity

The historical 24 h sensitivity study changes one declared control or transition factor at a time while retaining both energy and physical delivery. Figure S22 covers local weight, strict risk threshold, transition-latency multiplier, and damping. These factors affect a model that includes actual event

detection, active service, and transition consumption; it is distinct from a conditional cycle-energy calculation.

Across the tested local weights, lower expenditure can accompany lower delivery, particularly for fixed 1800 s. Changing the risk threshold gives visually unchanged nominal outcomes over the plotted settings, a neutral finding retained alongside stronger sensitivities. Increasing transition latency raises modeled energy, while the adaptive delivery curve declines. The two damping settings have closely spaced means with overlapping displayed intervals. These patterns do not identify a universally optimal parameter or support retuning after inspection. Rather, they delimit how the historical model responds to the selected perturbations relevant to the manuscript's robustness discussion. Each cell is based on 30 whole-network traces; pointwise exploratory intervals have no multiplicity adjustment. Raster positions support qualitative comparisons, while exact estimates remain those of the retained source analyses. These historical local-policy settings are not parameters of the current replacement-selector campaign.

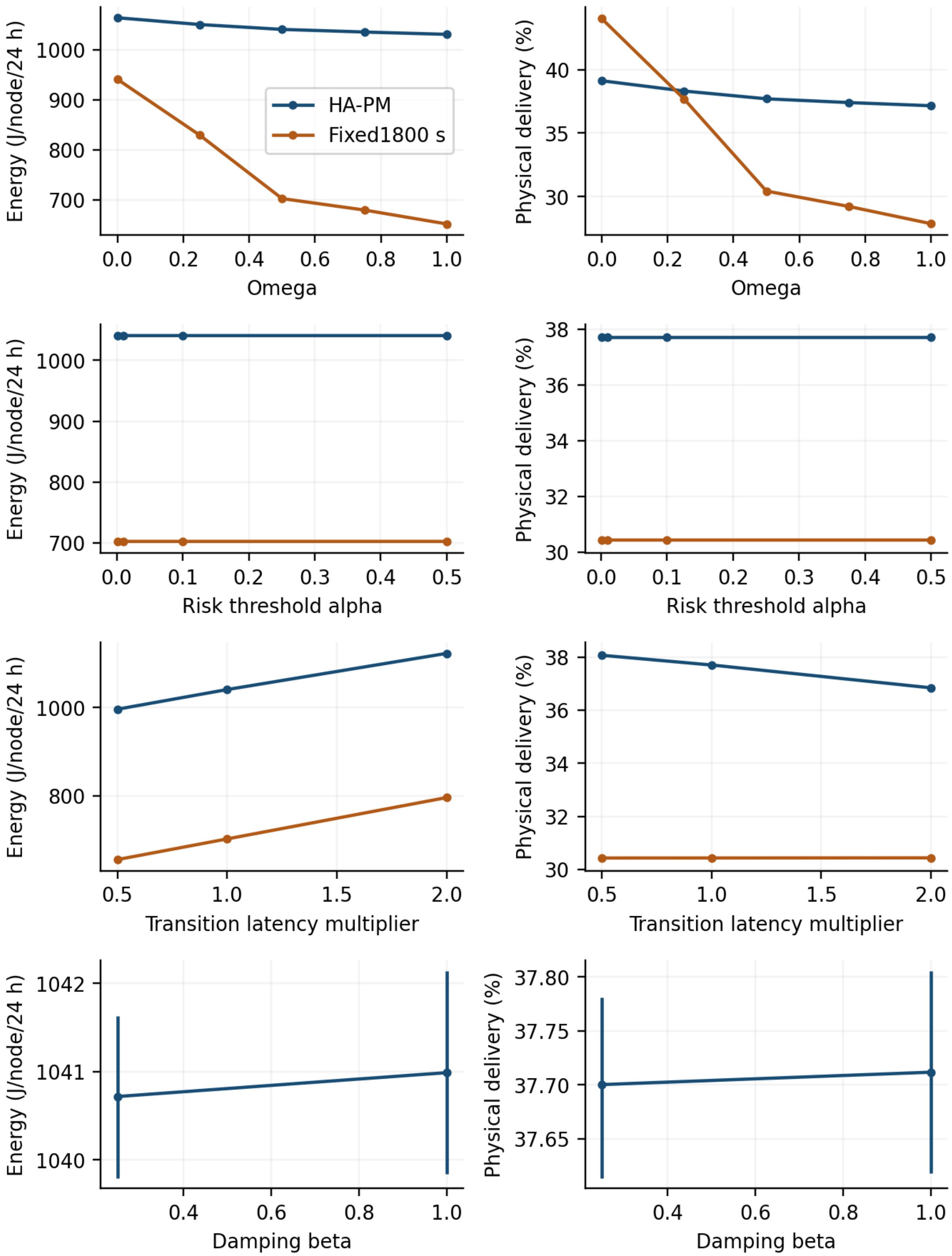

Figure S22. Eight panels pair energy and delivery for local weight, risk threshold, transition latency, and damping; pointwise exploratory 95% intervals across 30 traces.

S11.9 Environmental and channel sensitivity

Environmental sensitivity combines storage, harvesting, workload, and channel assumptions whose effects need not align. Figure S23 retains energy and physical delivery under changes in leakage current, clear-day net harvest, event rate, and packet erasure in the historical 24 h model. Increasing event rate reduces the delivered fraction in both displayed policies while changing the physical event population itself. Those workload comparisons consequently do not hold the eligible event set fixed.

The low-rate case, one event per 1800 s per node, exposes an adverse continuity result. HA-PM has 72.667% uncut nodes at 24 h, with a pointwise interval of [71.033, 74.300]%, and a restricted alive time of 23.832 h [23.822, 23.843]. Fixed 1800 s has no observed cutoff in this case. Thus nominal horizon survival does not imply survival under lighter event traffic. The channel panels additionally show decreasing delivery with erasure probability without converting lost events into an equal-service energy comparison. This historical evidence narrows the manuscript's environmental robustness interpretation. Factors change individually, intervals summarize 30 independent network traces, and the results provide no hardware calibration, universal operational-continuity guarantee, or parameter optimum.

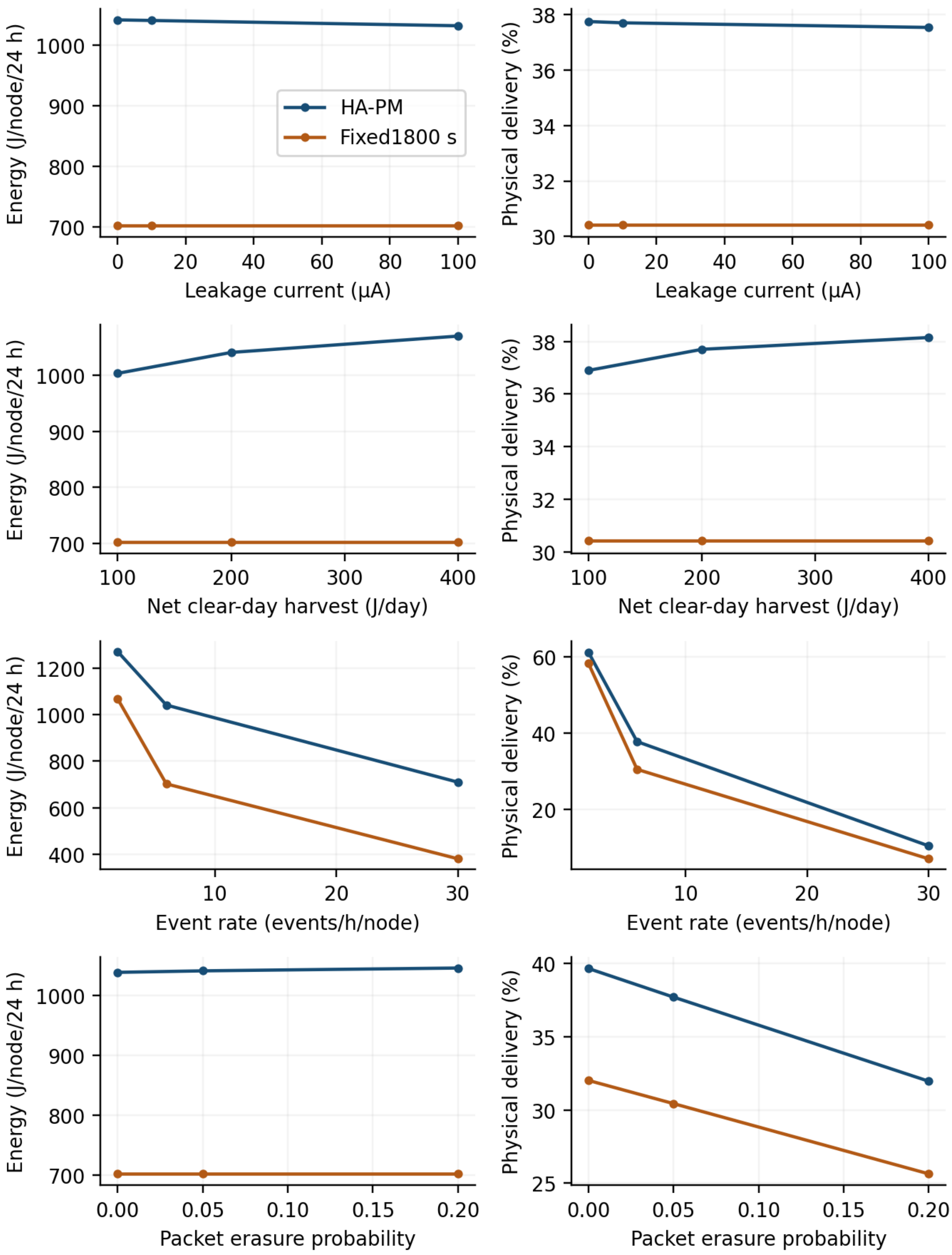

Figure S23. Eight panels pair energy and delivery for leakage, harvest, event-rate, and erasure changes; pointwise exploratory 95% intervals over 30 traces.

S11.10 Local-weight response under nominal and burst workloads

Local-weight sensitivity depends on the workload under which the selector operates. Figure S24 retains all five settings, 0, 0.25, 0.5, 0.75, and 1, for nominal and burst conditions in the historical 24 h model. Energy and physical delivery are shown for HA-PM and fixed 1800 s. The fixed reporting comparator still uses the declared local selection rule, so its outcomes can respond to the local weight.

Increasing the weight lowers the displayed energy for both policies, but also lowers delivery, especially for the fixed-period comparator. Under the burst workload, the fixed policy's delivery curve declines more steeply than HA-PM's; the energy curves alone would conceal that service cost. The source retains exact pointwise intervals and all trace values for these means. These joint responses clarify the manuscript's historical local-policy interpretation without selecting a universally preferred weight or inferring equal-service savings. This is an event-service model with realized transition and active consumption, not the separate superseded conditional cycle-energy blend. Its shared symbol therefore does not make the studies interchangeable. The 30 independent network traces define uncertainty, and the plotted nominal and burst means do not constitute an optimization over deployment distributions.

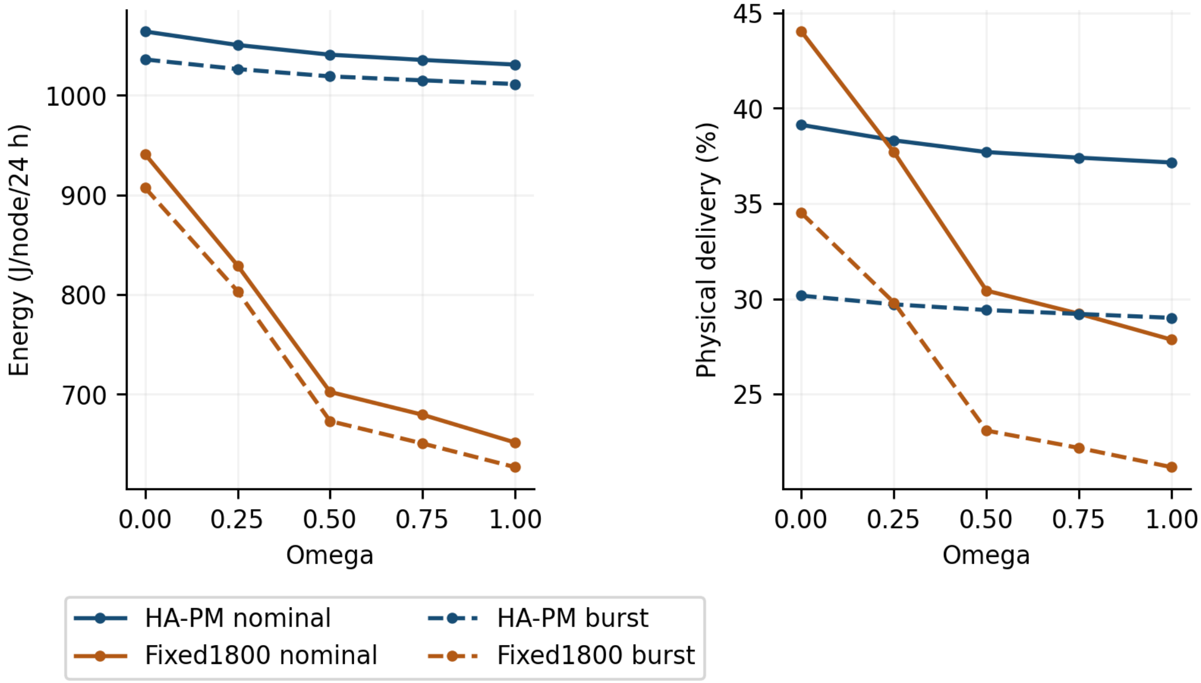

Figure S24. Nominal and burst-workload energy and delivery across five local weights; curves show means over 30 independent network traces.

S11.11 Censor-aware 24 h horizon outcomes

A finite observation horizon distinguishes first cutoff from continued operation. The displayed definition uses supercapacitor voltage $V_{SC}(t)$ and minimum operating voltage V_{min}, both in volts. Table S51 retains all 17 nominal, clamp, and stress-policy rows of the historical 24 h evaluation. All 3000 nominal fixed-300 s histories cross the threshold, yielding restricted alive time 21.490 h [21.469, 21.510]. Nominal HA-PM and the other fixed policies each have 3000 censored histories.

Figure S25 separates survival through 24 h from min(T, 24 h). A noncrossing history contributes 24 h to this restricted quantity, not an observed death time. All clamp and declared stress rows likewise lack a

first cutoff within the horizon and cannot rank uncensored lifetimes. A crossing exactly at the endpoint is observed. Operation stops at first cutoff without harvest-driven restart, while nodes and subsequent physical events remain in the common service denominator. These distinctions constrain the manuscript's historical lifetime interpretation and prevent multi-year extrapolation. Counts aggregate 100 nodes within each of 30 independent traces; resampling uses the trace, not 3000 independent node trials.

Table S51. Cutoff/censored counts and restricted alive time for all nominal, clamp, and declared stress conditions; pointwise exploratory 95% trace-bootstrap intervals.

Table S51		
**Condition / policy**	**Cutoff / censored nodes**	**Restricted alive time (h), mean [95% interval]**
Nominal fixed300s	3000 / 0	21.490 [21.469, 21.510]
Nominal fixed600s	0 / 3000	24.000 [24.000, 24.000]
Nominal fixed900s	0 / 3000	24.000 [24.000, 24.000]
Nominal fixed1800s	0 / 3000	24.000 [24.000, 24.000]
Nominal fixed3600s	0 / 3000	24.000 [24.000, 24.000]
Nominal HA PM full	0 / 3000	24.000 [24.000, 24.000]
Nominal HA PM soc fixed50percent	0 / 3000	24.000 [24.000, 24.000]
Nominal HA PM load fixed0.5	0 / 3000	24.000 [24.000, 24.000]
Nominal HA PM weather context fixed0.5	0 / 3000	24.000 [24.000, 24.000]
weather only HA PM full	0 / 3000	24.000 [24.000, 24.000]
weather only fixed1800s	0 / 3000	24.000 [24.000, 24.000]
gateway outage only HA PM full	0 / 3000	24.000 [24.000, 24.000]
gateway outage only fixed1800s	0 / 3000	24.000 [24.000, 24.000]
burst only HA PM full	0 / 3000	24.000 [24.000, 24.000]
burst only fixed1800s	0 / 3000	24.000 [24.000, 24.000]
combined HA PM full	0 / 3000	24.000 [24.000, 24.000]
combined fixed1800s	0 / 3000	24.000 [24.000, 24.000]

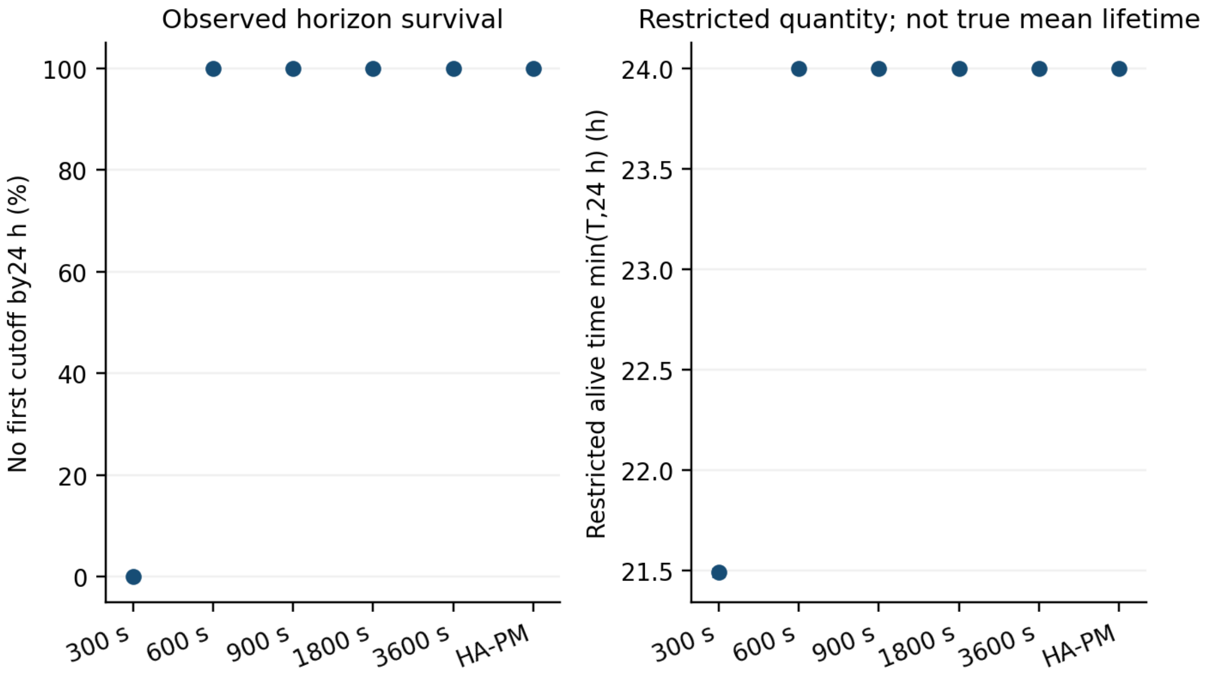

Figure S25. Horizon survival and restricted alive time for six nominal policies; pointwise exploratory 95% intervals over 30 network traces.

$$L_{op} = min\{t: V_{SC}(t) \leq V_{min}\} \quad (7)$$

S11.12 Device and service assumptions in the 24 h model

Table S52 defines the device and event-service assumptions of the historical 24 h network model. Independent physical events persist for 2 s, while a finite FIFO discards the newest observation on overflow. These choices determine which detected events can become delivered records, rather than merely setting reporting frequency.

Here P_i denotes mode power in watts, t_i transition time in seconds, and T_i the minimum idle-time threshold in seconds. Entry/exit splitting and ideal sensing availability are synthetic assumptions; the listed millisecond thresholds are policy guards rather than actual sleep durations. Aggregate M0 power covers active services without an extra component-current debit. These distinctions support interpretation of the manuscript's historical energy/service results without presenting adopted settings as hardware calibration.

Table S52. Historical device and service assumptions, retaining reference quantities and their source qualifications.

Table S52		
**Quantity**	**Value / units**	**Evidence / role**
Evaluation design	100 nodes; one gateway; 30 independent traces; H = 86400 s	Declared synthetic evaluation design
Physical events	Independent per-node Poisson; nominal rate 1/600 s^{-1}; persistence 2 s	Author-adopted workload; no geographic or collision model

Table S52		
**Quantity**	**Value / units**	**Evidence / role**
Queue and payload	FIFO, drop newest; 16 records; 64 bytes per record	Author-adopted service model
Active services	Processing 0.1 s; uplink 0.9 s; heartbeat receive window 0.25 s	Aggregate M0 power; no extra component-current debit
Mode powers	M0 1730; M15 300; M16 260; M17 110; M18 14; M19 0.7 mW	Published reference quantities: LPWAN source, Table 2
Mode thresholds	M15–M19: 7, 15, 30, 189, 300 ms	Author-retained policy convention; distinct from actual sleep
Transition model	Reported ti split equally into entry/exit; linear power ramps; zero extra impulse	Explicit synthetic adaptation; not measured split
Sensing availability	On in M0/M15–M18; off in M19 and transitions; continuous ideal visibility while on	Author-adopted capability model

S11.13 Control, storage and communication assumptions

The storage and transport settings in Table S53 define what the historical network controller can know and execute. Gateway observations, command generation, transmission, receipt, and node application occur on different clocks. Freshness and cache rules can therefore make the applied period differ from the generated output.

Physical net harvest is already storage-side input, and discarded overflow is recorded separately. Leakage follows evolving voltage under the declared constant-current assumption. The local strict risk gate, reserve constraints, output damping, and upward quantization belong to this 24 h model. These assumptions clarify the manuscript's historical implementation context while remaining separate from the current replacement selector and from numerical integration cadence.

Table S53. Historical local-control, storage, and communication assumptions, including their model and accounting boundaries.

Table S53		
**Quantity**	**Value / units**	**Evidence / role**
Local selection	$\omega = 0.5$; strict qi < α with $\alpha = 0.1$; rate window 600 s; zero-rate watchdog 60 s	Author-adopted local policy; no division by zero
Storage	C = 270 F; Vmax = Vinitial = 3.8 V; Vmin = 2.5 V	Published rating plus declared initialization/cutoff scenario

Table S53		
**Quantity**	**Value / units**	**Evidence / role**
Leakage	Ileak = 10 μA; Pleak(t) = Ileak V(t)	Synthetic constant-current law; evolving voltage integrated
Net harvest	Clear-day area 200 J; positive half-sine 06:00–18:00; physical weather multiplier	Already net storage input; overflow recorded separately
Packet loss	Independent erasure probability 0.05; no retransmission	Abstract channel assumption; no LoRaWAN MAC validation
Synchronization bounds / fallback	60–3600 s; adaptive initial/default interval 1800 s; fixed policies retain their fixed interval	Timing-design assumptions; no claimed regulatory guarantee
Physical monitoring / control	Gateway sampling and FLC period 60 s; received-load window 600 s	Distinct from numerical integration substeps
Freshness / dissemination	Stale SoC 1200 s; node cache 3600 s; command TTL 3600 s; gate 30 s / 600 s	Sample, generation, actual transmission and valid-receipt times are distinct
Output processing	Separate Low-SoC envelope; protected first-order β = 0.25; upward 1 s quantization	Explicit adopted implementation; prior computed output state

S11.14 Membership, stress and analysis settings

Table S54 completes the historical configuration with membership knots, stress timing, comparison policies, and statistical settings. The received-load definition uses observed packets; weather context describes sampled information rather than future physical forcing. Original consequents and the piecewise-linear centroid remain part of the declared mapping.

The three single-input clamps preserve the same local service model and paired exogenous conditions. The common clock preserves scheduled stress timing across paired policies. Numerical step refinement controls storage integration accuracy without changing the sensing or command cadence. Statistical resampling treats the whole network trace as independent, with pointwise exploratory intervals and no confirmatory superiority claim. The source declares 61 conditions and 87 comparisons, each condition using 30 network traces. Their complete raw tables were not independently reread or recomputed here. These settings bound the manuscript's historical interpretation without implying empirical calibration.

Table S54. Historical membership, stress, numerical, and statistical assumptions with their stated interpretation limits.

Table S54		
**Quantity**	**Value / units**	**Evidence / role**

Table S54		
**Quantity**	**Value / units**	**Evidence / role**
SoC memberships (x, μ)	Low: (0,1),(35,0); Medium: (20,0),(50,1),(80,0); High: (65,0),(90,1),(100,1)	Author-adopted piecewise-linear knots; percentage universe
Load memberships (x, μ)	Low: (0,1),(.4,0); Normal: (.2,0),(.5,1),(.8,0); High: (.6,0),(1,1)	Load = clip(received count / (600 s × 1 packet s^{-1}),0,1)
Weather memberships (x, μ)	Poor: (0,1),(.4,0); Average: (.25,0),(.5,1),(.75,0); Good: (.6,0),(1,1)	Dimensionless sampled context; distinct from future physical weather
Output memberships (s, μ)	Short: (60,1),(200,1),(1200,0); Medium: (800,0),(1800,1),(2800,0); Long: (2400,0),(3600,1)	27 original consequents retained; exact piecewise-linear centroid
Stress schedule	Weather 0.2 during 6–12 h; gateway outage 8–14 h; rate 1/120 s^{-1} during 10–12 h	Separate weather/outage/burst and combined scenarios
Comparison / ablation	Fixed 300/600/900/1800/3600 s; full HA-PM; SoC=50%, load=.5, weather=.5 single-input ablations	Same exogenous trace and local device/service model within each pair
Numerical integration	Event-driven physical clock; maximum storage substep 1 s; refinement 1/.5/.25 s	Numerical accuracy control; never a physical sensing cadence
Statistical unit	Whole-network trace; paired resampling; 2000 bootstrap draws; pointwise 95% intervals	Exploratory uncertainty only; no confirmatory superiority claim

S11.15 Storage-side energy accounting in the 24 h model

The historical 24 h storage model links actual service expenditure to a changing supercapacitor voltage. The displayed equations define usable state of charge, integrated device energy, the storage balance, leakage expenditure, and voltage recovery from stored energy. Usable state of charge is clipped to 0–100% and sampled at uplink transmission start, after the 0.1 s processing phase. A gateway therefore receives a timestamped observation rather than the evaluator's instantaneous state.

Aggregate device power includes realized mode residence, transitions, and active service, with power in watts and time in seconds. Every term in the storage balance covers the same integration interval, ending no later than the first operational cutoff. Harvest is already net input at the storage boundary; overflow discarded at maximum energy is counted once. The numerical integration step controls the energy calculation and does not prescribe sensing, control, synchronization, or packet cadence.

The leakage parameter I_{leak} is current in amperes; its constant value within a simulation yields voltage-dependent leakage power. Component selection, temperature, aging, and implementation motivate treating it as an explicit parameter. This historical model differs from the current campaign's proportional-energy leakage law. The 0.7 mW M19 reference corresponds arithmetically to 60.48 J over uninterrupted 24 h residence, excluding sensing. It is neither measured device consumption nor expenditure after an earlier cutoff. These definitions delimit the manuscript's storage and lifetime interpretation.

$$S_{SoC}(t) = 100 \cdot \frac{E_{SC}(t) - E_{min}}{E_{max} - E_{min}} \tag{8}$$

$$\mathrm{E_{dev}}[\mathrm{t_0}, \mathrm{t_1}] = \int_{\mathrm{t_0}}^{\mathrm{t_1}} \mathrm{P_{device}}(\mathrm{s})\, \mathrm{ds} \tag{9}$$

$$\mathrm{E_{SC}}(\mathrm{t}+\mathrm{h}) - \mathrm{E_{SC}}(\mathrm{t}) = \int_{\mathrm{t}}^{\mathrm{t+h}} \mathrm{P_{net}}(\mathrm{s})\, \mathrm{ds} - \mathrm{E_{dev}} - \mathrm{E_{leak}} - \mathrm{E_{overflow}} \tag{10}$$

$$\mathrm{E_{leak}}[\mathrm{t}, \mathrm{t}+\mathrm{h}] = \int_{\mathrm{t}}^{\mathrm{t+h}} \mathrm{I_{leak}} \mathrm{V_{SC}}(\mathrm{s})\, \mathrm{ds} \tag{11}$$

$$V_{SC}(t) = \sqrt{\frac{2E_{SC}(t)}{C}} \tag{12}$$

S12 Bounded-Input Numerical Replay

S12.1 Bounded numerical properties and their limits

The historical numerical model supports boundedness statements with distinct scopes. Table S55 retains the raw-output bound, projected storage-domain invariance, same-input Low-SoC non-regression, and reachable-mode contract. Its projection constrains computed energy to 0–1949.4 J; it does not guarantee operation above the 843.75 J cutoff.

The displayed equations separately express the memoryless mapping, storage recurrence, fixed-complete-input invariance, and reachable-M19 energy derivative. Projection is applied after each numerical storage update. Fixed exogenous context alone does not fix SoC in a storage-feedback replay. The negative derivative applies only to positive periods in the transition-disabled M19 energy model. These distinctions qualify the manuscript's controller interpretation: bounded output amplitude does not bound indefinite cumulative variation, and numerical projection does not establish physical safety or dynamic convergence.

Table S55. Four numerical properties of the historical model and their permitted and prohibited interpretations.

Table S55			
**Property**	**Verified bound/result**	**Permitted interpretation**	**Prohibited interpretation**
Raw FLC output bound	60 <= T_sync <= 3600 s	Bounded output for admissible finite inputs	Not asymptotic stability, ISS, convergence, or anti-chattering
Projected storage-domain invariance	0<=E<=1949.4 J; 0<=V<=3.8 V; 0<=usable SoC<=100%	Numerical projection/domain invariant	Not hardware safety, physical validation, or reliability

Table S55			
**Property**	**Verified bound/result**	**Permitted interpretation**	**Prohibited interpretation**
Low-SoC non-regression	T_safe >= T_nominal at identical input triple	Implemented local non-regression property	Not global monotonicity in SoC
EH-DPM reachable-mode contract	Contextwise/global knot minima and maxima recorded; all audited knots select M19; linear interpolation preserves knot endpoint bounds	Verified implementation-domain selector property and falsification check	Zero is not empirical anti-chattering or a hardware switching result

$$T_{sync,k} = F(S_k, N_k, W_k) \quad (13)$$

$$E_{k+1} = \Pi_{[0,Emax]}\{E_k + h_k[H_k - G(T_{sync,k})] - I_{leak}\, V(E_k) D h_k\} \quad (14)$$

$$z_k = z^* \; for \; every \; k \;\; \Rightarrow \;\; T_{sync,k} = F(z *) \; for \; every \; k \quad (15)$$

$$G_{M19}(T) = 60.48 + 298{,}823.04/T \; J \; \text{day}^{-1} \quad (16)$$

$$dG_{M19}(T)/dT = -298{,}823.04/T^2 < 0 \quad (17)$$

S12.2 Prespecified bounded-input replay design

The historical bounded-input replay evaluates the unchanged numerical controller over 14 days. Each replicate uses 2800 evaluations at 0.005-day steps and 112 external-input epochs of 0.125 day. Table S56 retains two stochastic scenarios with 200 replicates each, together with one fixed-context and one deterministic alternating replay. The prescribed probabilities and finite states define synthetic stresses rather than measured environmental distributions.

At each evaluation, current projected usable SoC and the external traffic/weather context enter the unchanged lookup. Raw periods remain unrounded before the daily-energy and mode-selection calculations. The wrapper introduces no command transport, output hold, quantizer, deadband, hysteresis, or dwell rule. A fixed external context can still produce changing complete input through storage feedback. Conversely, the alternating challenge deliberately forces persistent external variation and is not an autonomous limit-cycle experiment. Full-horizon diagnostics include all 14 days, potentially including numerical evaluations after the first 2.5 V crossing; separate pre-cutoff summaries answer another question. The modeled cutoff corresponds to 843.75 J of stored energy. This design supports a bounded numerical interpretation relevant to the manuscript, without converting computed evaluations into physical operating events or claims of field reliability.

Table S56. Prespecified fixed, moderate, adverse, and alternating input designs; counts refer to complete 14-day numerical replays.

Table S56			
**Scenario**	**Type**	**Replicates**	**Input design**

Table S56			
**Scenario**	**Type**	**Replicates**	**Input design**
Fixed exogenous context	fixed	1	N=0.50,W=0.50,H=230.5 throughout
Moderate stochastic	stochastic	200	weather pi=0.2|0.7031539888682746|0.09684601113172542; weather refresh=0.1; traffic p_on=0.08, p_off=0.5
Adverse stochastic	stochastic	200	weather pi=0.7|0.25|0.05; weather refresh=0.03; traffic p_on=0.2, p_off=0.2
Alternating challenge	alternating	1	even epochs adverse N=0.90,W=0.10,H=100; odd epochs good N=0.20,W=0.85,H=500

S12.3 Raw-output variation across stochastic replays

Raw-output variation measures numerical responsiveness rather than communication activity. Table S57 retains all four historical replay scenarios, while Figure S26 shows distributions across 200 replicates of each stochastic scenario. Moderate and adverse inputs yield mean total variation of 1303.43 and 1360.37 s/day, respectively. At the intermediate 60 s threshold, material changes occur at 1.24 and 1.32 numerical evaluations per day.

The figure also preserves the 1 s and 300 s thresholds, so interpretation does not depend on selecting one favorable change definition. The alternating challenge has substantially larger total variation, 17913.42 s/day, and 7.93 changes per day at 60 s. Even fixed exogenous context permits output variation because SoC remains dynamic. Immediate consecutive material reversals are a narrower diagnostic: zero counts do not exclude opposite increments separated by unchanged evaluations. These findings qualify the manuscript's boundedness discussion by reporting sustained variation rather than relabeling it convergence. All quantities use the complete 14-day numerical horizon and may include post-crossing evaluations. They are not broadcasts, applied command updates, physical mode switches, or rates of operation before cutoff.

Table S57. Full-horizon raw-period means, ranges, total variation, and 60 s material-change rates for four replay scenarios.

Table S57				
**Scenario**	**Mean T_sync (s)**	**Mean range (s)**	**Total variation (s/day)**	**|Delta T_sync| >= 60 s (numerical evaluations/day)**
Fixed exogenous context	1,675.38	1,354.90	96.78	0.00

Table S57				
**Scenario**	**Mean T_sync (s)**	**Mean range (s)**	**Total variation (s/day)**	**|Delta T_sync| >= 60 s (numerical evaluations/day)**
Moderate stochastic	1,777.06 (95% interval 1,743.81-1,810.65)	2,488.94 (95% interval 2,449.82-2,527.08)	1,303.43 (95% interval 1,260.39-1,347.58)	1.24 (95% interval 1.20-1.28)
Adverse stochastic	2,324.28 (95% interval 2,273.22-2,371.45)	2,057.58 (95% interval 1,974.18-2,139.37)	1,360.37 (95% interval 1,303.64-1,415.89)	1.32 (95% interval 1.27-1.37)
Alternating challenge	1,778.04	2,663.30	17,913.42	7.93

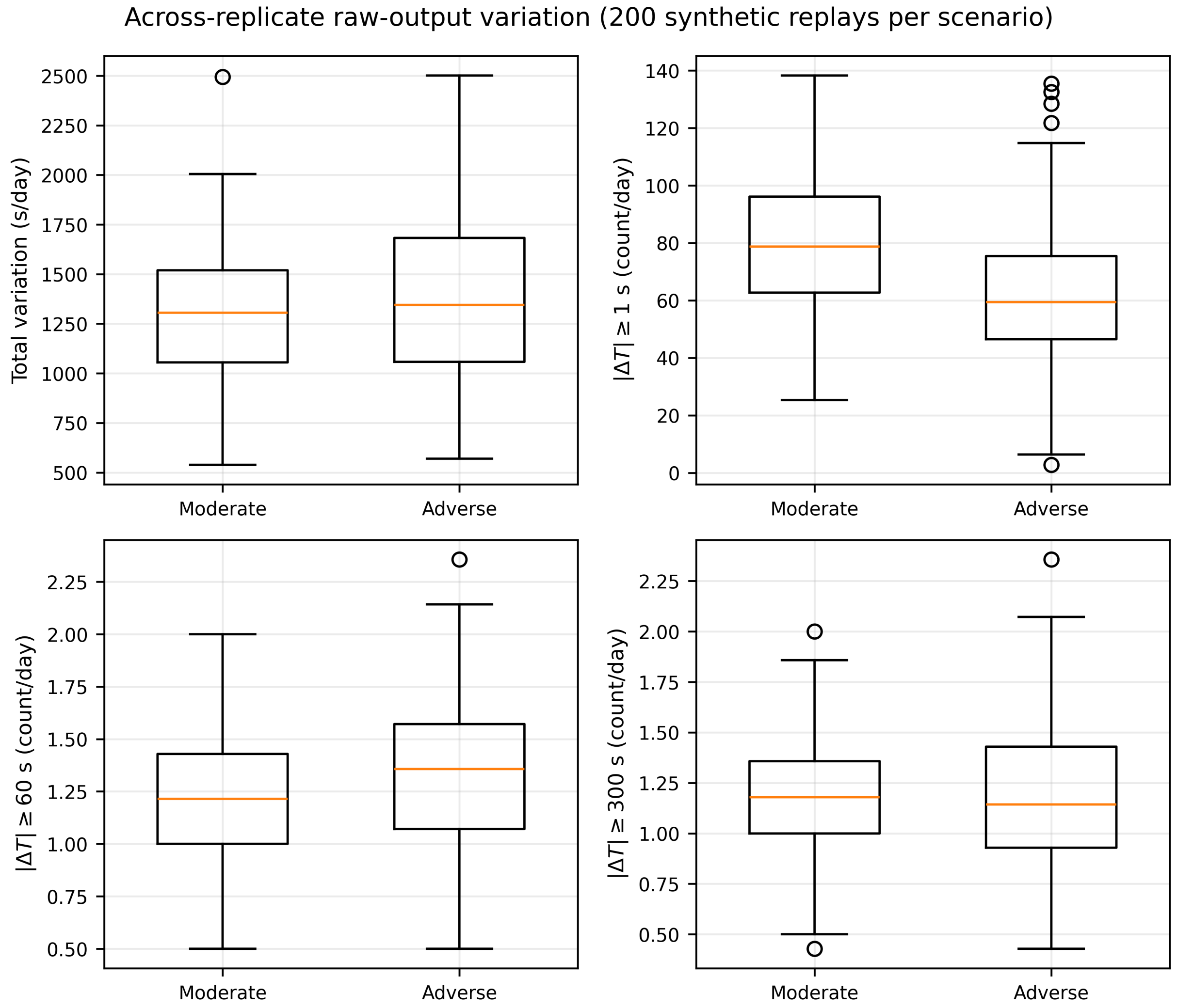

Figure S26. Stochastic replicate distributions of total variation and material-change rates at 1, 60, and 300 s; outliers remain visible.

S12.4 Projected-storage threshold diagnostics

Projected-storage threshold outcomes distinguish an event fraction from a restricted first-passage time. Table S58 retains all four historical replay scenarios, including immediate-reversal and mode-label diagnostics. Figure S27 reports the two 200-replicate stochastic cases. Moderate inputs produce 7.50% crossings, with a Wilson 95% interval of [4.60, 12.00]%, and a mean 14-day restricted first passage of 13.72 days [13.56, 13.87]. Adverse inputs give 60.50% [53.59, 67.02]% and 12.51 days [12.29, 12.72].

The restricted means use percentile-bootstrap intervals, a different construction from the crossing intervals. A noncrossing replicate contributes 14 days and remains right-censored rather than acquiring an observed failure time. The deterministic fixed and alternating cases have no crossing within the horizon. All scenario mode-label switch counts are zero because the audited lookup domain selects M19; they do not demonstrate physical anti-chattering. These conditional ideal-storage outcomes delimit the manuscript's numerical behavior claims. They depend on the projected recurrence, synthetic harvesting, leakage assumption, and finite horizon, and provide neither hardware lifetime estimates nor an energy-neutrality guarantee.

Table S58. Full-horizon reversal, mode-label, threshold-crossing, and restricted first-passage diagnostics for all four replay scenarios.

Table S58

Scenario	Immediate consecutive reversals >= 60 s (numerical evaluations/day)	Mode-label switches (count)	Threshold crossing fraction	Mean 14-day restricted first passage (day)
Fixed exogenous context	0.00	0.00	0.00%	14.00
Moderate stochastic	0.00 (95% interval 0.00-0.00)	0.00 (95% interval 0.00-0.00)	7.50% (95% interval 4.60-12.00%)	13.72 (95% interval 13.56-13.87)
Adverse stochastic	0.00 (95% interval 0.00-0.00)	0.00 (95% interval 0.00-0.00)	60.50% (95% interval 53.59-67.02%)	12.51 (95% interval 12.29-12.72)
Alternating challenge	0.00	0.00	0.00%	14.00

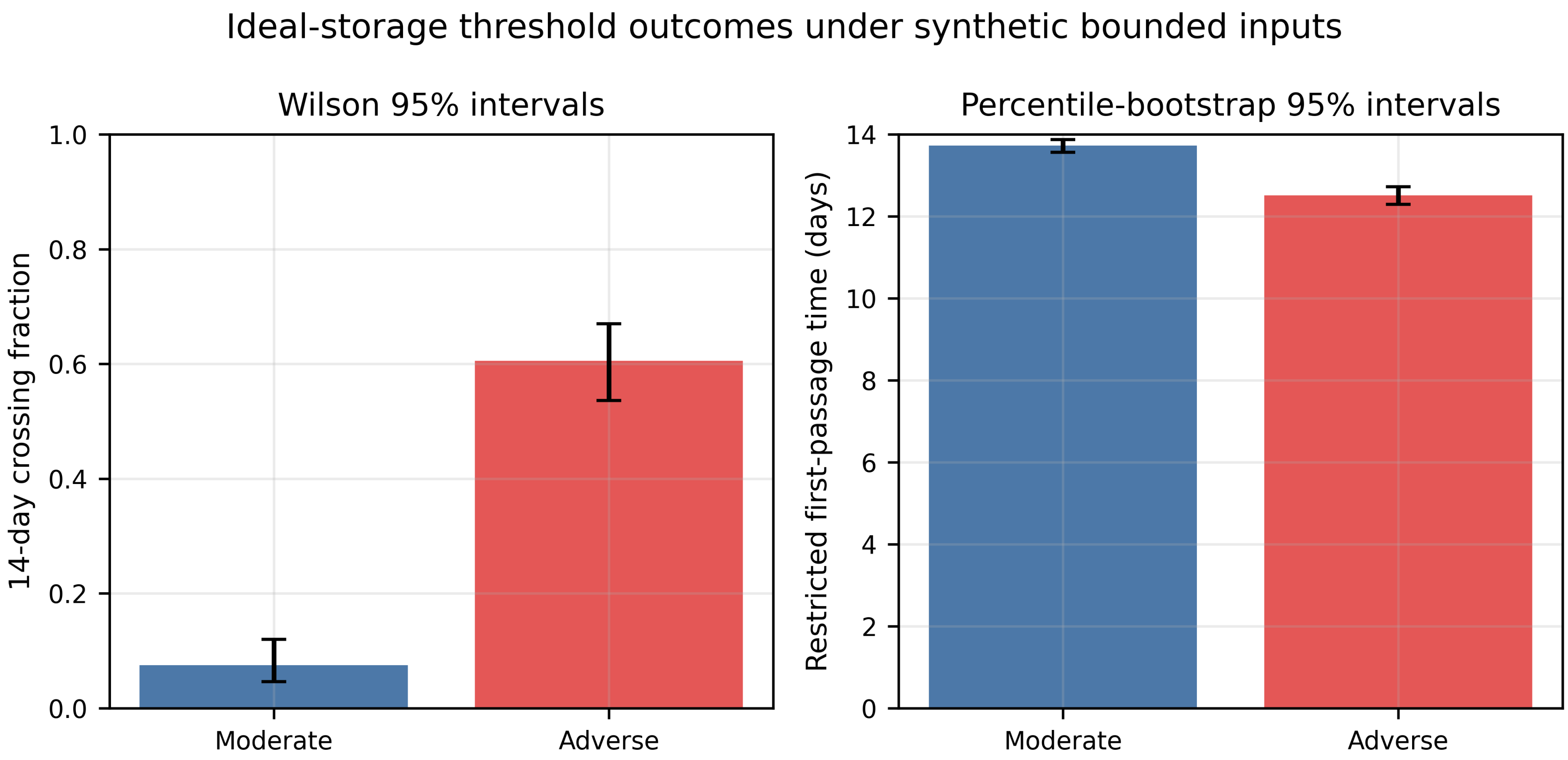

Figure S27. Modeled crossing fractions and restricted first-passage means for moderate and adverse inputs, with their distinct 95% interval constructions.

S12.5 Externally forced alternating replay

The deterministic alternating replay challenges the controller with persistent bounded external variation. Figure S28 retains context, harvesting, raw period, usable SoC, and computed voltage over the entire 14-day horizon. Even epochs use adverse traffic/context and 100 J/day harvesting; odd epochs use favorable inputs and 500 J/day. The changing complete input includes both this imposed sequence and evolving storage.

Raw periods span 516.70–3180.00 s and continue to vary rather than converging to a constant. The modeled operational threshold is not crossed in this single replay, while the mode-label switch count is zero. The latter follows the M19-only reachability of the audited domain despite large changes in period; it is not a measured dwell-time or switching guarantee. The unsmoothed traces retain the response rather than concealing it through an added temporal mechanism. This example therefore supports the manuscript's distinction between bounded amplitude and convergence. It demonstrates an externally forced numerical response, not an autonomous limit cycle, a physical voltage waveform, or a global stability proof. One deterministic challenge also cannot supply stochastic confidence intervals or establish reliability across possible environmental sequences.

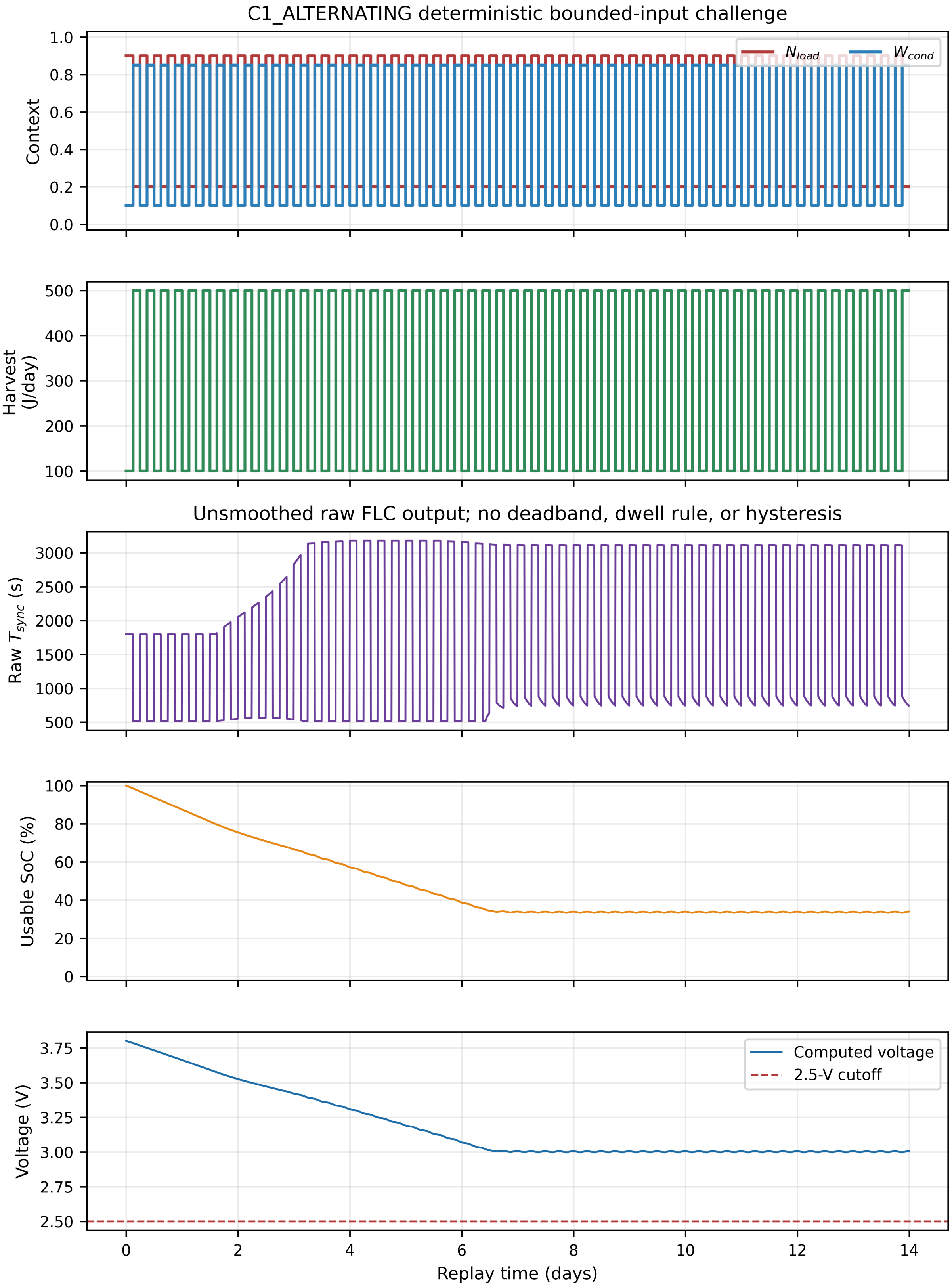

Figure S28. Five unsmoothed panels show imposed context and harvest, raw period, usable SoC, and computed voltage in the alternating replay.

S13 Deterministic Prolonged Outage Study

S13.1 Primary deterministic prolonged-outage case

The historical prolonged-outage abstraction compares connected operation, indefinite hold-last, and timeout fallback under identical storage conditions. Figure S29 shows the predeclared 72 h outage and 96 h observation window. A valid 445.104 s command is cached before outage; receptions fail until restoration, and the first eligible later command succeeds by assumption. Fallback is applied at a missed synchronization opportunity rather than exactly when its timeout expires.

Indefinite hold-last crosses 2.5 V at 52.35 h. With a 12 h timeout and 3600 s fallback, activation occurs at 12.117 h, and the trajectory has no crossing through 96 h. At outage end, modeled voltage is 3.746 V with 95.03% usable SoC; command recovery occurs at 72.117 h. Both cached and fallback periods select M19, so fewer synchronization cycles explain the benefit. This conditional result supports a narrower outage mechanism relevant to the manuscript. It excludes event-service, RX, transition, converter/ESR, temperature, aging, and drift costs; the 230.5 J/day harvest assumption remains provisional. Consequently, modeled recovery and noncrossing do not establish physical fault tolerance.

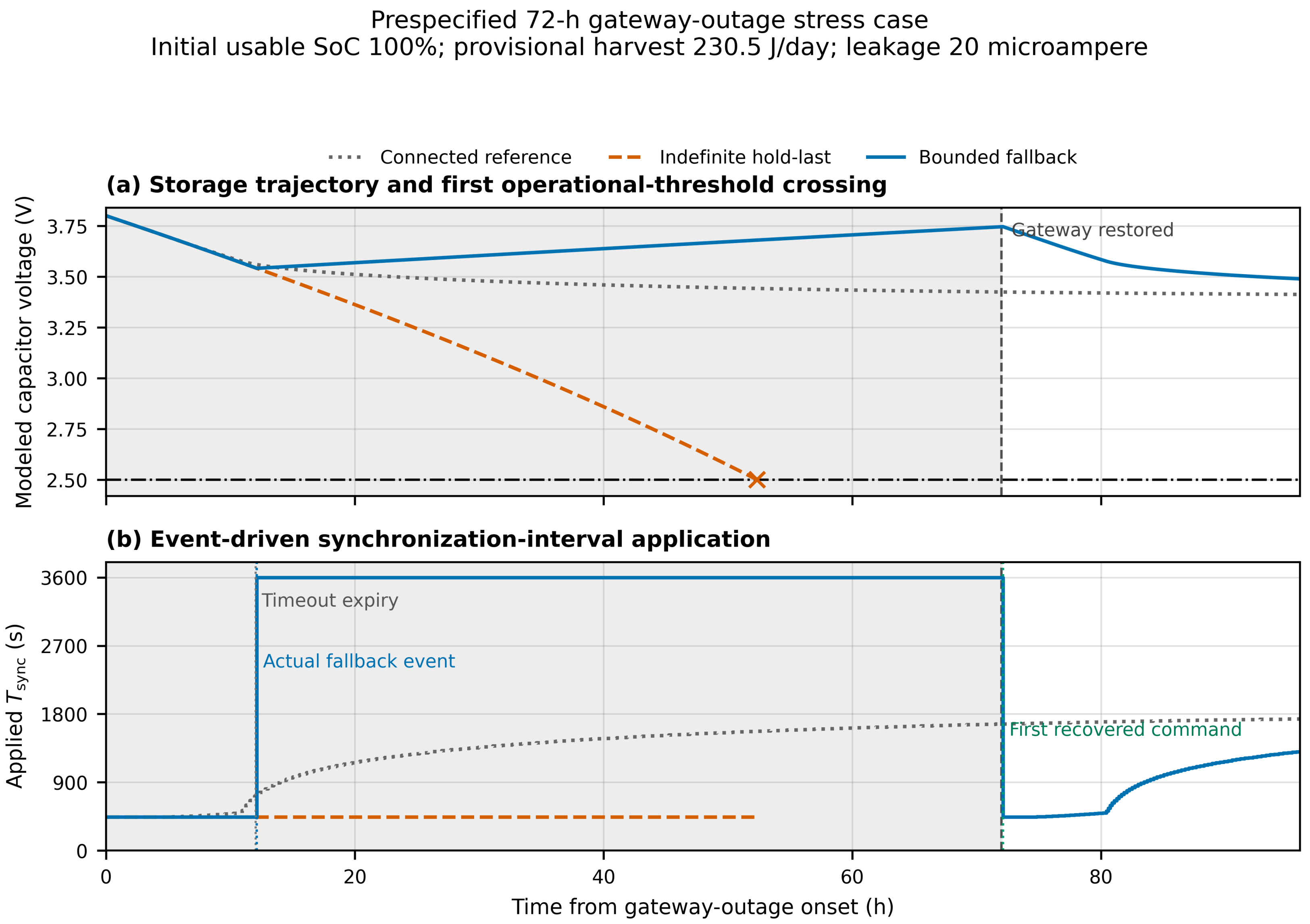

Figure S29. Connected, hold-last, and fallback trajectories: modeled storage voltage and applied interval, with outage, threshold crossing, fallback, and command-recovery annotations.

S13.2 Complete deterministic outage domain

The complete deterministic outage domain tests whether the primary-case benefit persists across declared conditions. It crosses three initial SoC levels, four network/weather contexts, five harvest assumptions, three leakage levels, and four outage durations, yielding 720 physical scenarios and 2160 policy runs. Figure S30 retains the paired hold-last/fallback comparison and the primary-case design sweep.

During outage, indefinite hold-last crosses the operational threshold in 290 scenarios, compared with 128 under bounded fallback. Fallback avoids 162 crossings observed under hold-last; threshold-free outage time is longer in 290 cases, equal in 430, and shorter in none. These frequencies describe the chosen deterministic domain, not sampling probabilities. The 430 coincident normalized points remain explicitly identified. The design sweep also retains failure: a 300 s fallback crosses at every tested timeout, and a 600 s fallback crosses with the 24 h timeout. Thus both timeout and interval affect the outcome. This historical evidence qualifies the manuscript's fallback interpretation without claiming universal drain prevention. Its duty-cycle-averaged accounting and omitted service/radio/physical losses retain the limitations of the primary case, and noncrossing outcomes are censored at outage duration plus 24 h.

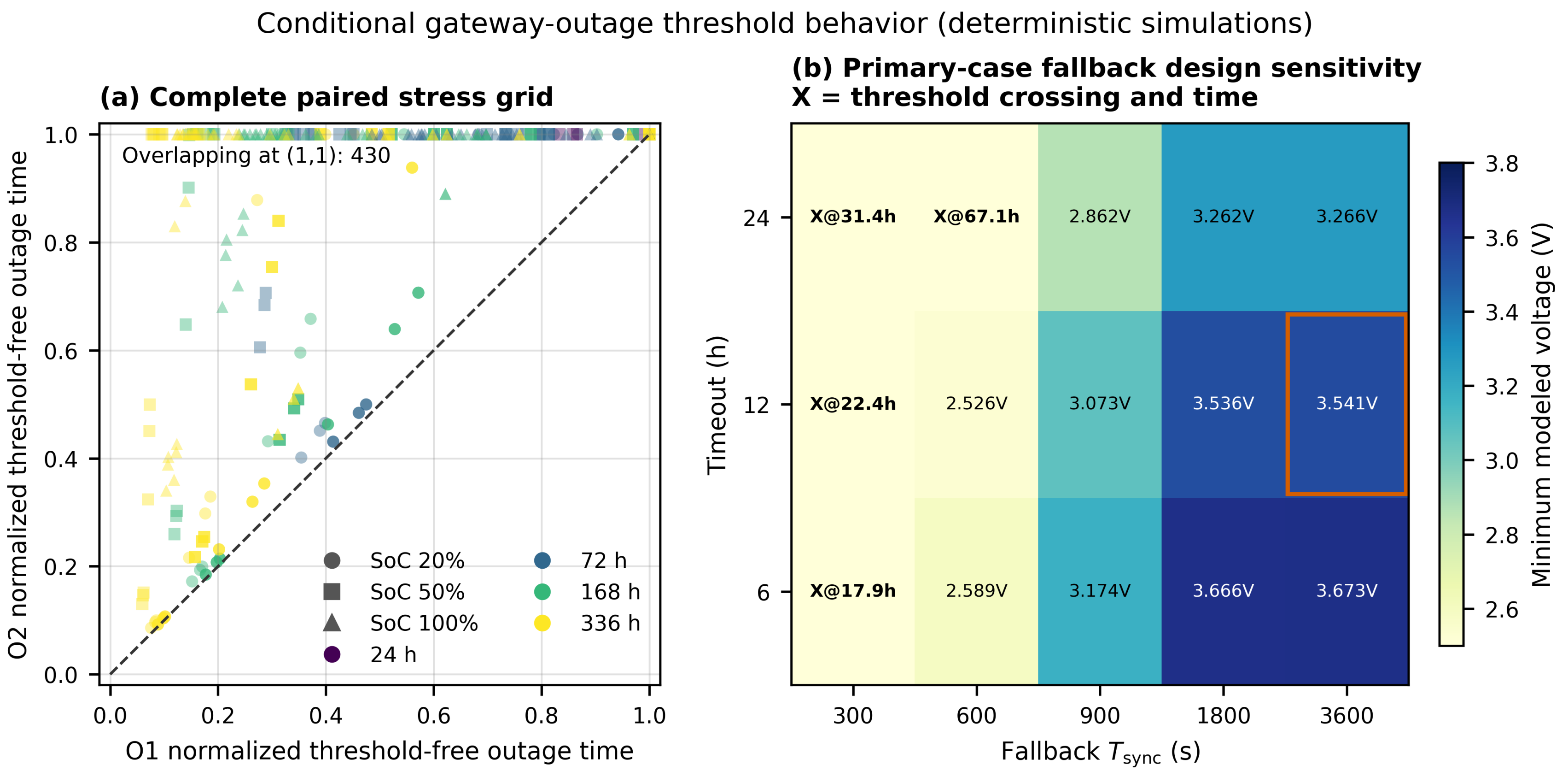

Figure S30. Complete paired threshold-free outage durations and primary-case timeout/fallback sensitivity; colors show minimum modeled voltage, and X marks crossings.

S14 Provisional Thermal Diagnostic

The separate thermal diagnostic is provisional. Figure S31 retains voltage, usable SoC, and selected-mode histories over 72 h, with a 12–48 h gateway outage and prescribed 15–35 °C variation. Its thermal integration and terminal-voltage calculation belong to this branch rather than the current campaign's physical kernel.

Table S59 preserves the six reported energy quantities and their harvest-normalized percentages. Gross load is 7671.90 J, leakage 496.67 J, ESR loss 30.95 J, and discarded harvest 49459.45 J. The reported stored-energy change is −884.52 J. The associated 7.63 J balance discrepancy remains unresolved; no new correction term is introduced to force closure.

The baseline's apparent operating margin is also indistinguishable from zero: its sign changes under a twice-coarser timestep and its magnitude shrinks under refinement. This numerical sensitivity prevents interpreting the plotted trace as robust survival. The diagnostic therefore qualifies the manuscript's physical-model discussion without supporting hardware reliability, a validated energy-neutral regime, or superiority to external adaptive methods. Its retained negative accounting and convergence findings are integral to the interpretation.

Table S59. Uncorrected thermal energy ledger: six quantities and their percentages of gross harvest.

Table S59		
**Audit Metric**	**Energy Quantity (J)**	**Proportion of Harvest (%)**
Total Photovoltaic Energy Harvested (E_harv)	56,766.82	100.00%
Gross Load Energy Consumption (E_load)	7,671.90	13.51%
Photovoltaic Overcharge Curtailment (E_spill)	49,459.45	87.13%
Arrhenius Thermal Leakage Loss (E_leak)	496.67	0.87%
Internal ESR Ohmic Dissipation (E_esr)	30.95	0.05%
Net Capacitor Energy Delta (ΔE_stored)	-884.52	-1.56%

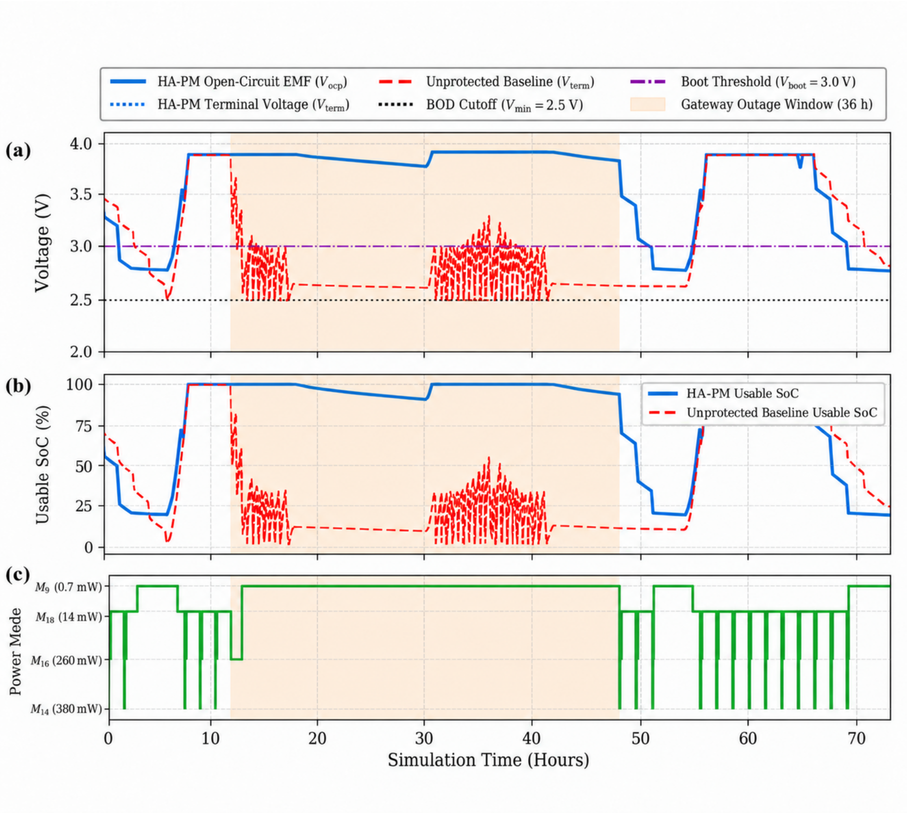

Figure S31. Provisional thermal voltage, usable-SoC, and mode histories with outage and numerical-margin annotations.

S15 Energy Consumption Distributions and Variability

HA-PM occupies the lowest region of per-duty-cycle energy among the evaluated reporting policies and has the smallest reported absolute standard deviation (32.9 mJ) (Figure S32 and S33). Relative to the reported standard deviations for fixed 1 h and fixed 1 min operation, this corresponds to reductions of 69.5% and 92.1%, respectively. These reductions describe absolute dispersion rather than mean energy savings. The figure therefore supports a descriptive advantage in cycle-energy magnitude and variability for the displayed comparison. Its reported Wilcoxon significance requires the underlying pairing and test records before being treated as independently verified evidence.

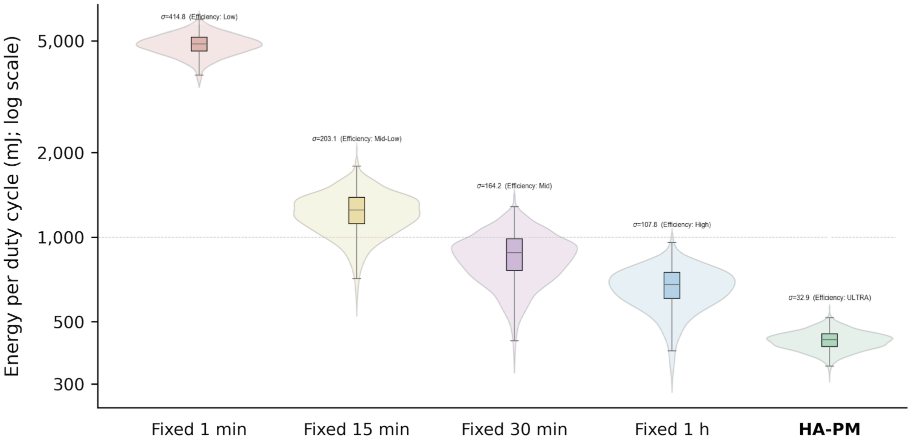

Figure S32. Energy distributions: A source-artwork violin comparison on a logarithmic energy axis.

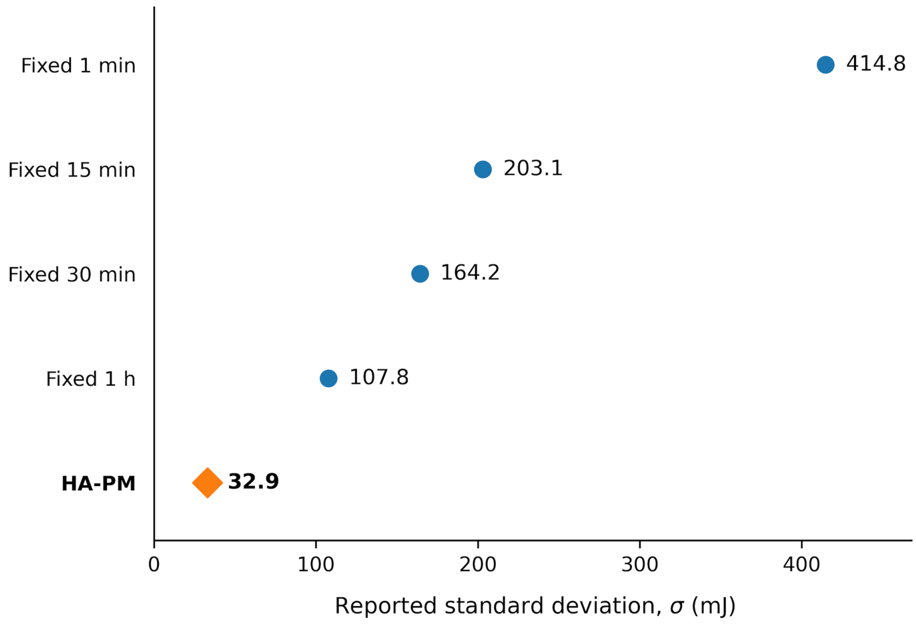

Figure S33. Absolute variability of energy per duty cycle.
